# Predictors of major adverse cardiac and cerebrovascular events after percutaneous coronary intervention in older adults: a systematic review and meta-analysis

**DOI:** 10.1186/s12877-024-04896-4

**Published:** 2024-04-12

**Authors:** Arash Jalali, Ali Hassanzadeh, Mohammad Sadeq Najafi, Sepehr Nayebirad, Mohadese Dashtkoohi, Zahra Karimi, Akbar Shafiee

**Affiliations:** 1 Tehran Heart Center, Cardiovascular Diseases Research Institute, Tehran University of Medical Sciences, North Kargar Ave, 1411713138 Tehran, Iran; 2 Department of Epidemiology and Biostatistics, School of Public Health, Tehran University of Medical Sciences, Tehran, Iran; 3 Diabetes Research Center, Endocrinology and Metabolism Clinical Sciences Institute, Tehran University of Medical Sciences, Tehran, Iran; 4 Research Center for Advanced Technologies in Cardiovascular Medicine, Cardiovascular Diseases Research Institute, Tehran University of Medical Sciences, Tehran, Iran; 5 Vali-E-Asr Reproductive Health Research Center, Family Health Research Institute, Tehran University of Medical Sciences, Tehran, Iran

**Keywords:** Percutaneous coronary intervention, Older adults, Coronary artery disease, Major adverse cardiac events

## Abstract

**Aim:**

We systematically reviewed and meta-analyzed the predictors of major adverse cardiac and cerebrovascular events (MACE/MACCE) in older adults who underwent PCI.

**Methods:**

Three databases, PubMed, Embase, and Scopus, were searched for observational studies considering the out-of-hospital MACE/MACCE in adults ≥ 60 years old with coronary artery disease (acute or chronic) who underwent PCI. Studies were eligible if they had determined at least two statistically significant predictors of MACE/MACCE by multivariable analysis. We used the QUIPS tool to evaluate the risk of bias in the studies. Random-effects meta-analysis was utilized to pool the hazard ratios (HRs) of the most reported predictors.

**Results:**

A total of 34 studies were included in the review. Older age (HR = 1.04, 95% Confidence Interval (CI): 1.03–1.06, *P*-value < 0.001), diabetes (HR = 1.36, 95% CI: 1.22–1.53, *P* < 0.001), history of myocardial infarction (MI) (HR = 1.88, 95% CI: 1.37–2.57, *P* < 0.001), ST-elevation MI (STEMI) at presentation (HR = 1.72, 95% CI: 1.37–2.18, *P* < 0.001), reduced left ventricular ejection fraction (LVEF) (HR = 2.01, 95% CI: 1.52–2.65, *P* < 0.001), successful PCI (HR = 0.35, 95% CI: 0.27–0.47, *P* < 0.001), eGFR (HR = 0.99, 95% CI: 0.97-1.00; *P*-value = 0.04) and left main coronary artery (LMCA) disease (HR = 2.07, 95% CI: 1.52–2.84, *P* < 0.001) were identified as predictors of MACE.

**Conclusion:**

We identified older age, diabetes, history of MI, STEMI presentation, lower LVEF, and LMCA disease increased the risk of MACE/MACCE after PCI in older adults. Meanwhile, higher eGFR and successful PCI predicted lower adverse events risk. Future studies should focus on a more robust methodology and a precise definition of MACE.

**Registration:**

PROSPERO (CRD42023480332).

**Supplementary Information:**

The online version contains supplementary material available at 10.1186/s12877-024-04896-4.

## Introduction

Cardiovascular diseases, particularly coronary artery disease (CAD), are the most prevalent cause of mortality worldwide and represent a major health challenge [1–5]. With the improvements in the health care system and, thereby, the increase in life expectancy, the population of older people has become a noticeable component of society globally. This increase in the aging population means a dramatic incline in patients suffering from non-communicable diseases, and CAD is not an exception [6]. The burden of CAD on the older population necessitates more worldwide dedication to geriatric studies, especially in developing countries [7].

Atherosclerosis might progress more rapidly in older individuals and form more complex and calcified plaques associated with a higher risk of CAD [8]. Moreover, older people may not only be more prone to CAD, but their comorbidities also result in more complications and undesirable outcomes [9]. Making decisions about the appropriate therapeutic approach following CAD is a complex challenge for physicians, as older people and their families often prefer to choose a less invasive approach and conservative drug treatment. Although older age is a significant predictor of increased risk of major adverse cardiac events (MACE)/ major adverse cardiac and cerebrovascular events (MACCE) following percutaneous coronary intervention (PCI), other predictors, such as clinical or procedural characteristics, are also important [10].

Studies discussing the predictors of MACE/MACCE following PCI in older individuals are few, and the suggested predictors differ between these studies due to variations in population, sampling methods, and the definition of endpoints. Furthermore, no prior studies have examined these predictors systematically. Thus, The main objective of the present systematic review was to identify the main determinants of MACE/MACCE after PCI in the older population.

## Methods

The review protocol was registered at the International Prospective Register of Systematic Reviews (PROSPERO) with the identification code CRD42023480332. The present study followed the updated Preferred Reporting Items for Systematic Reviews and Meta-Analyses (PRISMA) statement for conducting systematic reviews and meta-analyses [11]..

### Eligibility criteria

This study included observational research that investigated the predictors of out-of-hospital outcomes (MACE or MACCE) in older adults (≥ 60 years old, according to the United Nations definition [12]) with coronary artery disease (acute or chronic) who underwent PCI. Studies were excluded according to the following criteria: (1) Conference abstracts, reviews, case reports/ series, and editorials; (2) The analyzed population consisted of other treatment approaches, e.g., coronary artery bypass grafting (CABG), thrombolytic, and medical treatment; (3) Comparison of outcomes between older adults and younger patients, with no separate report on older people; (4) No MACE/MACCE predictor identification by multivariable analysis; (5) Only one associated exposure with the outcome in the multivariable analysis; (6) No composite MACE/MACCE outcomes (including studies that defined only mortality as the endpoint); (7) In-hospital outcomes only; (8) Non-English articles. In the case of studies using the same database or with overlapping populations, the studies with a more complete recruitment period, overall number of PCI patients, follow-up, and measured outcomes were selected. Since the definition of MACE and MACCE varied markedly between the studies, no eligibility criterion was set based on the components of MACE/MACCE. Instead, we assessed the outcome definitions of the included studies for risk of bias.

### Information sources and search strategy

We searched PubMed, EMBASE, and Scopus from January 1st, 2000, to November 2nd, 2023, with no study design or language filters. Databases were searched using keywords like “elderly,” “Primary Percutaneous Intervention,” “Major Adverse Cardiovascular Events,” and “Major Adverse Cardiac-Cerebrovascular Events.” The detailed search syntax is provided in the Supplementary File.

### Selection process and data collection

Two independent groups (Group 1: A.S. and M.S.N., and Group 2: Z.K. and S.N.) screened the records for eligibility criteria in two stages (title/abstract and full text). Discrepancies were resolved by discussion with the review team. Two reviewers (A.H. and M.D.) reviewed the included articles and independently extracted the variables of interest. Disagreements were solved by consensus. Data items have been explained in detail in the Supplementary File.

### Risk of bias assessment

We utilized the Quality In Prognosis Studies (QUIPS) tool to evaluate the quality and risk of bias among the included studies [13]. QUIPS tool consists of six major components: (1) study participation (7 items), (2) study attrition (5 items), (3) prognostic factor measurement (6 items), (4) outcome measurement (3 items), (5) study confounding (7 items), and (6) statistical analysis and reporting (4 items). Two independent authors (M.S.N and S.N.) performed the assessment. The reviewers rated each component as low, moderate, and high risk of bias. The findings were compared, and any disagreement was solved by discussion with A.J.

## Synthesis methods

The provided effect sizes (i.e., hazard ratio (HR) or odds ratio (OR)) of each predictor from the multivariate analysis were extracted. Summary tables were then used to report the results qualitatively. Using a random-effects model, we pooled the most commonly reported effect sizes (HRs) for the quantitative synthesis demonstrated in forest plots. The choice of the model was made due to suspected heterogeneity among the included studies. Meta-analysis was performed if at least five studies reported HRs as a predictor. More details about the synthesis method are available in the Supplementary File.

The I^2^ test was used for assessing statistical heterogeneity. For publication bias assessment, funnel plots and Egger’s test were utilized. All analyses were performed with R V.4.2.1 (R Foundation for Statistical Computing, Vienna, Austria) and RStudio (RStudio, Boston, Massachusetts, USA), using “meta,” “metafor,” and “dmetar” packages [14].

## Results

**Fig. 1:**
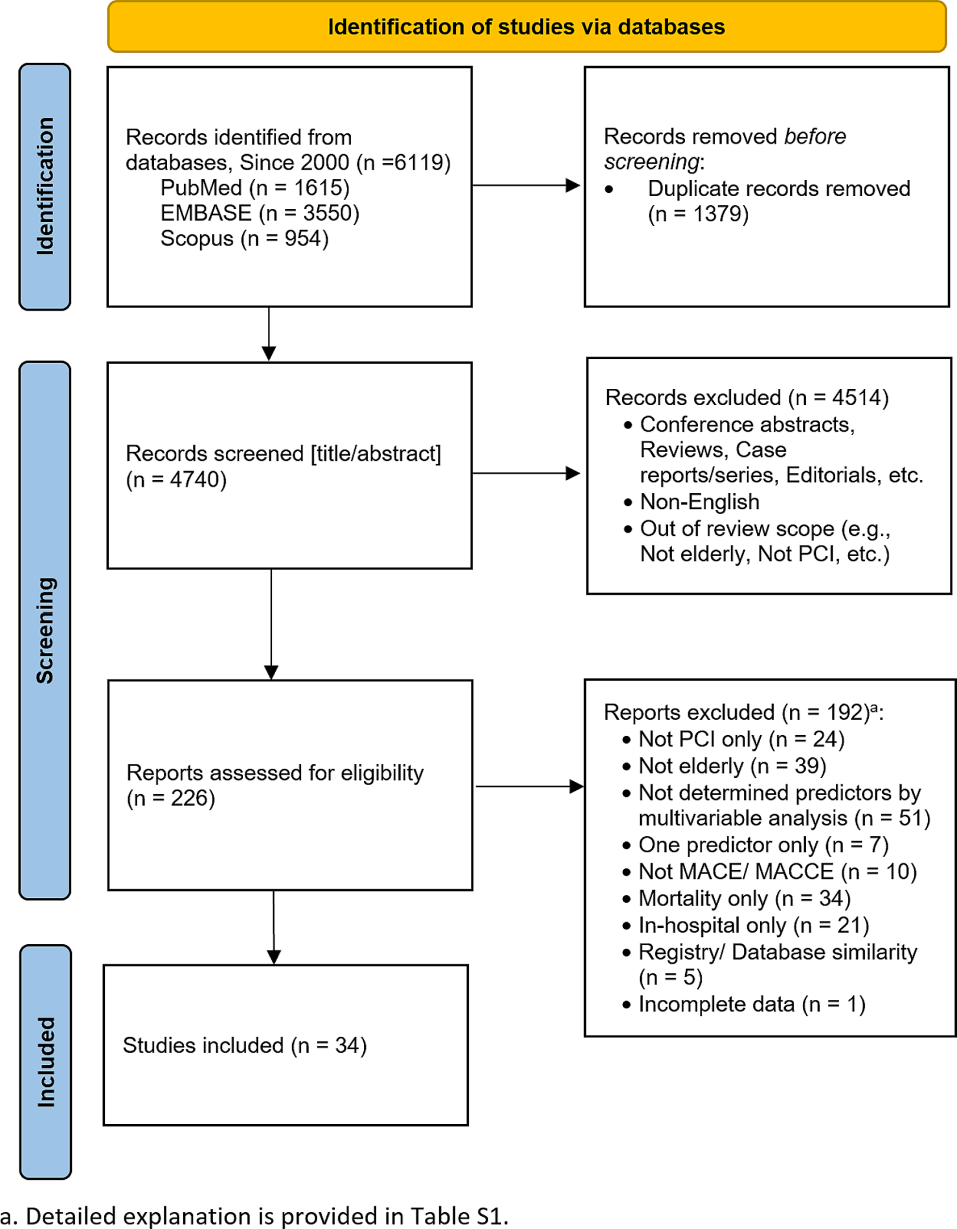
PRISMA flow diagram for study selection

We reviewed the full texts of 226 studies from 6119 identified records. Finally, thirty-four studies of 25,550 individuals and 11 multicenter investigations were selected [15–48]. The PRISMA flowchart of the study selection process is shown in Fig. 1. The reasons for excluding the remaining studies can be found in the Supplementary File. The baseline characteristics of the eligible studies are summarized in Table 1. Eleven (31.4%) were multicenter studies, and the median follow-up time ranged from one to 120 months.

**Table 1:** The baseline characteristics of eligible studies

First Author, Year (reference)	Country	Center	Inclusion year(s)	Elderly definition (year-old), [special population]	Overall N (MACE/MACCE rate, %)	Male (%), Mean age (years)	CAD (%)	Primary/ Emergent PCI (%)	MACE/MACCE components	Median/ Reported follow-up (months)
Cheng, 2023 [15]	China	SC	2009–2011	≥ 60	791 (17.32)	NR	NR	NR	ACM, MI, Revasc, Stroke	34
Li, 2023 [16]	China	SC	2019–2023	≥ 65	1286 (8.8)	61.35, 73.5	NSTE-ACS: 47.2 STEMI: 41.7	41.7	ACM, CM, MI, Stroke, TVR	1
Marschall, 2023 [17]	Spain	MC	2012–2019	≥ 75	2725 (9.9)	65.65, 80.97	ACS: 65	NR	CM, MI, Stroke, Revasc	12
Park, 2023 [18]	South Korea	MC	2017–2021	≥ 75	650 (7.8)	56.3, 80.5	NSTE-ACS: 54.8 SA: 45.2	None	ACM, MI, TVR, Stroke, Stent Thrombosis	12
Shimono, 2023 [19]	Japan	SC	2017–2020	≥ 65	239 (19.2)	73.6, 74.87	Stable CAD: 100	None	ACM, MI, Stroke, HFRH	32.1
Yan, 2023 [20]	China	SC	2013	≥ 65	2131 (12)	63.2, 70.3	ACS: 60.6	NR	ACM, MI, Stroke	120
Fallahzadeh, 2022 [21]	Iran	SC	2015–2019	≥ 80	610 (20.3)	65.7, 84	NSTE-ACS: 47.5 STEMI: 52.5	NR	ACM, ACS, Stroke/ TIA, Revasc	12
Horikoshi, 2022 [22]	Japan	MC	2008–2018	≥ 75	932 (18.9)	67.5, 81	ACS: 58	NR	ACM, MI	25
Lang, 2022 [23]	China	SC	2014–2019	≥ 65	617 (33.9)	60.3, 73	STEMI: 100	100	ACM, MI, Stroke, Revasc	56
Marino, 2022 [24]	Italy	SC	2009–2020	≥ 85	166 (41)	43.4, 87.8	NSTE-ACS: 60.8 STEMI: 39.2	NR	CM, MI, Revasc, Hosp.	18.5
Otowa, 2022 [25]	Japan	MC	2017	≥ 90	872 (8.1)	46.4, 92	NSTE-ACS: 25.8 STEMI: 40.7 SA: 17	NR	CM, MI, Stroke	12
Wang, 2022 [26]	China	SC	2009–2010	≥ 65	437 (16.2)	80.3, 72	STEMI: 100	100	ACM, MI, Revasc	59
Wang, 2022 [27]	China	SC	2013–2020	≥ 80	604 (19.5)	53.1, 82	NSTE-ACS: 67.4 STEMI: 32.6	20.5	CM, MI, Stroke, HFRH	48
Lattuca, 2021 [28]	France	MC	2012–2015	≥ 75 [BARC 2, 3, or 5]	181 (16.6)	56.4, 81.6	NSTE-ACS: 68 STEMI: 32	32.6	CM, MI, Stroke	12
Lim, 2021 [29]	Australia	MC	2013–2017	≥ 80	1875 (8)	59.3, 84.2	NSTE-ACS: 100	NR	ACM, MI, Stroke, Major bleeding, TVR/TLR, in-hospital cardiogenic shock or stent thrombosis, and a new requirement for dialysis.	1
Kalyoncuoğlu, 2021 [30]	Turkey	SC	2017–2019	≥ 60	253 (19)	71.5, 68.5	NSTEMI: 100	NR	ACM, MI, Stroke, Revasc	12
Kanwar, 2021 [31]	USA	MC	2005–2008	≥ 65	629 (NR)	69, 74.8	NR	NR	ACM, MI	35
Maruyama, 2021 [32]	Japan	MC	2012–2013	≥ 75	597 (10.2)	65.7, 80.9	ACS: 40.9 SA: 59.1	NR	ACM, MI, Stroke	51.6
Morici, 2020 [33]	Italy	MC	2012–2017	≥ 75	630 (10.8)	62.9, 80.2	ACS: 100	NR	ACM, MI, Stroke	12
Zhang, 2020 [34]	China	SC	2015–2019	≥ 70, [T2DM]	273 (17.2)	44.3, 78.4	ACS: 100	NR	CM, MI, Revasc	12
Berezhnoi, 2019 [35]	Russia	SC	2014–2017	≥ 80 [MVD]	305 (21.6)	34.4, 84.2	NSTE-ACS: 74.4 STEMI: 25.6	NR	ACM, MI, Stroke	12
Huang, 2019 [36]	China	SC	2015–2017	≥ 65	711 (NR)	66.9	NSTEMI: 43 STEMI: 57	NR	Cardiovascular/ cerebrovascular accident	24.6
Aghajani, 2018 [37]	Iran	SC	2004–2013	≥ 65	2772 (14.1)	62.3, 70.8	NSTE-ACS: 50 STEMI: 24.1	None	CM, MI, CABG Revasc, UA hosp., TVR/TLR	60
de la Torre Hernandez, 2018 [38]	Spain	MC	2006–2013	≥ 75, [MVD]	1830 (NR)	62.1, 81.1	STEMI: 100	100	CM, MI	24
De Rosa, 2018 [39]	Italy	MC	NR	≥ 75	311 (3.9)	66.6, 81.5	NSTE-ACS: 100	NR	CM, MI, Stent Thrombosis	12
Gerber, 2017 [40]	UK	SC	2006–2011	≥ 75	580 (14.1)	57.4, 79.8	ACS: 58.3 SA: 41.7	16.2	CM, MI, Stroke, TVR, TLR	30.8
Wei, 2016 [41]	China	SC	2012–2013	≥ 60 [LMCA]	64 (17.2)	75, 73.9	STEMI: 9.4	NR	CM, MI, Angina, Stroke, Worsening of HF, TVR	15.2
Yu, 2016 [42]	China	SC	2008–2012	≥ 60	1090 (8)	62.4, 68.9	NSTE-ACS: 100	NR	CM, MI	36
Uthamalingam, 2015 [43]	USA	SC	2000–2008	≥ 80	320 (5)	50.9, 83.6	ACS: 76.6 SA: 16.25	NR	CM, MI, TVR	12
Liu, 2013 [44]	Japan	SC	2005–2009	≥ 65 [CTO]	153 (18.3)	60.8, 76	NR	NR	CM, MI, TLR	36
Chen, 2012 [45]	China	SC	2005–2010	≥ 75 [multi-lesion]	502 (15.3)	63.5, 78.5	NSTE-ACS: 79.1 STEMI: 17.5	NR	CM, MI, Stroke, TLR/TVR	35.7
López-Palop, 2009 [46]	Spain	SC	2002–2006	≥ 80	176 (32.4)	60.8, 82.8	MI: 38.6 SA: 8.0	NR	ACM, MI, Revasc	26.3
Ma, 2008 [47]	China	SC	2004–2006	≥ 85	80 (16.25)	53.75, 87.5	NSTE-ACS: 81.25 STEMI: 18.75	18.75	CM, MI, TLR, TVR	36
Gach, 2003 [48]	Belgium	SC	1994–1999	≥ 80	158 (NR)	54.4, 85.2	UA: 49.4 MI: 5	76	ACM, MI, Revasc	24

**Abbreviations**:ACS: acute coronary syndrome, ACM: all-cause mortality, BARC: Bleeding Academic Research Consortium, CABG: coronary artery bypass graft surgery, CAD: coronary artery disease, Hosp: cardiac-related hospitalization, CM: cardiovascular mortality, CTO: chronic total occlusion, HFRH: HF requiring hospitalization, LMCA: left main coronary artery lesion, MACCE: major adverse cardiac cerebrovascular events, MACE: major adverse cardiovascular events, MC: multicenter, MI: myocardial infarction, MVD: multivessel disease, NR: not reported, NSTE-ACS: non-ST segment elevation ACS, PCI: percutaneous coronary intervention, Revasc: revascularization, SC: single center, STEMI: ST-segment elevation myocardial infarction, TLR: target lesion revascularization, TVR: target vessel revascularization, UA: unstable angina,

### Risk of bias assessment

**Fig. 2:**
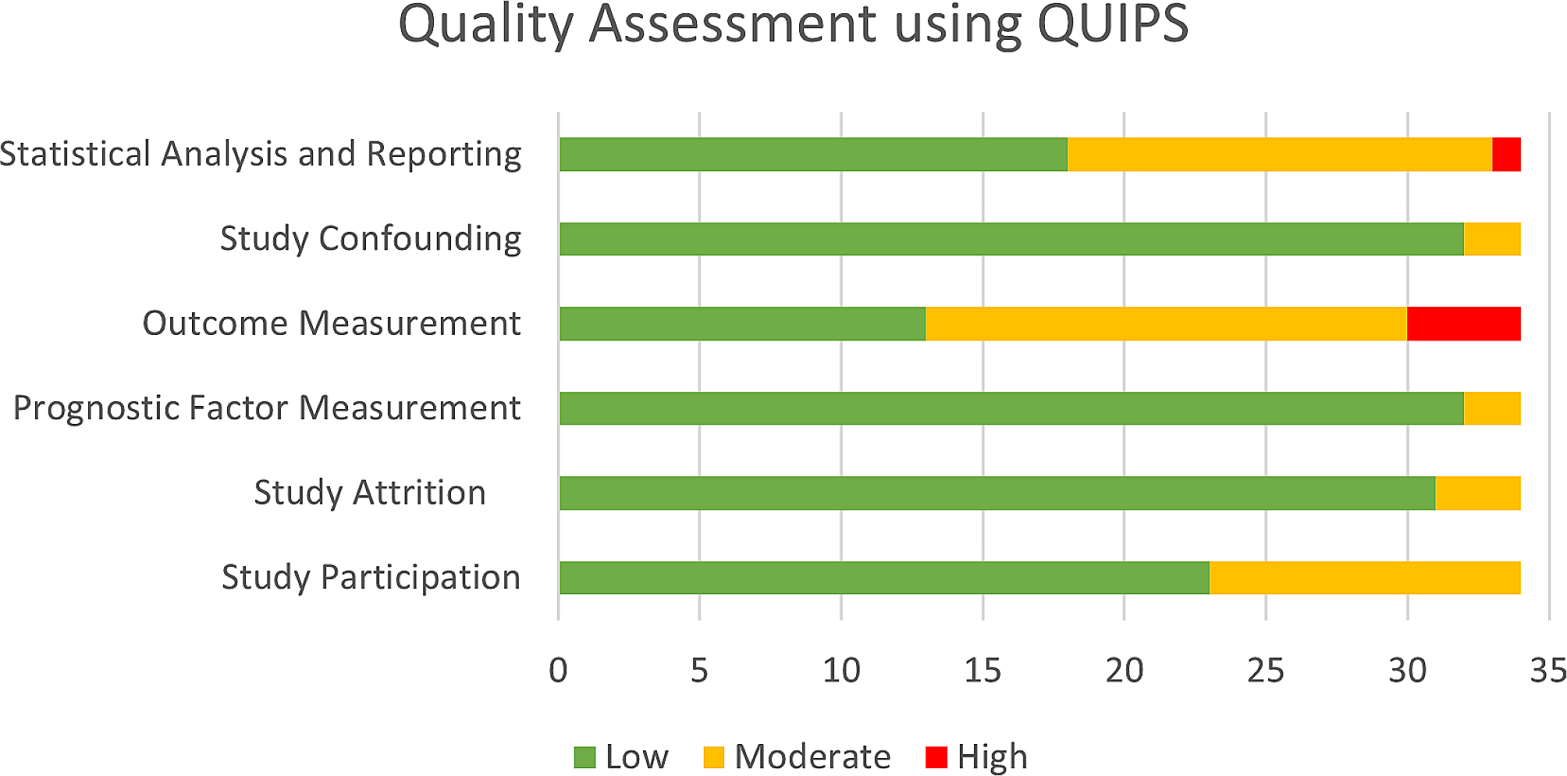
Summary of risk of bias assessment of the included studies using QUIPS

The risk of bias in study attrition, prognostic factor measurement, and study confounding domains was generally low (low-risk: 91.2%, 94.1%, and 94.1%, respectively). The risk of bias was higher in study participation and statistical analysis (moderate risk: 32.4% and 47.1%, respectively) domains. In the outcome measurement domain, 13 studies (38.2%) had low risk, four (11.8%) had high risk, and 17 (50%) had moderate risk of bias. The risk of bias among the included studies has been summarized in Fig. 2. A detailed version of the risk of bias assessment for each study is presented in Supplementary Table S1.

### Qualitative synthesis

The most common independent demographic predictors of increased risk of MACE/MACCE were higher age [15, 16, 22, 27, 28, 31–33, 36, 40, 42, 45] and male sex [25, 37]. The main comorbidities which predicted escalated MACE/MACCE risk were as follows: history of myocardial infarction (MI) before the studied exposure [15, 17, 24, 28, 33, 42, 45], CABG [20], Stroke [23], cardiovascular disease [42], diabetes (DM) [17, 26, 28, 30, 37, 39, 43, 44], hypertension (HTN) [20, 26, 45, 47], and chronic kidney disease (CKD) [29]. One study presented that patients with a positive CAD family history had a higher risk of adverse events [37]. Two studies identified higher frailty scores as a clinical predictor of higher MACE/MACCE risk [19, 31]. ST-elevation MI (STEMI) diagnosis in patients also resulted in a significantly higher occurrence of endpoints [27, 33, 35]. Lower left ventricular ejection fraction (LVEF) [17, 18, 20, 24, 29, 30, 32, 34–36, 38, 41, 46, 49], lower estimated glomerular filtration rate (eGFR) [17, 21, 22, 38, 41, 42], and anemia [17, 18, 42] were reported more frequently among the paraclinical predictors of higher risk of adverse events.

Several procedural variables, including multivessel disease (MVD) [16, 19, 26] and left main coronary artery (LMCA) involvement [17, 19, 21], were also identified as predictors of increased MACE/MACCE risk. PCI through radial access [17, 24, 35] and either successful PCI (thrombolysis in myocardial infarction or TIMI grade III) or complete revascularization [21, 38, 43, 48] resulted in a lower risk of adverse events. The summaries of significant predictors and adjusted variables in each study are provided in Supplementary Table S2. Effect sizes of exposures on MACE/MACCE are summarized in Supplementary Table S3.

### Quantitative synthesis

**Fig. 3:**
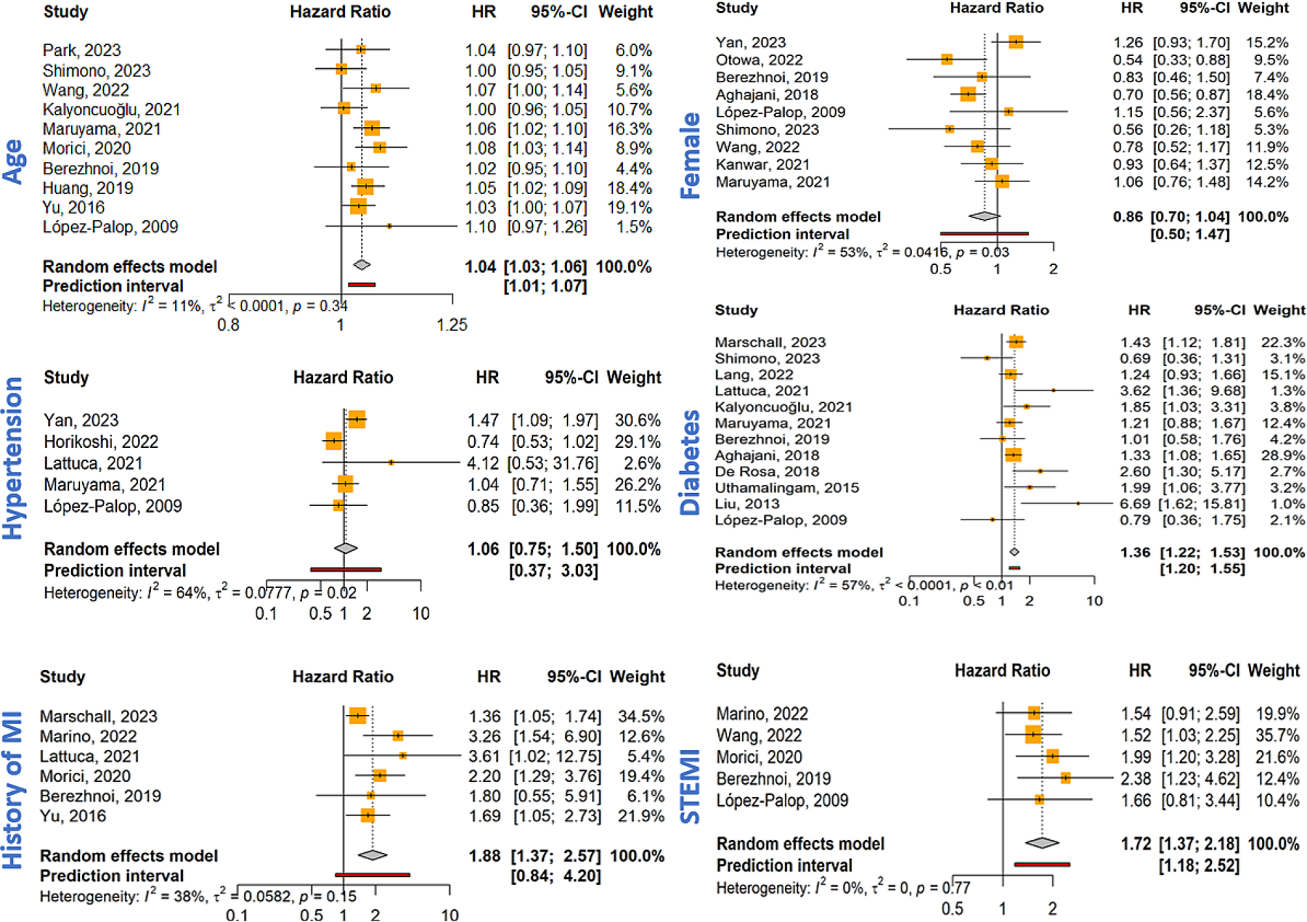
Forest plots of demographic and clinical predictors of MACE/MACCE. Abbreviations: MI: myocardial infarction, STEMI: ST elevation MI

**Fig. 4:**
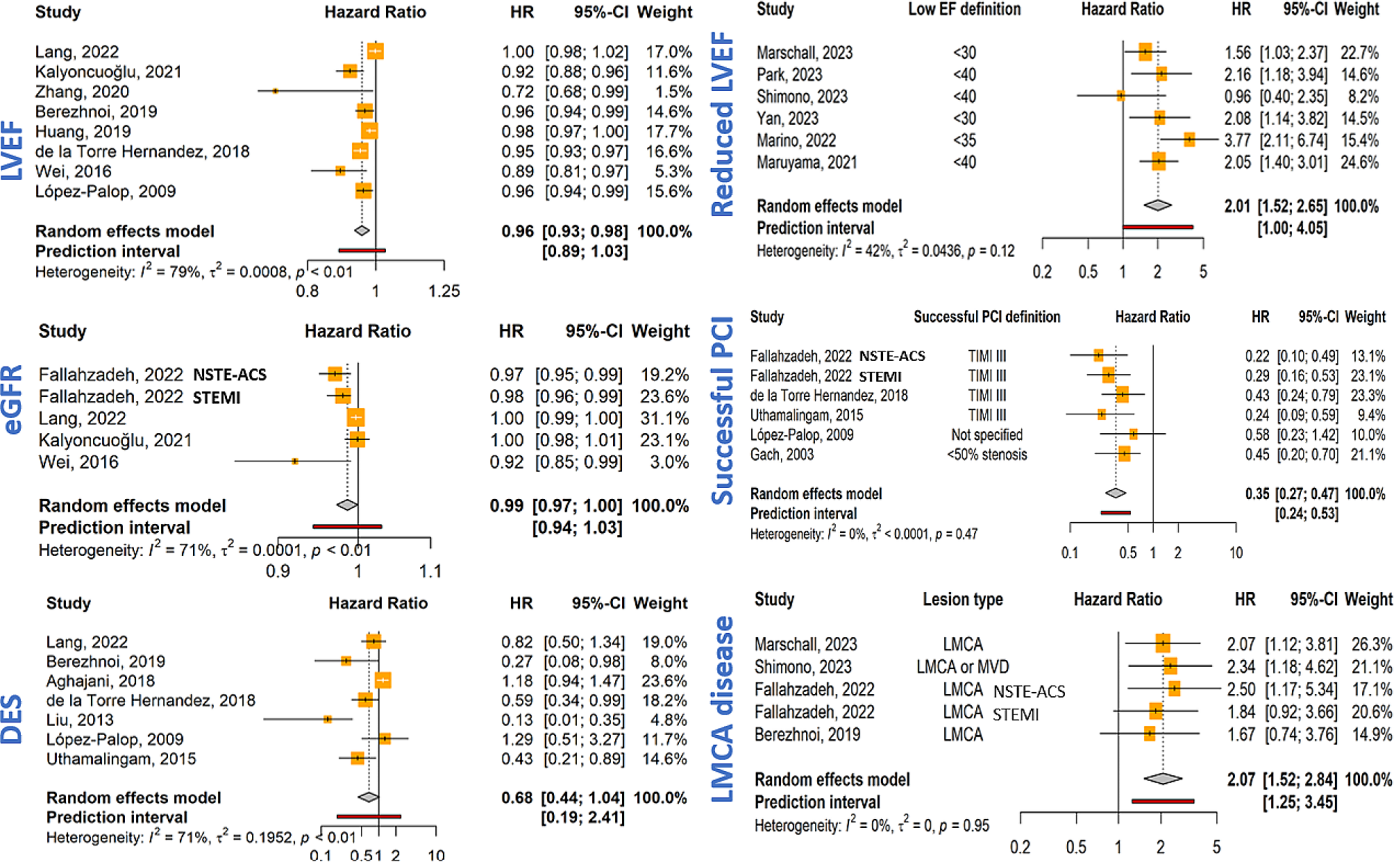
Forest plots of paraclinical and procedural predictors of MACE/MACCE. Abbreviations: LVEF: left ventricular ejection fraction, eGFR: estimated glomerular filtration rate, PCI: percutaneous coronary intervention, DES: drug-eluting stents, LMCA: left main coronary artery

The findings of 27 studies were eligible for meta-analysis. Increasing age was associated with higher MACE/MACCE risk (HR = 1.04, 95% CI: 1.03–1.06; *P*-value < 0.001, I^2^ = 11.3%). However, sex did not significantly predict increased MACE/MACCE risk (Female HR = 0.86, 95% CI: 0.70–1.04; *P*-value = 0.12, I^2^ = 52.6%, Fig. 3). Among the clinical exposures, DM (HR = 1.36, 95% CI: 1.22–1.53; *P*-value < 0.001, I^2^ = 56.7%), history of MI (HR = 1.88, 95% CI: 1.37–2.57; *P*-value < 0.001, I^2^ = 37.8%), and STEMI presentation (HR = 1.72, 95% CI: 1.37–2.18; *P*-value < 0.001, I^2^ = 0%) were significant determinants of MACE/MACCE increased occurrence (Fig. 3). Incremental LVEF prevented adverse events (HR = 0.96, 95% CI: 0.93–0.98; *P*-value < 0.001, I^2^ = 79.2%). On the other hand, reduced LVEF increased the risk of MACE/MACCE (HR = 2.01, 95% CI: 1.52–2.65; *P*-value < 0.001, I^2^ = 42.4%, Fig. 4). Higher kidney function, measured by eGFR, caused a slight decrease in the MACE/MACCE risk (HR = 0.99, 95% CI: 0.97-1.00; *P*-value = 0.04, I^2^ = 70.7%).

Drug-eluting stents (DES) were not associated with a statistically significant decrease in adverse events (HR = 0.68, 95% CI: 0.44–1.04; *P*-value = 0.08, I^2^ = 70.6%). LMCA disease was an important risk factor for MACE/MACCE (HR = 2.07, 95% CI: 1.52–1.2.84; *P*-value < 0.001, I^2^ = 0%), whereas procedural success (TIMI grade III) was protective against the endpoints (HR = 0.35, 95% CI: 0.27–0.47; *P*-value < 0.001, I^2^ = 0%). Other important predictors of a higher MACE risk after PCI were non-radial access [17, 24, 35], heart failure [15] and higher Killip class [23, 35, 36, 38], albumin [19], CONUT score [30], ACE inhibitors [15, 26], and GRACE score [34].

Funnel plots and Egger’s test measured publication bias. Except for the history of MI (Egger’s coefficient = 1.88, 95% CI: 0.63–3.14, *P*-value = 0.04), continuous LVEF (Egger’s coefficient=-3.38, 95% CI: -5.33,-1.42, *P*-value = 0.01), and DES (Egger’s coefficient=-2.45, 95% CI: -3.91,-0.98, *P*-value = 0.02), there was no significant publication bias among other predictors as described in Supplementary File. The funnel plots are presented in Supplementary Figure S1.

## Discussion

To our knowledge, the present study was the first systematic review and meta-analysis investigating the predictors of MACE/MACCE among older adults who had undergone PCI. According to the meta-analysis results, older age, diabetes, history of MI, STEMI, reduced LVEF, and LMCA disease were significant predictors of escalated risk of adverse events. Meanwhile, higher eGFR and successful PCI predicted lower MACE/MACCE risk. However, the pooled estimates for hypertension, female sex, and DES showed no significant associations with the increased risk of MACE.

Advances in PCI technology and techniques have resulted in better outcomes and fewer adverse events, especially in vulnerable older individuals [50]. The landmark FIRE trial indicated that older adults who underwent physiology-guided complete revascularization had a significantly lower risk of 1-year MACE than culprit–lesion–only PCI [51]. Conversely, Hanna et al. showed that older adults with stable ischemic heart disease who underwent complex PCI had a lower risk of target lesion revascularization but a higher risk of all-cause death compared to those who underwent noncomplex PCI [52]. It is crucial to note that while older adults may benefit more from PCI, they also face a higher risk of post-procedural complications and adverse events compared to younger patients [53, 54].

### Demographic and clinical predictors

Advanced age is a well-known risk factor for MACE/MACCE after PCI. Older adults often have more comorbidities, complex coronary lesions, and frailty that increase the procedural and post-procedural complications [55], which accounts for higher rates of MACCE [56]. Unlike age, the role of sex in the outcome of PCI is a topic of ongoing debate. Otawa et al. and Aghajani et al. were the only studies that found women to be a significant protective factor against one-year and five-year MACE, respectively [25, 37]. Contrariwise, in other studies not limited to older age, the female sex served as an independent predictor of a higher risk of one-year [57] and five-year [58] MACE after PCI. At the same time, it is essential to consider the interaction of age and sex, as studied by Alkhouli et al., in a large population of acute MI patients. They suggested that younger women generally have higher mortality compared to men, but older women have better outcomes compared to their male counterparts [59]. Similarly, Tonet et al. pointed out that in patients > 70 with acute coronary syndrome, the protective role of the female sex against higher mortality becomes evident when the model was adjusted for physical activity, and that female patients with preserved physical status had a better outcome compared to their male counterparts [60]. Therefore, identifying potential underappreciated confounding factors such as frailty, malnutrition [61], and physical activity and their interactions could clarify the complex role of age and sex in older adults on PCI outcomes. Alternatively, some studies proposed that the negative impact of female sex on PCI outcomes disappeared in older patients, and no significant difference was observed regarding the incidence of all-cause mortality and MACCE between men and women [62, 63]. The conflicting results in the literature could arise from population differences, assumed endpoints, and follow-up times. Therefore, the exact role of sex in the outcome of PCI among older adults remains controversial and requires more extensive investigations.

Hypertension is a common risk factor for CAD [64] and a leading cause of mortality in older people [65]. However, only Yan et al. could demonstrate that HTN is the predictor of increased MACE risk [20]. Notably, their adjusted model consisted of fewer variables than other studies, which may have affected their results. Diabetes has been consistently associated with an increased risk of MACE/MACCE after PCI [66–68], especially in patients with chronic total occlusions [69]. Our meta-analysis showed that diabetes predicts a higher risk of MACE/MACCE. Moreover, triglyceride glucose-body mass index, a predictor of type II DM [70], could also predict MACE/MACCE risk in older adults after PCI [15]. However, De Luca et al. concluded that the impact of diabetes on survival in advanced age (> 74 years old) becomes unclear when adjusted for baseline confounding factors, suggesting that diabetes is mainly responsible for significant comorbidity and more bleeding complications that result in higher mortality [71].

Our study indicated that the history of MI in older people is a predictive factor of major adverse events after PCI. Previous MI is responsible for decreased LVEF and heart failure; additionally, patients are more likely to develop complex CAD, which consequently results in higher mortality and MACE following PCI [72–74].

In the present study, older patients with STEMI presentation had higher MACE/MACCE risk than other presentations after PCI. Likewise, Wang et al., who investigated the interaction of STEMI, sex, and age and the risk of MACE, found that older women with STEMI had the highest risk of MACE [75]. On the other hand, Chang et al. found that STEMI could independently predict a higher revascularization incidence after the index event. In comparison, non-STEMI had a higher incidence of MACE [76]. Moreover, they indicated that older adults (> 65 years old) with non-STEMI had significantly longer hospital and ICU stays besides the need for mechanical circulatory support.

### Paraclinical and procedural predictors

The literature agrees that lower LVEF is associated with worse outcomes in older patients [77]. Similarly, decreased eGFR is associated with a higher risk of MACE in young and older adults undergoing PCI [78]. Consensus also exists for successful PCI as a protective factor against major adverse events [79–81].

Older patients receiving DES (> 75 years old) had remarkably lower risk of MACE and mortality compared to bare metal stents [82, 83]. Although our meta-analysis did not establish DES as a statistically significant protective factor against MACE/MACCE in older patients, the confidence interval was borderline (HR = 0.68, 95% CI: 0.44–1.04), suggesting that the association between DES and reduced risk of MACE/MACCE may be clinically meaningful. The advantages of DES over plain old balloon angioplasty [84] and bare metal stent [85] make DES clinically essential.

LMCA disease needs special attention due to its large amount of at-risk myocardium, and patients having MI with LCMA involvement are at significantly higher risks of cardiovascular morbidity and mortality compared to other obstructive CAD [86]. Although coronary artery bypass graft has long been the preferred treatment for LMCA with less long-term mortality and MACE/MACCE [87–90], PCI is considered in older people with higher surgical risk and frailty [91]. Our meta-analysis showed that LMCA disease in older patients predicted an increased risk of MACE/MACCE after PCI.

### Limitations

The current investigation has a few limitations. First, we considered studies that reported multiple predictors of MACE/MACCE in their multivariate analysis rather than a single exposure. Second, meta-analyses were performed using HRs resulting from a multivariate model. Some studies did not use multivariate analysis or report effect sizes for statistically non-significant variables. Some studies misreported the OR instead of the HR obtained from the time-to-event model. The abovementioned shortcomings may result in publication bias in the current systematic review. Regardless, the funnel plots showed little asymmetries. Third, the included studies exhibited significant heterogeneity mainly due to differences in endpoint definitions (MACE/MACCE) and population (age cut-offs, primary or elective PCI, and CAD type). Nevertheless, it was the first systematic review with a holistic investigation of MACE/MACCE predictors in older adults after PCI.

## Conclusion

We found that factors such as older age, diabetes, history of MI, STEMI presentation, lower LVEF, and LMCA disease increased the risk of MACE/MACCE after PCI in older adults. On the other hand, a higher eGFR and successful PCI were associated with a lower risk of adverse events. By identifying these predictors, healthcare providers can better assess their patients’ risk profiles and tailor interventions to mitigate adverse outcomes. Our risk of bias assessment revealed the need for more accurate study designs and statistical analysis, along with a uniform definition of MACE/MACCE.

### Electronic supplementary material

Below is the link to the electronic supplementary material.

Supplementary Material 1

Supplementary Material 2

Supplementary Material 3

## Data Availability

The datasets used during the current study are available from the corresponding author upon reasonable request.
