# Supplementary material for: Predictors of major adverse cardiac and cerebrovascular events after percutaneous coronary intervention in older adults: a systematic review and meta-analysis
Source: BMC Geriatr. 2024 Apr 12;24:337. doi: 10.1186/s12877-024-04896-4 (PMC11015672; doi:10.1186/s12877-024-04896-4)
Supplement: Supplementary file 1 — Supplementary Material 1 [file 12877_2024_4896_MOESM1_ESM.docx]

| **Table S1:** Quality assessment by QUIPS tool for each study | | | | | | | | |
| --- | --- | --- | --- | --- | --- | --- | --- | --- |
| ID | Author name | Study Date | Study Participation | Study Attrition | Prognostic Factor Measurement | Outcome Measurement | Study Confounding | Statistical Analysis and Reporting |
| 1 | Cheng | 2023 | Moderate | Low | Moderate | Moderate | Low | Moderate |
| 2 | Li | 2023 | Low | Low | Low | Moderate | Low | Moderate |
| 3 | Marschall | 2023 | Low | Low | Low | Low | Low | Low |
| 4 | Park | 2023 | Low | Low | Low | Moderate | Low | Low |
| 5 | Shimono | 2023 | Low | Low | Low | Moderate | Low | Low |
| 6 | Yan | 2023 | Moderate | Moderate | Low | Moderate | Low | Low |
| 7 | Fallazadeh | 2022 | Low | Low | Low | Moderate | Low | Moderate |
| 8 | Horikoshi | 2022 | Low | Moderate | Low | High | Low | Low |
| 9 | Lang | 2022 | Moderate | Low | Low | Moderate | Low | Low |
| 10 | Marino | 2022 | Moderate | Low | Low | Low | Low | Low |
| 11 | Otowa | 2022 | Low | Low | Low | Low | Low | Moderate |
| 12 | Wang | 2022 | Low | Low | Low | Moderate | Low | Moderate |
| 13 | Wang | 2022 | Low | Low | Low | Low | Low | Low |
| 14 | Lattuca | 2021 | Moderate | Low | Low | High | Low | Moderate |
| 15 | Lim | 2021 | Low | Low | Low | Moderate | Low | Moderate |
| 16 | Kalyoncuoğlu | 2021 | Low | Low | Low | Moderate | Low | Low |
| 17 | Kanwar | 2021 | Low | Low | Low | High | Moderate | Moderate |
| 18 | Maruyama | 2021 | Low | Low | Low | Moderate | Low | Low |
| 19 | Morici | 2020 | Low | Low | Low | Moderate | Low | Low |
| 20 | Zhang | 2020 | Low | Low | Low | Low | Low | Low |
| 21 | Berezhnoi | 2019 | Moderate | Low | Low | Moderate | Low | Moderate |
| 22 | Huang | 2019 | Low | Low | Low | High | Low | Low |
| 23 | Aghajani | 2018 | Low | Low | Low | Low | Low | Low |
| 24 | de la Torre Hernandez | 2018 | Moderate | Low | Low | Moderate | Low | Moderate |
| 25 | De Rosa | 2018 | Low | Low | Low | Low | Low | Low |
| 26 | Gerber | 2017 | Low | Low | Moderate | Low | Low | High |
| 27 | Wei | 2016 | Moderate | Moderate | Low | Low | Low | Moderate |
| 28 | Yu | 2016 | Moderate | Low | Low | Moderate | Low | Low |
| 29 | Uthamalingam | 2015 | Low | Low | Low | Low | Low | Low |
| 30 | Liu | 2013 | Moderate | Low | Low | Low | Low | Moderate |
| 31 | Chen | 2012 | Moderate | Low | Low | Low | Low | Moderate |
| 32 | Ma | 2008 | Low | Low | Low | Low | Low | Moderate |
| 33 | Gach | 2003 | Low | Low | Low | Moderate | Moderate | Moderate |
| 34 | Lopez | 2009 | Low | Low | Low | Moderate | Low | Low |

**Table S2.** Significant predictors and adjusted variables in each study

| Study | | Independent Predictors | | | | Included Variables |
| --- | --- | --- | --- | --- | --- | --- |
|  | | ***Demographic*** | ***Clinical*** | ***Paraclinical*** | ***Procedural*** |  |
| Cheng, 2023 [1] | | Age | HF, MI Hx | TyG-BMI index, Uric acid | ACEI | Age, HF, MI, TyG-BMI index, Uric acid, ACEI |
| Li, 2023 [2] | | Age | None | None | MVD | Age, Sex, BMI, HTN, DM, Cardiac surgery, SCAD/ NSTEMI/ STEMI presentation, CRUSADE score, MVD, Bivalirudin, Emergent PCI, Stenting, Stent diameter, Stent length |
| Marschall, 2023 [3] | | None | MI Hx, DM | LVEF, Hb, Creatinine clearance, Severe calcification | LMCA PCI, Access | MI, CKD, DM, ACS presentation, LVEF, Hb, Calcification, LMCA PCI, Non-radial access, Lesion length |
| Park, 2023 [4] | | None | None | Hb, LVEF | None | Age, BMI, CKD, Anemia, LVEF, High ischemic risk |
| Shimono, 2023 [5] | | None | Frailty | Albumin | LMCA/MVD | Frailty, Age, Sex, DM, LVEF, LMCA/MVD, Albumin |
| Yan, 2023 [6] | | Smoking | HTN, CABG Hx | LVEF | None | Smoking, HTN, CABG Hx, LVEF, Sex, BMI |
| Fallahzadeh, 2022 [7] | STEMI | None | None | eGFR | Post-PCI TIMI flow | eGFR, LMCA stenosis, post-PCI TIMI flow |
|  | NSTE-ACS | None | Dyslipidemia | eGFR | LMCA stenosis, post-PCI TIMI flow | eGFR, Dyslipidemia, LMCA stenosis, post-PCI TIMI flow |
| Horikoshi, 2022 [8] | | Age | NYHA class | eGFR | Statin | Age, HTN, DM, PAD, Stroke Hx, Smoking, eGFR, NYHA, Statin, ACS presentation, DES |
| Lang, 2022 [9] | | None | Stroke Hx | None | Staged PCI, Killip class | Staged PCI, DM, Stroke Hx, eGFR, HR, LVEF, Killip, Beta-blockers, IABP, Stenotic arteries N, Non-culprit diameter, DES |
| Marino, 2022 [10] | | None | MI Hx, Active malignancy | LVEF | RSS, Access | RSS, Active malignancy, MI Hx, Killip, LVEF, STEMI presentation, Access |
| Otowa, 2022 [11] | | Sex | Cardiogenic shock, Cardiac arrest | None | Prox.LAD PCI, Preprocedural PCI | Sex, PCI Hx, CKD, ACS presentation, Cardiogenic shock, Cardiac arrest, Prox.LAD lesion, Preprocedural OAC |
| Wang, 2022 [12] | | None | DM, HTN | None | TVD, Regular exercise, ACEI/ARB, Bleedings | Age, Sex, Systolic and diastolic BP, Heart rate, Glucose, LDL, HTN, DM, Stroke Hx, Smoking, TIMI flow, Drug (aspirin, ACEI/ARB, Beta blocker, Statin) adherence, Bleeding |
| Wang, 2022 [13] | | Age | STEMI | NT-pro-BNP | P12Y12 receptor inhibitors, Beta-blockers | Age, Sex, BMI, STEMI presentation, NT-pro-BNP, Creatinine, aspirin, Statin, P2Y12 receptor inhibitors, Beta-blockers |
| Lattuca, 2021^*^ [14] | | Age | DM, MI Hx | None | None | Age, Sex, DM, HTN, BMI, Smoking, Dyslipidemia, MI Hx, PCI Hx, CABG Hx, Malignancy Hx, HF, Chronic Respiratory Failure, Inflammatory disease, PAD, Creatinine clearance, Anemia, Presentation (STEMI/NSTEMI), Access, MVD, LVEF, Clopidogrel [vs Prasugrel], PPI |
| Lim, 2021 [15] | | None | CKD (eGFR≤30 or RRT) | LVEF | None | Age, eGFR, LVEF, GP IIb/IIIa inhibitors, DAPT (at discharge/follow-up), admission to PCI period, Mechanical ventricular support, Success, BMS only, Lesion location, AHA/ACC B2/C complexity |
| Kalyoncuoğlu, 2021 [16] | | None | DM | LVEF, CONUT score | None | Age, BMI, DM, eGFR, LVEF, CONUT score |
| Kanwar, 2021 [17] | | Age, QoL | Frailty | None | None | Age, Sex, [Frailty/ QoL] |
| Maruyama, 2021 [18] | | Age | None | TCBI, LVEF | Statin | Age, Sex, TCBI, ACS presentation, LVEF, Statin, CKD, PAD, DM, HTN |
| Morici, 2020 [19] | | Age | MI Hx, STEMI presentation | None | RSS | Age, Sex, MI Hx, Presentation, DM, PAD, COPD |
| Zhang, 2020 [20] | | None | None | Calprotectin, LVEF | GRACE | Age, BMI, HTN, Dyslipidemia, Insulin, ACEI/ARB, Statin, GRACE, LVEF, Calprotectin |
| Berezhnoi, 2019 [21] | | None | STEMI presentation, | LVEF | Killip III-IV, Radial access, DES | Age, Sex, DM, CKD, MI Hx, STEMI, LVEF, Killip class, Vessel/lesion location (LMA, LAD, LCx, RCA, Bifurcation), Calcification, SYNTAX, Access, DES, TIMI post-PCI |
| Huang, 2019 [22] | | Age | None | LVEF, SII | Killip class | Age, Sex, Smoking, HTN, CAD Hx, DM, Killip class, LVEF, CTnI, WBC, Neu, Lym, Hb, Creatinine, SII |
| Aghajani, 2018 [23] | | Sex | DM, CAD FHx | None | POBA | Sex, DM, CAD FHx, LAD territory, POBA, DES, |
| de la Torre Hernandez, 2018 ^a^ [24] | | None | None | LVEF, eGFR | Killip class, Delay, Bivalirudin, Thrombus aspiration, DES, TIMI flow post-PCI, DAPT, Complete Reavsc | CKD, LVEF, Killip class, Delay, Bivalirudin, Thrombus aspiration, DES, TIMI flow post-PCI, DAPT, Complete Reavsc |
| De Rosa, 2018 [25] | | None | DM | High platelet reactivity | None | DM, SYNTAX score, High platelet reactivity |
| Gerber, 2017 [26] | | Age | None | None | Bleeding | Age, Sex, ACS presentation, DM, HTN, Smoking, Bleeding |
| Wei, 2016 [27] | | None | None | LVEF, eGFR | None | Age, LVEF, eGFR, SYNTAX score, EuroScore |
| Yu, 2016 [28] | | Age | MI Hx, CVD Hx | Hb, eGFR | Staged PCI, Temporary pacemaker | Age, Sex, BMI, Heart rate, Atrial fibrillation, DM, HTN, Dyslipidemia, PAD, Smoking, MI/PCI/CVD Hx, Presentation, Hb, eGFR, LVEF, Early/ Delayed PCI, One-time/ Staged PCI, LMCA, Number of vessels, Temporary pacemaker, Complete Revasc, Contrast Volume |
| Uthamalingam, 2015 [29] | | None | DM | None | BMS, Type C lesion, Complete procedural success | Age, Sex, DM, HTN, Dyslipidemia, Smoking, CKD, MI Hx, CABG Hx, LVEF, PCI indication, GP IIa/IIIb, PCI complete success, LMCA |
| Liu, 2013 [30] | | None | DM | None | DES, Final MLD | DM, CKD, Lesion length, Stent length, Reference vessel diameter, Final MLD |
| Chen, 2012 [31] | | Age | MI Hx, HTN | None | None | Not specified clearly |
| López-Palop, 2009 [32] | | None | None | LVEF | Procedure success | Age, Sex, HTN, DM, STEMI presentation, PCI/CABG Hx, Stroke Hx, Diseased vessels number, LVEF, CKD, Malignancy, Complete Revasc, Procedure success, DES |
| Ma, 2008 [33] | | None | HTN | Creatinine | None | Creatinine, HTN, Presentation |
| Gach, 2003 [34] | | None | None | None | Complete Revasc, Stenting | Complete Revasc, Stenting, LVEF |
| a. The model with complete revascularization as an independent variable was chosen.  Abbreviations:  ACEI: angiotensin converting enzyme inhibitors, ARB: angiotensin receptor blockers, BNP: brain natriuretic peptide, BMI: body mass index, CKD: chronic kidney disease, CONUT: controlling nutritional status score, CRUSADE: Can Rapid Risk Stratification of Unstable Angina Patients Suppress Adverse Outcomes With Early Implementation of the ACC/AHA Guidelines, CVD: cardiovascular diseases, DAPT: dual antiplatelet therapy, DES: drug-eluting stent, , DM: diabetes mellitus, eGFR: estimated glomerular filtration rate, Hb: hemoglobin, HF: heart failure, HTN: hypertension, Hx: history, GRACE: Global Registry of Acute Coronary Events, LAD: left anterior descending artery, LCx: left circumflex artery, LDL: low density lipoprotein, LMCA: left main coronary artery, LVEF: left ventricular ejection fraction, MLD: minimal lumen diameter, NYHA: New York Heart Association, OAC: oral anticoagulant, PAD: peripheral artery/vascular disease, POBA: plain old balloon angioplasty, Prox: proximal, QoL: quality of life, RRT: renal replacement therapy, RSS: residual SYNTAX score, SCAD: spontaneous coronary artery dissection, SII: systemic immune-inflammatory index, SYNTAX: Synergy Between PCI With Taxus and Cardiac Surgery, TCBI: triglyceride-cholesterol-body weight index, TIMI: thrombolysis in myocardial infarction, TyG: triglyceride-glucose index; remaining abbreviations same as Table 1 | | | | | | |

| **Table S3**: Effect sizes of exposures on MACE/MACCE | | | | | | |
| --- | --- | --- | --- | --- | --- | --- |
| **Predictors** | | | **(Author, Year)** | **Effect Size** | | |
|  |  |  |  | Type | Estimate (95% CI) | P-value |
| Demographics | Age, years | | Cheng, 2023 [1] | OR | 1.07 (1.04-1.10) | <0.001 |
|  |  |  | Li, 2023 [2] | OR | 1.60 (1.06-2.41) [≥75] | 0.024 |
|  |  |  | Park, 2023 [4] | HR | 1.04 (0.97-1.10) | 0.278 |
|  |  |  | Shimono, 2023 [5] | HR | 1.00 (0.95-1.05) | 0.99 |
|  |  |  | Horikoshi, 2022 [8] | HR | 1.24 (1.07-1.44) [per 5 years] | 0.004 |
|  |  |  | Wang, 2022 [13] | HR | 1.071 (1.004-1.143) | 0.036 |
|  |  |  | Lattuca, 2021 [14] | HR | 8.10 (2.96-22.13) [>85] | <0.001 |
|  |  |  | Kalyoncuoğlu, 2021 [16] | HR | 1.005 (0.960-1.052) | 0.82 |
|  |  |  | Kanwar, 2021 [17] | HR | 1.17 (1.02-1.35) [per 5 years] | 0.023 |
|  |  |  | Maruyama, 2021 [18] | HR | 1.063 (1.025-1.101) | 0.001 |
|  |  |  | Morici, 2020 [19] | HR | 1.08 (1.03-1.14) | NR |
|  |  |  | Berezhnoi, 2019 [21] | HR | 1.020 (0.948-1.099) | 0.592 |
|  |  |  | Huang, 2019 [22] | HR | 1.05 (1.02-1.09) | <0.01 |
|  |  |  | Gerber, 2017 [26] | OR | 1.08 (1.01-1.15) | 0.02 |
|  |  |  | Yu, 2016 [28] | HR | 1.035 (1.002-1.069) | 0.038 |
|  |  |  | Chen, 2012 [31] | OR | 1.079 (1.007-1.157) | 0.032 |
|  |  |  | López-Palop, 2009 [32] | HR | 1.10 (0.97-1.26) | 0.140 |
|  | Sex | | Shimono, 2023 [5] | HR | 1.79 (0.85-3.79) [Male] | 0.12 |
|  |  |  | Yan, 2023 [6] | HR | 1.258 (0.930-1.702) [Female] | 0.136 |
|  |  |  | Otowa, 2022 [11] | HR | 0.54 (0.33-0.88) [Female] | 0.02 |
|  |  |  | Wang, 2022 [13] | HR | 1.275 (0.853-1.905) [Male] | 0236 |
|  |  |  | Kanwar, 2021 [17] | HR | 1.07 (0.73-1.56) [Male] | 0.73 |
|  |  |  | Maruyama, 2021 [18] | HR | 0.940 (0.676-1.308) [Male] | 0.714 |
|  |  |  | Berezhnoi, 2019 [21] | HR | 0.829 (0.458-1.501) [Female] | 0.536 |
|  |  |  | Aghajani, 2018 [23] | HR | 0.701 (0.565-0.870) [Female] | 0.001 |
|  |  |  | Gerber, 2017 [26] | OR | 1.02 (0.63-1.66) [Male] | 0.92 |
|  |  |  | López-Palop, 2009 [32] | HR | 1.15 (0.56-2.37) [Female] | 0.70 |
|  | BMI, kg/m^2^ | | Park, 2023 [4] | HR | 0.94 (0.87-1.02) | 0.166 |
|  |  |  | Yan, 2023 [6] | HR | 0.995 (0.957-1.035) | 0.807 |
|  |  |  | Wang, 2022 [13] | HR | 0.976 (0.920-1.035) | 0.417 |
|  |  |  | Kalyoncuoğlu, 2021 [16] | HR | 1.007 (0.897-1.131) | 0.91 |
|  | Smoking | | Yan, 2023 [6] | HR | 1.607 (1.203-2.146) | 0.001 |
|  |  |  | Gerber, 2017 [26] | OR | 1.18 (0.50-2.75) | 0.71 |
|  | Regular exercise | | Wang, 2022 [12] | OR | 0.472 (0.234-0.951) | 0.036 |
|  | Low QoL | | Kanwar, 2021 [17] | HR | 1.55 (1.09-2.22) | 0.016 |
| Clinical | Heart Rate, bpm | | Lang, 2022 [9] | HR | 1.007 (0.999-1.016) | 0.102 |
|  | Frailty | | Shimono, 2023 [5] | HR | 4.27 (1.86-9.80) [CFS ≥5] | <0.001 |
|  |  |  | Kanwar, 2021 [17] | HR | 2.54 (1.74-3.71) | <0.001 |
|  | Presentation | | Marschall, 2023 [3] | HR | 2.11 (0.96-1.63) [ACS] | 0.101 |
|  |  |  | Marino, 2022 [10] | HR | 1.54 (0.91-2.59) [STEMI] | 0.105 |
|  |  |  | Otowa, 2022 [11] | HR | 1.84 (0.91-3.72) [ACS] | 0.09 |
|  |  |  | Wang, 2022 [13] | HR | 1.522 (1.030-2.251) [STEMI] | 0.035 |
|  |  |  | Maruyama, 2021 [18] | HR | 1.184 (0.848-1.653) [ACS] | 0.321 |
|  |  |  | Morici, 2020 [19] | HR | 1.99 (1.20-3.28) [STEMI] | NR |
|  |  |  | Berezhnoi, 2019 [21] | HR | 2.381 (1.228-4.616) [STEMI] | 0.010 |
|  |  |  | Gerber, 2017 [26] | OR | 1.21 (0.72-2.02) [ACS] | 0.47 |
|  |  |  | López-Palop, 2009 [32] | HR | 1.66 (0.81-3.44) [STEMI] | 0.17 |
|  | Cardiogenic shock | | Otowa, 2022 [11] | HR | 2.32 (1.22-4.41) | 0.01 |
|  | Cardiac arrest | | Otowa, 2022 [11] | HR | 2.91 (1.28-6.62) | 0.01 |
|  | NYHA | | Horikoshi, 2022 [8] | HR | 5.24 (3.51-7.83) [Class IV] | <0.001 |
|  | Killip Class | | Lang, 2022 [9] | HR | 1.285 (1.049-1.574) | 0.015 |
|  |  |  | Marino, 2022 [10] | HR | 1.78 (0.97-3.26) [Class 3-4] | 0.062 |
|  |  |  | Berezhnoi, 2019 [21] | HR | 3.537 (1.726-7.251) [Class 3-4] | 0.001 |
|  |  |  | Huang, 2019 [22] | HR | 1.57 (1.29-1.90) | <0.01 |
|  |  |  | de la Torre Hernandez, 2018 [24] | HR | 0.2 (0.13-0.32) [Class 1-2] | <0.0001 |
|  | GRACE score | | Zhang, 2020 [20] | HR | 2.24 (1.03-4.65) | 0.01 |
|  | HTN | | Yan, 2023 [6] | HR | 1.466 (1.089-1.972) | 0.012 |
|  |  |  | Horikoshi, 2022 [8] | HR | 0.74 (0.53-1.02) | 0.07 |
|  |  |  | Wang, 2022 [12] | OR | 3.44 (1.43-8.30) | 0.006 |
|  |  |  | Lattuca, 2021 [14] | HR | 4.12 (0.53-31.76) | 0.174 |
|  |  |  | Maruyama, 2021 [18] | HR | 1.045 (0.707-1.546) | 0.824 |
|  |  |  | Gerber, 2017 [26] | OR | 1.11 (0.68-1.81) | 0.67 |
|  |  |  | Chen, 2012 [31] | OR | 1.653 (1.010-2.734) | 0.050 |
|  |  |  | López-Palop, 2009 [32] | HR | 0.85 (0.36-1.99) | 0.70 |
|  |  |  | Ma, 2008 [33] | OR | 3.201 (1.000-10.663) | 0.04 |
|  | DM | | Marschall, 2023 [3] | HR | 1.43 (1.12-1.81) | 0.014 |
|  |  |  | Shimono, 2023 [5] | HR | 0.69 (0.36-1.31) | 0.26 |
|  |  |  | Lang, 2022 [9] | HR | 1.243 (0.928-1.664) | 0.145 |
|  |  |  | Wang, 2022 [12] | OR | 2.63 (1.11-6.23) | 0.028 |
|  |  |  | Lattuca, 2021 [14] | HR | 3.62 (1.36-9.68) | 0.01 |
|  |  |  | Kalyoncuoğlu, 2021 [16] | HR | 1.852 (1.034-3.315) | 0.04 |
|  |  |  | Maruyama, 2021 [18] | HR | 1.211 (0.878-1.669) | 0.244 |
|  |  |  | Berezhnoi, 2019 [21] | HR | 1.015 (0.584-1.765) | 0.957 |
|  |  |  | Aghajani, 2018 [23] | HR | 1.333 (1.080-1.646) | 0.007 |
|  |  |  | De Rosa, 2018 [25] | HR | 2.597 (1.305-5.168) | 0.007 |
|  |  |  | Gerber, 2017 [26] | OR | 0.76 (0.38-1.53) | 0.44 |
|  |  |  | Uthamalingam, 2015 [29] | HR | 1.99 (1.06-3.77) | 0.03 |
|  |  |  | Liu, 2013 [30] | HR | 6.69 (1.62-15.81) | 0.01 |
|  |  |  | López-Palop, 2009 [32] | HR | 0.79 (0.36-1.75) | 0.56 |
|  | Dyslipidemia | | Fallahzadeh, 2022 [7] | HR | 0.37 (0.19-0.72) [in NSTE-ACS] | 0.003 |
|  | HF | | Cheng, 2023 [1] | OR | 1.81 (1.09-3.01) | 0.023 |
|  | MI/CAD Hx | | Cheng, 2023 [1] | OR | 1.98 (1.14-3.44) | 0.015 |
|  |  |  | Marschall, 2023 [3] | HR | 1.36 (1.05-1.74) | 0.016 |
|  |  |  | Marino, 2022 [10] | HR | 3.26 (1.54-6.90) | 0.002 |
|  |  |  | Lattuca, 2021 [14] | HR | 3.61 (1.02-12.75) | 0.046 |
|  |  |  | Morici, 2020 [19] | HR | 2.20 (1.29-3.76) | NR |
|  |  |  | Berezhnoi, 2019 [21] | HR | 1.805 (0.552-5.906) | 0.329 |
|  |  |  | Yu, 2016 [28] | HR | 1.691 (1.049-2.726) | 0.031 |
|  |  |  | Chen, 2012 [31] | OR | 1.440 (1.268-2.723) | 0.001 |
|  | CABG Hx | | Yan, 2023 [6] | HR | 1.818 (1.209-2.734) | 0.004 |
|  |  |  | Lattuca, 2021 [14] | HR | 2.13 (0.54-8.44) | 0.279 |
|  | PCI Hx | | Otowa, 2022 [11] | HR | 0.68 (0.34-1.34) | 0.27 |
|  |  |  | Lattuca, 2021 [14] | HR | 1.03 (0.33-3.25) | 0.956 |
|  | Revasc Hx | | López-Palop, 2009 [32] | HR | 2.27 (0.75-6.81) | 0.15 |
|  | CVD Hx | | Yu, 2016 [28] | HR | 1.875 (1.064-3.307) | 0.030 |
|  | PAD | | Maruyama, 2021 [18] | HR | 1.212 (0.804-1.828) |  |
|  | Stroke Hx | | Lang, 2022 [9] | HR | 1.531 (1.133-2.068) | 0.006 |
|  |  |  | López-Palop, 2009 [32] | HR | 0.73 (0.21-2.53) | 0.62 |
|  | CAD FHx | | Aghajani, 2018 [23] | HR | 1.489 (1.141-1.942) | 0.003 |
|  | CKD,  [Not defined on single laboratory finding] | | Otowa, 2022 [11] | HR | 2.23 (0.65-7.64) [Stage 4-5 vs stage 1-2] | 0.20 |
|  |  |  | Lim, 2021 [15] | OR | 2.10 (1.27-3.46) [GFR<30 or RRT] | <0.01 |
|  |  |  | Maruyama, 2021 [18] | HR | 1.247 (0.901-1.727) | 0.183 |
|  |  |  | Berezhnoi, 2019 [21] | HR | 1.295 (0.721-2.329) | 0.387 |
|  | Malignancy | | Marino, 2022 [10] | HR | 5.84 (2.38-14.33) [Dialysis] | <0.001 |
|  |  |  | López-Palop, 2009 [32] | HR | 1.12 (0.14-8.80) | 0.92 |
| Paraclinical | Serum Creatinine | | Wang, 2022 [13] | HR | 1.001 (0.995-1.006) | 0.846 |
|  |  |  | López-Palop, 2009 [32] | HR | 1.63 (0.65-4.1) [>2 mg/dL] | 0.30 |
|  |  |  | Ma, 2008 [33] | OR | 1.013 (1.006-1.020) | 0.004 |
|  | Hb, g/dL | | Marschall, 2023 [3] | HR | 1.53 (1.13-2.06) [<11] | 0.002 |
|  |  |  | Park, 2023 [4] | HR | 2.50 (1.41-4.45) [M:<13, F:<12] | 0.001 |
|  |  |  | Yu, 2016 [28] | HR | 0.980 (0.967-0.993) | 0.002 |
|  | Uric acid, µmol/L | | Cheng, 2023 [1] | OR | 1.04 (1.00-1.07) [per 20 unit] | 0.039 |
|  | TyG-BMI index | | Cheng, 2023 [1] | OR | 1.22 (1.01-1.47) [per one SD] | 0.038 |
|  | CONUT score | | Kalyoncuoğlu, 2021 [16] | HR | 1.434 (1.194-1.723) | <0.01 |
|  | Albumin, g/dL | | Shimono, 2023 [5] | HR | 3.63 (1.74-7.56) [<3.5] | <0.001 |
|  | Calcification | | Marschall, 2023 [3] | HR | 1.76 (1.27-2.50) [Severe] | 0.008 |
|  |  |  | Berezhnoi, 2019 [21] | HR | 0.953 (0.515-1.764) | 0.877 |
|  | LVEF, % | | Marschall, 2023 [3] | HR | 1.56 (1.03-2.37) [<30] | 0.006 |
|  |  |  | Park, 2023 [4] | HR | 2.16 (1.18-3.94) [<40] | 0.012 |
|  |  |  | Shimono, 2023 [5] | HR | 0.96 (0.40-2.35) [<40] | 0.94 |
|  |  |  | Yan, 2023 [6] | HR | 2.083 (1.136-3.817) [<30] | 0.018 |
|  |  |  | Lang, 2022 [9] | HR | 0.998 (0.979-1.017) | 0.833 |
|  |  |  | Marino, 2022 [10] | HR | 3.77 (2.11-6.74) [<35] | <0.001 |
|  |  |  | Lim, 2021 [15] | OR | 2.55 (1.35-4.84) [<35] | <0.01 |
|  |  |  | Kalyoncuoğlu, 2021 [16] | HR | 0.919 (0.879-0.961) | <0.01 |
|  |  |  | Maruyama, 2021 [18] | HR | 2.050 (1.396-3.010) [<40] | <0.001 |
|  |  |  | Zhang, 2020 [20] | HR | 0.72 (0.68-0.99) | 0.03 |
|  |  |  | Berezhnoi, 2019 [21] | HR | 0.965 (0.936-0.995) | 0.023 |
|  |  |  | Huang, 2019 [22] | HR | 0.98 (0.97-1.00) | 0.02 |
|  |  |  | de la Torre Hernandez, 2018 [24] | HR | 0.95 (0.93-0.97) | <0.0001 |
|  |  |  | Wei, 2016 [27] | HR | 0.89 (0.81-0.97) | 0.006 |
|  |  |  | López-Palop, 2009 [32] | HR | 0.96 (0.94-0.99) | 0.003 |
|  | NT-pro-BNP | | Wang, 2022 [13] | HR | 1.708 (1.215-2.400) [log scale] | 0.002 |
|  | Serum calprotectin | | Zhang, 2020 [20] | HR | 2.23 (1.26-7.62) | <0.01 |
|  | SII | | Huang, 2019 [22] | HR | 1.72 (1.23-2.40) | <0.01 |
|  | High platelet reactivity,  pre-discharge | | De Rosa, 2018 [25] | HR | 3.191 (1.373-7.417) | 0.007 |
|  | Kidney function (eGFR/ CrCl) | | Marschall, 2023 [3] | HR | 1.28 (1.04-1.63) [CrCl <60] | 0.011 |
|  |  |  | Park, 2023 [4] | HR | 1.55 (0.78-3.10) [eGFR<60] | 0.212 |
|  |  |  | Horikoshi, 2022 [8] | HR | 0.80 (0.67-0.94) [per eGFR SD] | 0.007 |
|  |  |  | Lang, 2022 [9] | HR | 0.997 (0.991-1.004) [eGFR] | 0.397 |
|  |  |  | Fallahzadeh, 2022 [7] | HR | 0.98 (0.96-0.99) [in STEMI] | 0.029 |
|  |  |  |  |  | 0.97 (0.95-0.99) [in NSTE-ACS] | 0.006 |
|  |  |  | Kalyoncuoğlu, 2021 [16] | HR | 0.999 (0.983-1.015) [eGFR] | 0.91 |
|  |  |  | de la Torre Hernandez, 2018 [24] | HR | 0.39 (0.24-0.61) [eGFR>60] | <0.0001 |
|  |  |  | Wei, 2016 [27] | HR | 0.92 (0.85-0.99) [eGFR] | 0.04 |
|  |  |  | Yu, 2016 [28] | HR | 1.956 (1.197-3.194) (eGFR≤60) | 0.007 |
| Procedural | DES/BMS | | Lang, 2022 [9] | HR | 0.820 (0.502-1.341) [DES] | 0.429 |
|  |  |  | Berezhnoi, 2019 [21] | HR | 0.274 (0.077-0.979) [DES] | 0.046 |
|  |  |  | Aghajani, 2018 [23] | HR | 1.179 (0.944-1.474) [DES] | 0.147 |
|  |  |  | de la Torre Hernandez, 2018 [24] | HR | 0.59 (0.34-0.99) [DES] | 0.045 |
|  |  |  | Uthamalingam, 2015 [29] | HR | 2.33 (1.12-4.86) [BMS] | 0.02 |
|  |  |  | Liu, 2013 [30] | HR | 0.13 (0.01-0.35) [DES] | 0.002 |
|  |  |  | López-Palop, 2009 [32] | HR | 1.29 (0.51-3.27) [DES] | 0.60 |
|  | Stenting | | Gach, 2003 [34] | HR | 0.56 (0.30-0.82) | 0.024 |
|  | POBA | | Aghajani, 2018 [23] | HR | 1.81 (1.15-2.85) | 0.01 |
|  | Staged PCI | | Lang, 2022 [9] | HR | 0.579 (0.390-0.860) (vs. one-time/ culprit-only) | 0.007 |
|  |  |  | Yu, 2016 [28] | HR | 0.638 (0.408-0.998) (vs. one-time) | 0.049 |
|  | Thrombus aspiration | | de la Torre Hernandez, 2018 [24] | HR | 0.57 (0.38-0.85) | 0.0058 |
|  | SYNTAX | | Berezhnoi, 2019 [21] | HR | 1.012 (0.991-1.040) | 0.331 |
|  |  |  | De Rosa, 2018 [25] | HR | 1.626 (0.807-3.276) [>15] | 0.174 |
|  | rSS | | Marino, 2022 [10] | HR | 0.60 (0.37-0.98) [0-8] | 0.04 |
|  |  |  | Morici, 2020 [19] | HR | 2.47 (1.51-4.06) [>8] | NR |
|  | Post-PCI TIMI flow | | Fallahzadeh, 2022 [7] | HR | 0.29 (0.16-0.53) [in STEMI] [III] | <0.001 |
|  |  |  | Fallahzadeh, 2022 [7] | HR | 0.22 (0.10-0.49) [in NSTE-ACS] [III] | <0.001 |
|  |  |  | Berezhnoi, 2019 [21] | HR | 0.783 (0.574-1.069) [continuous] | 0.124 |
|  |  |  | de la Torre Hernandez, 2018 [24] | HR | 0.43 (0.24-0.79) [III] | 0.0061 |
|  |  |  | Uthamalingam, 2015 [29] | HR | 0.24 (0.09-0.59) [III] | 0.002 |
|  | Lesion location/ Diseased vessel(s) / Involvement | LMCA/ Multivessel | Shimono, 2023 [5] | HR | 2.34 (1.18-4.62) | 0.01 |
|  |  | Multivessel | Li, 2023 [2] | OR | 1.75 (1.13-2.71) | 0.013 |
|  |  |  | Wang, 2022 [12] | OR | 2.71 (1.29-5.69) [TVD] | 0.008 |
|  |  | LMCA | Marschall, 2023 [3] | HR | 2.07 (1.12-3.81) | 0.013 |
|  |  |  | Fallahzadeh, 2022 [7] | HR | 1.84 (0.92-3.66) [in STEMI] | 0.081 |
|  |  |  |  |  | 2.50 (1.17-5.34) [in NSTE-ACS] | 0.018 |
|  |  |  | Berezhnoi, 2019 [21] | HR | 1.667 (0.739-3.758) | 0.218 |
|  |  | LAD | Otowa, 2022 [11] | HR | 1.79 (1.05-3.06) [Prox LAD] | 0.03 |
|  |  |  | Berezhnoi, 2019 [21] | HR | 1.060 (0.450-2.499) | 0.894 |
|  |  |  | Aghajani, 2018 [23] | HR | 0.828 (0.674-1.017) | 0.071 |
|  |  | LCx | Berezhnoi, 2019 [21] | HR | 1.665 (0.840-3.301) | 0.144 |
|  |  | RCA | Berezhnoi, 2019 [21] | HR | 0.615 (0.311-1.215) | 0.161 |
|  |  | Bifurcation | Berezhnoi, 2019 [21] | HR | 0.726 (0.351-1.501 | 0.387 |
|  |  | Number of vessels | Lang, 2022 [9] | HR | 1.163 (0.856-1.581) | 0.333 |
|  |  |  | López-Palop, 2009 [32] | HR | 1.04 (0.65-1.66) | 0.87 |
|  | Non-culprit lesion diameter stenosis | | Lang, 2022 [9] | HR | 1.126 (0.927-1.368) | 0.231 |
|  | Final MLD | | Liu, 2013 [30] | HR | 0.37 (0.13-0.90) | 0.03 |
|  | Access | | Marschall, 2023 [3] | HR | 1.56 (1.19-2.05) [non-radial] | 0.002 |
|  |  |  | Marino, 2022 [10] | HR | 0.49 (0.29-0.83) [radial] | 0.009 |
|  |  |  | Berezhnoi, 2019 [21] | HR | 0.512 (0.284-0.922) | 0.026 |
|  | Lesion length | | Marschall, 2023 [3] | HR | 1.23 (0.92-1.64) | 0.096 |
|  |  |  | Liu, 2013 [30] | HR | 1.05 (0.98-1.12) | 0.15 |
|  | High ischemic risk | | Park, 2023 [4] | HR | 1.48 (0.66-3.32) | 0.337 |
|  | Type C lesion | | Uthamalingam, 2015 [29] | HR | 1.58 (1.39-1.88) | 0.01 |
|  | Successful procedure | | López-Palop, 2009 [32] | HR | 0.25 (0.08-0.76) | 0.02 |
|  | Time delay | | de la Torre Hernandez, 2018 [24] | HR | 0.66 (0.45-0.99) [<6 hours] | 0.041 |
|  | Complete Revasc | | de la Torre Hernandez, 2018 [24] | HR | 0.62 (0.42-0.98) [<50% stenosis] | 0.047 |
|  |  |  | López-Palop, 2009 [32] | HR | 0.58 (0.23-1.42) | 0.23 |
|  |  |  | Gach, 2003 [34] | HR | 0.45 (0.20-0.70) [<50% stenosis] | 0.002 |
|  | Temporary Pacemaker | | Yu, 2016 [28] | HR | 6.621 (2.008-21.827) | 0.002 |
|  | IABP | | Lang, 2022 [9] | HR | 1.109 (0.755-1.889) | 0.449 |
|  | DAPT | | de la Torre Hernandez, 2018 [24] | HR | 0.63 (0.34-0.95) [>6 months] | 0.029 |
|  | Preprocedural OAC | | Otowa, 2022 [11] | HR | 2.10 (1.07-4.12) | 0.03 |
|  | P2Y12 receptor inhibitors | | Wang, 2022 [13] | HR | 0.319 (0.181-0.561) | <0.001 |
|  | Bivalirudin | | de la Torre Hernandez, 2018 [24] | HR | 0.40 (0.18-0.86) | 0.019 |
|  | ASA | | Wang, 2022 [13] | HR | 1.044 (0.579-1.884) | 0.885 |
|  | Statin | | Horikoshi, 2022 [8] | HR | 0.55 (0.40-0.75) | <0.001 |
|  |  |  | Wang, 2022 [13] | HR | 0.601 (0.356-1.015) | 0.057 |
|  |  |  | Maruyama, 2021 [18] | HR | 0.463 (0.322-0.667) | <0.001 |
|  | ACEIs | | Cheng, 2023 [1] | OR | 1.94 (1.27-2.96) | 0.002 |
|  |  |  | Wang, 2022 [12] | OR | 0.298 (0.144-0.618) | 0.001 |
|  | Beta Blockers | | Lang, 2022 [9] | HR | 1.210 (0.830-1.764) | 0.323 |
|  |  |  | Wang, 2022 [13] | HR | 0.639 (0.429-0.950) | 0.027 |
|  | Bleedings | | Wang, 2022 [12] | OR | 12.670 (3.822-42.001) | <0.001 |
|  |  |  | Gerber, 2017 [26] | OR | 5.39 (2.68-10.85) | 0.0001 |

**References**

1. Cheng, Y., et al., *Association between triglyceride glucose-body mass index and cardiovascular outcomes in patients undergoing percutaneous coronary intervention: a retrospective study.* Cardiovasc Diabetol, 2023. **22**(1): p. 75.

2. Li, Q., et al., *Bivalirudin versus Heparin on Net Adverse Clinical Events, Major Adverse Cardiac and Cerebral Events, and Bleeding in Elderly Chinese Patients Treated with Percutaneous Coronary Intervention.* Tohoku J Exp Med, 2023.

3. Marschall, A., et al., *Outcomes Prediction in Complex High-Risk Indicated Percutaneous Coronary Interventions in the Older Patients.* Am J Cardiol, 2023. **205**: p. 465-472.

4. Park, J.I., et al., *Efficacy of Percutaneous Coronary Intervention With Synergy Stents in Patients Aged ≥75 Years: 1-Year Clinical Outcomes from the Synergy Elderly Registry.* Am J Cardiol, 2023. **204**: p. 43-52.

5. Shimono, H., et al., *Association of preoperative clinical frailty and clinical outcomes in elderly patients with stable coronary artery disease after percutaneous coronary intervention.* Heart Vessels, 2023. **38**(10): p. 1205-1217.

6. Yan, K., et al., *Five-Year Prognostic Value of DAPT Score in Older Patients undergoing Percutaneous Coronary Intervention: A Large-Sample Study in the Real World.* J Atheroscler Thromb, 2023. **30**(8): p. 1057-1069.

7. Fallahzadeh, A., et al., *Outcome of Percutaneous Coronary Intervention in Old Patients Presenting with Acute Coronary Syndrome.* Arch Iran Med, 2022. **25**(8): p. 523-532.

8. Horikoshi, T., et al., *A Propensity Score Matched Analysis of Statin Effects on Major Adverse Cardiac Events after Percutaneous Coronary Intervention in Patients Over 75 Years Old.* Intern Med, 2022. **61**(18): p. 2711-2719.

9. Lang, J., et al., *Staged revascularization vs. culprit-only percutaneous coronary intervention for multivessel disease in elderly patients with ST-segment elevation myocardial infarction.* Front Cardiovasc Med, 2022. **9**: p. 943323.

10. Marino, M., et al., *Complete Percutaneous Revascularization in Patients Aged ≥85 Years With Acute Coronary Syndrome and Multivessel Coronary Artery Disease.* Am J Cardiol, 2022. **180**: p. 10-16.

11. Otowa, K., et al., *One-year outcome after percutaneous coronary intervention in nonagenarians: Insights from the J-PCI OUTCOME registry.* Am Heart J, 2022. **246**: p. 105-116.

12. Wang, J., et al., *Impact of BMI on Long-Term Outcomes in Patients with ST-Segment Elevation Myocardial Infarction after Primary Percutaneous Coronary Intervention.* Int J Clin Pract, 2022. **2022**: p. 6210204.

13. Wang, J.L., et al., *Sex-related differences in clinical outcomes and predictive factors in the very elderly patients with ACS undergoing PCI.* Front Cardiovasc Med, 2022. **9**: p. 950165.

14. Lattuca, B., et al., *Bleeding in the Elderly: Risk Factors and Impact on Clinical Outcomes After an Acute Coronary Syndrome, a Sub-study of the Randomized ANTARCTIC Trial.* Am J Cardiovasc Drugs, 2021. **21**(6): p. 681-691.

15. Lim, M., et al., *Clinical Outcomes in Older Patients Undergoing Percutaneous Coronary Intervention for Non-ST-Elevation Acute Coronary Syndromes.* Heart Lung Circ, 2021. **30**(2): p. 275-281.

16. Kalyoncuoğlu, M., et al., *Predicting One-Year Deaths and Major Adverse Vascular Events with the Controlling Nutritional Status Score in Elderly Patients with Non-ST-Elevated Myocardial Infarction Undergoing Percutaneous Coronary Intervention.* J Clin Med, 2021. **10**(11).

17. Kanwar, A., et al., *Poor quality of life in patients with and without frailty: co-prevalence and prognostic implications in patients undergoing percutaneous coronary interventions and cardiac catheterization.* Eur Heart J Qual Care Clin Outcomes, 2021. **7**(6): p. 591-600.

18. Maruyama, S., et al., *Impact of nutritional index on long-term outcomes of elderly patients with coronary artery disease: sub-analysis of the SHINANO 5 year registry.* Heart Vessels, 2021. **36**(1): p. 7-13.

19. Morici, N., et al., *Residual SYNTAX Score and One-Year Outcome in Elderly Patients With Acute Coronary Syndrome.* CJC Open, 2020. **2**(4): p. 236-243.

20. Zhang, W., et al., *Prognostic value of serum calprotectin level in elderly diabetic patients with acute coronary syndrome undergoing percutaneous coronary intervention: A Cohort study.* Medicine (Baltimore), 2020. **99**(33): p. e20805.

21. Berezhnoi, K., L. Kokov, and A. Vanyukov, *Effects of complete revascularization on long-term treatment outcomes in patients with multivessel coronary artery disease over 80 years of age admitted for acute coronary syndrome.* Cardiovasc Diagn Ther, 2019. **9**(4): p. 301-309.

22. Huang, J., et al., *Systemic Immune-Inflammatory Index Predicts Clinical Outcomes for Elderly Patients with Acute Myocardial Infarction Receiving Percutaneous Coronary Intervention.* Med Sci Monit, 2019. **25**: p. 9690-9701.

23. Aghajani, H., et al., *Predictors of Long-term Major Adverse Cardiac Events Following Percutaneous Coronary Intervention in the Elderly.* Arch Iran Med, 2018. **21**(8): p. 344-348.

24. de La Torre Hernandez, J.M., et al., *Multivessel disease in patients over 75years old with ST elevated myocardial infarction. Current management strategies and related clinical outcomes in the ESTROFA MI+75 nation-wide registry.* Cardiovasc Revasc Med, 2018. **19**(5 Pt B): p. 580-588.

25. De Rosa, R., et al., *High on-treatment platelet reactivity and outcome in elderly with non ST-segment elevation acute coronary syndrome - Insight from the GEPRESS study.* Int J Cardiol, 2018. **259**: p. 20-25.

26. Gerber, R.T., et al., *Age is not a bar to PCI: Insights from the long-term outcomes from off-site PCI in a real-world setting.* J Interv Cardiol, 2017. **30**(4): p. 347-355.

27. Wei, Z., et al., *Comparison of Percutaneous Coronary Intervention Versus Coronary Artery Bypass Graft in Aged Patients With Unprotected Left Main Artery Lesions.* Int Heart J, 2016. **57**(6): p. 682-688.

28. Yu, X.F., et al., *Staged versus "one-time" multivessel intervention in elderly patients with non-ST-elevation acute coronary syndrome.* J Geriatr Cardiol, 2016. **13**(9): p. 760-767.

29. Uthamalingam, S., et al., *Long term outcomes in octogenarians undergoing percutaneous coronary intervention: comparison of bare metal versus drug eluting stent.* Int J Cardiol, 2015. **179**: p. 385-9.

30. Liu, W., et al., *Impact of diabetes on long term follow-up of elderly patients with chronic total occlusion post percutaneous coronary intervention.* J Geriatr Cardiol, 2013. **10**(1): p. 16-20.

31. Chen, J., et al., *Incomplete revascularization in the drug eluting stent era permits meaningful long-term (12-78 months) outcomes in patients ≥ 75 years with acute coronary syndrome.* J Geriatr Cardiol, 2012. **9**(4): p. 336-43.

32. López-Palop, R., et al., *Safety and efficacy of coronary drug-eluting stents in octogenarians.* Rev Esp Cardiol, 2009. **62**(11): p. 1250-9.

33. Ma, H.Y., et al., *Long-term outcome of patients of over 85 years old with acute coronary syndrome undergoing percutaneous coronary stenting: a comparison of bare metal stent and drug eluting stent.* Chin Med J (Engl), 2008. **121**(10): p. 887-91.

34. Gach, O., et al., *Predictors of early and late outcome of percutaneous coronary intervention in octogenarians.* Acta Cardiol, 2003. **58**(4): p. 289-94.
