# Supplementary material for: Predictors of major adverse cardiac and cerebrovascular events after percutaneous coronary intervention in older adults: a systematic review and meta-analysis
Source: BMC Geriatr. 2024 Apr 12;24:337. doi: 10.1186/s12877-024-04896-4 (PMC11015672; doi:10.1186/s12877-024-04896-4)
Supplement: Supplementary file 3 — Supplementary Material 3 [file 12877_2024_4896_MOESM3_ESM.docx]

**Supplementary File**

**Contents** Page number

1. **Supplementary Methods**

- Search strategy……………………………………………………………………...………….1
- Data items………………………………………………………………………..…………….2
- Synthesis methods………………………………………………………………….………….2

1. **Supplementary Results**

- Exclusion reasons………………………………………………………………..…………….4
- Exclusion table…………………………………………………………………..…………….5
- Publication bias……………………………………………………………………………….10
- Supplementary Figure……….……………………………...…………………..…………….10
- References………………………………………………………………………….………...11

**Supplementary Methods**

**Search strategy**

- **PubMed:**

("Percutaneous Coronary Intervention"[Title/Abstract] OR "balloon angioplasty"[Title/Abstract] OR "Coronary Angioplasty"[Title/Abstract] OR "Percutaneous Coronary revascularization"[Title/Abstract]) AND (MACE[Title/Abstract] OR “major adverse cardiac event”[Title/Abstract] OR “major cardiac event”[Title/Abstract] OR “outcome”[Title/Abstract] OR survival[Title/Abstract] OR prognosis[Title/Abstract]) AND (aged[Title/Abstract] OR elderly[Title/Abstract] OR "old age"[Title/Abstract] OR "older adult"[Title/Abstract] OR “older people” [Title/Abstract]) AND (Predictor[Title/Abstract] OR  Predictors[Title/Abstract] OR cohort[Title/Abstract] OR prospective[Title/Abstract])

- **Embase:**

((elderly OR ‘old age’ OR ‘older adult’ OR ‘Aged’ OR ‘older people’):ab,ti) AND ((‘Percutaneous Coronary Intervention’ OR ‘balloon angioplasty’ OR ‘Coronary Angioplasty’ OR ‘Percutaneous Coronary revascularization’):ab,ti) AND ((MACE OR ‘major adverse cardiac event’ OR ‘major cardiac event’ OR ‘outcome’ OR survival OR prognosis):ab,ti) AND ((Predictor OR  Predictors OR cohort OR prospective):ab,ti)

- **Scopus:**

(TITLE("Percutaneous Coronary Intervention") OR TITLE("balloon angioplasty") OR TITLE("Coronary Angioplasty") OR TITLE("Percutaneous Coronary revascularization") OR ABS("Percutaneous Coronary Intervention") OR ABS("balloon angioplasty") OR ABS("Coronary Angioplasty") OR ABS("Percutaneous Coronary revascularization")) AND (TITLE(MACE) OR TITLE( “major adverse cardiac event”) OR TITLE( “major cardiac event”) OR TITLE( “outcome”) OR TITLE(survival) OR TITLE(prognosis) OR ABS(MACE) OR ABS( “major adverse cardiac event”) OR ABS( “major cardiac event”) OR ABS( “outcome”) OR ABS(survival) OR ABS(prognosis)) AND (TITLE(aged) OR TITLE(elderly) OR TITLE("old age") OR TITLE("older adult") OR TITLE(“older people”) OR ABS(aged) OR ABS(elderly) OR ABS("old age") OR ABS("older adult") OR ABS(“older people”)) AND (TITLE(Predictor) OR  TITLE(Predictors) OR TITLE(cohort) OR TITLE(prospective) OR ABS(Predictor) OR ABS(Predictors) OR ABS(cohort) OR ABS(prospective))

**Data items**

We extracted study characteristics, including identification (i.e., first author, publication year, and country), setting (multicenter vs. single-center), and study period. Population characteristics were elderly definition cut-off, mean age, male gender percentage, indications of PCI or patient presentation (i.e., stable angina (SA), Non-ST segment elevation acute coronary syndrome (NSTE-ACS, including unstable angina (UA) or NSTE-myocardial infarction (NSTEMI)), and ST-segment elevation MI (STEMI)), PCI urgency (elective vs emergent primary PCI (pPCI)), inclusion criteria, and total patients. The outcome components, occurrence rate, and follow-up duration were extracted from the studies.

We classified predictors of MACE/MACCE into four categories: 1. Demographics, including age, sex, BMI, and habitual history; 2. Clinical: consisting of clinical presentation (ACS, STEMI, etc.) and comorbidities (such as CVD, diabetes (DM), chronic kidney disease (CKD), etc.); 3. Paraclinical: laboratory and imaging (including echocardiography) findings and derived novel indices; and 4. Procedural: coronary angiography (CAG) findings, PCI interventions (i.e., type and number of stents, etc.), and medications.

**Synthesis methods**

If a predictor was adjusted in the model and the effect size was not reported, we considered it a missing value. Independent predictors of the target endpoint and the adjusted variables were reported in the pre-specified datasheet. For binary exposures, in which two different reference groups were reported in the selected studies, the effect sizes and its 95% confidence interval were inversed to estimate the effect size of the target group.

**Supplementary Results**

**Exclusion reasons:**

- Wrong Population:

1) Not PCI / Not PCI only (i.e., CABG, medical treatment, or thrombolytic)

2) Not older adults/ Included younger population in the analysis

- Wrong Exposure:

3) Not multivariable analysis for predictors/ Just the target exposure assessed

4) Only one associated exposure with the outcome in the multivariable analysis.

- Wrong Outcome

5) Not MACE/MACCE

6) Mortality only

7) In-hospital only

- Duplicate

8) Same registry/population as the included studies

| ID | Study | Population | | Exposure | | Outcome | | | Registry |
| --- | --- | --- | --- | --- | --- | --- | --- | --- | --- |
|  |  | 1 | 2 | 3 | 4 | 5 | 6 | 7 | 8 |
| 1 | (Zhang et al., 2023) |  |  |  |  |  |  |  | ☒ |
| 2 | (Yeh et al., 2023) |  |  | ☒ |  |  |  |  |  |
| 3 | (Van de Werf et al., 2023) | ☒ |  |  |  |  |  |  |  |
| 4 | (Räsänen et al., 2023) |  | ☒ |  |  |  |  |  |  |
| 5 | (Park et al., 2023) |  |  |  |  |  |  | ☒ |  |
| 6 | (Nakamura et al., 2023) |  |  |  |  |  |  |  | ☒ |
| 7 | (Mangalesh et al., 2023) |  |  | ☒ |  |  |  |  |  |
| 8 | (Lu et al., 2023) |  |  | ☒ |  |  |  |  |  |
| 9 | (Lang et al., 2023) |  |  | ☒ |  |  |  |  |  |
| 10 | (Kiliç et al., 2023) |  |  |  |  |  |  | ☒ |  |
| 11 | (Jeong et al., 2023) |  |  |  |  | ☒ |  |  |  |
| 12 | (Jan et al., 2023) |  |  |  |  |  |  | ☒ |  |
| 13 | (Horikoshi et al., 2023) |  | ☒ |  |  |  |  |  |  |
| 14 | (Emre et al., 2023) |  |  |  |  |  | ☒ |  |  |
| 15 | (Doolub et al., 2023) |  | ☒ |  |  |  |  |  |  |
| 16 | (Balaji Srinivasan et al., 2023) |  |  | ☒ |  |  |  |  |  |
| 17 | (Bainey et al., 2023) |  | ☒ |  |  |  |  |  |  |
| 18 | (Ahn et al., 2023) |  |  | ☒ |  |  |  |  |  |
| 19 | (Zhang, Zhai, et al., 2022) |  |  |  |  |  | ☒ |  |  |
| 20 | (Zhang, Peng, et al., 2022) |  |  | ☒ |  |  |  |  |  |
| 21 | (Xu et al., 2022) |  |  |  | ☒ |  |  |  |  |
| 22 | (Rozenfeld et al., 2022) |  |  |  |  |  | ☒ |  |  |
| 23 | (Oh et al., 2022) | ☒ |  |  |  |  |  |  |  |
| 24 | (Liu et al., 2022) | ☒ |  |  |  |  |  |  |  |
| 25 | (Lee et al., 2022) |  | ☒ |  |  |  |  |  |  |
| 26 | (Kim et al., 2022) |  |  | ☒ |  |  |  |  |  |
| 27 | (de la Torre Hernandez et al., 2022) |  |  |  |  |  |  |  | ☒ |
| 28 | (D'Ascenzo et al., 2022) |  |  | ☒ |  |  |  |  |  |
| 29 | (Córdoba-Soriano et al., 2022) |  |  | ☒ |  |  |  |  |  |
| 30 | (Wong et al., 2021) |  |  |  |  | ☒ |  |  |  |
| 31 | (Wang et al., 2021) |  |  |  |  |  |  |  | ☒ |
| 32 | (Tamez et al., 2021) |  |  | ☒ |  |  |  |  |  |
| 33 | (Sawayama et al., 2021) |  |  |  |  |  |  | ☒ |  |
| 34 | (Sanz-Girgas et al., 2021) | ☒ |  |  |  |  |  |  |  |
| 35 | (Phan et al., 2021) | ☒ |  |  |  |  |  |  |  |
| 36 | (Ono et al., 2021) | ☒ |  |  |  |  |  |  |  |
| 37 | (Nishihira et al., 2021) |  |  |  |  |  | ☒ |  |  |
| 38 | (Moon et al., 2021) |  |  | ☒ |  |  |  |  |  |
| 39 | (Lafont et al., 2021) |  |  | ☒ |  |  |  |  |  |
| 40 | (Kurobe et al., 2021) |  | ☒ |  |  |  |  |  |  |
| 41 | (Kumar et al., 2021) |  |  |  |  |  | ☒ |  |  |
| 42 | (Kedhi et al., 2021) |  |  | ☒ |  |  |  |  |  |
| 43 | (Joshi et al., 2021) |  | ☒ |  |  |  |  |  |  |
| 44 | (Fu et al., 2021) |  |  |  |  |  | ☒ |  |  |
| 45 | (De Rosa et al., 2021) |  |  |  |  |  | ☒ |  |  |
| 46 | (de Miguel-Yanes et al., 2021) | ☒ |  |  |  |  |  |  |  |
| 47 | (Y. Chen et al., 2021) |  | ☒ |  |  |  |  |  |  |
| 48 | (L. Chen et al., 2021) |  |  |  |  |  | ☒ |  |  |
| 49 | (Cha et al., 2021) |  |  | ☒ |  |  |  |  |  |
| 50 | (Cepas-Guillen et al., 2021) | ☒ |  |  |  |  |  |  |  |
| 51 | (Căruntu et al., 2021) |  |  |  |  | ☒ |  |  |  |
| 52 | (Bianco et al., 2021) |  |  |  |  | ☒ |  |  |  |
| 53 | (Tomaniak et al., 2020) |  |  | ☒ |  |  |  |  |  |
| 54 | (Ramakrishna et al., 2020) |  |  | ☒ |  |  |  |  |  |
| 55 | (Nguyen et al., 2020) | ☒ |  |  |  |  |  |  |  |
| 56 | (Mahadevappa et al., 2020) |  |  | ☒ |  |  |  |  |  |
| 57 | (Li et al., 2020) |  |  |  |  |  | ☒ |  |  |
| 58 | (Lee et al., 2020) |  | ☒ |  |  |  |  |  |  |
| 59 | (Krivosheeva et al., 2020) |  | ☒ |  |  |  |  |  |  |
| 60 | (He et al., 2020) |  |  | ☒ |  |  |  |  |  |
| 61 | (Gimbel et al., 2020) | ☒ |  |  |  |  |  |  |  |
| 62 | (Fernández-Bergés et al., 2020) | ☒ |  |  |  |  |  |  |  |
| 63 | (Dong et al., 2020) |  | ☒ |  |  |  |  |  |  |
| 64 | (De Rosa et al., 2020) |  |  | ☒ |  |  |  |  |  |
| 65 | (De Luca, Verdoia, Savonitto, Piatti, et al., 2020) |  |  |  |  | ☒ |  |  |  |
| 66 | (De Luca, Verdoia, Savonitto, Ferri, et al., 2020) |  |  |  |  | ☒ |  |  |  |
| 67 | (Zheng et al., 2019) |  |  | ☒ |  |  |  |  |  |
| 68 | (Su et al., 2019) |  |  |  |  |  |  | ☒ |  |
| 69 | (Sliman et al., 2019) | ☒ |  |  |  |  |  |  |  |
| 70 | (Shih et al., 2019) |  |  |  |  |  | ☒ |  |  |
| 71 | (Schmucker et al., 2019) |  |  | ☒ |  |  |  |  |  |
| 72 | (Rencuzogullari et al., 2019) |  |  |  |  |  | ☒ |  |  |
| 73 | (Park et al., 2019) | ☒ |  |  |  |  |  |  |  |
| 74 | (Hermans et al., 2019) |  |  |  |  | ☒ |  |  |  |
| 75 | (Guo et al., 2019) | ☒ |  |  |  |  |  |  |  |
| 76 | (Fukuoka et al., 2019) |  |  |  |  |  | ☒ |  |  |
| 77 | (Zhang et al., 2018) |  |  |  |  |  | ☒ |  |  |
| 78 | (Varenne et al., 2018) |  |  | ☒ |  |  |  |  |  |
| 79 | (Silva et al., 2018) |  |  |  |  |  | ☒ |  |  |
| 80 | (Rumiz et al., 2018) |  |  |  | ☒ |  |  |  |  |
| 81 | (Nakamura et al., 2018) |  | ☒ |  |  |  |  |  |  |
| 82 | (Leistner, Münch, Steiner, Jakob, et al., 2018) |  |  |  |  |  | ☒ |  |  |
| 83 | (Leistner, Münch, Steiner, Erbay, et al., 2018) |  |  | ☒ |  |  |  |  |  |
| 84 | (Kim et al., 2018) |  | ☒ |  |  |  |  |  |  |
| 85 | (Effron et al., 2018) |  | ☒ |  |  |  |  |  |  |
| 86 | (Duggal et al., 2018) |  | ☒ |  |  |  |  |  |  |
| 87 | (Cenko et al., 2018) |  |  |  |  |  | ☒ |  |  |
| 88 | (Valle et al., 2017) |  |  | ☒ |  |  |  |  |  |
| 89 | (Sappa et al., 2017) |  |  |  | ☒ |  |  |  |  |
| 90 | (Roubelakis et al., 2017) | ☒ |  |  |  |  |  |  |  |
| 91 | (Qaderdan et al., 2017) |  |  | ☒ |  |  |  |  |  |
| 92 | (Nammas et al., 2017) |  |  |  | ☒ |  |  |  |  |
| 93 | (Kim et al., 2017) |  |  |  | ☒ |  |  |  |  |
| 94 | (Kang et al., 2017) |  | ☒ |  |  |  |  |  |  |
| 95 | (Jin et al., 2017) |  |  |  |  |  |  | ☒ |  |
| 96 | (Hejazi et al., 2017) |  | ☒ |  |  |  |  |  |  |
| 97 | (He et al., 2017) |  | ☒ |  |  |  |  |  |  |
| 98 | (Harada et al., 2017) |  |  |  |  |  |  |  | ☒ |
| 99 | (Won et al., 2016) | ☒ |  |  |  |  |  |  |  |
| 100 | (Ueki et al., 2016) |  | ☒ |  |  |  |  |  |  |
| 101 | (Tong et al., 2016) |  | ☒ |  |  |  |  |  |  |
| 102 | (Son et al., 2016) |  | ☒ |  |  |  |  |  |  |
| 103 | (Sigurjonsdottir et al., 2016) | ☒ |  |  |  |  |  |  |  |
| 104 | (Moon et al., 2016) |  |  |  | ☒ |  |  |  |  |
| 105 | (Jomaa et al., 2016) |  |  |  |  |  |  | ☒ |  |
| 106 | (Hamonangan et al., 2016) |  |  | ☒ |  |  |  |  |  |
| 107 | (Bromage et al., 2016) |  |  |  |  |  | ☒ |  |  |
| 108 | (Badings et al., 2016) |  |  | ☒ |  |  |  |  |  |
| 109 | (Ali et al., 2016) |  | ☒ |  |  |  |  |  |  |
| 110 | (Su et al., 2015) |  | ☒ |  |  |  |  |  |  |
| 111 | (Spyridopoulos et al., 2015) |  |  |  |  |  | ☒ |  |  |
| 112 | (Rynkowska-Kidawa et al., 2015) |  |  |  |  |  |  | ☒ |  |
| 113 | (Morici et al., 2015) |  |  |  |  | ☒ |  |  |  |
| 114 | (Y. Liu et al., 2015) |  |  | ☒ |  |  |  |  |  |
| 115 | (X. M. Liu et al., 2015) |  |  |  |  |  | ☒ |  |  |
| 116 | (Lim et al., 2015) |  |  |  |  |  | ☒ |  |  |
| 117 | (Kim et al., 2015) |  |  |  |  |  |  | ☒ |  |
| 118 | (He et al., 2015) |  |  |  |  |  |  | ☒ |  |
| 119 | (D'Avila et al., 2015) |  |  | ☒ |  |  |  |  |  |
| 120 | (Cantor et al., 2015) | ☒ |  |  |  |  |  |  |  |
| 121 | (Barywani et al., 2015) |  |  |  |  |  | ☒ |  |  |
| 122 | (Anzai et al., 2015) |  |  |  |  |  | ☒ |  |  |
| 123 | (Wang et al., 2014)^[[1]](#footnote-1)^ |  |  | ☒ |  |  |  |  |  |
| 124 | (Velders et al., 2014) |  |  |  |  | ☒ |  |  |  |
| 125 | (Tanaka et al., 2014) |  | ☒ |  |  |  |  |  |  |
| 126 | (Rodriguez-Leor et al., 2014) |  |  |  |  |  |  | ☒ |  |
| 127 | (Rittger et al., 2014) |  | ☒ |  |  |  |  |  |  |
| 128 | (Miura et al., 2014) |  |  |  |  |  |  | ☒ |  |
| 129 | (Kaneko et al., 2014) |  |  |  |  |  | ☒ |  |  |
| 130 | (Gao et al., 2014) |  |  | ☒ |  |  |  |  |  |
| 131 | (De Belder et al., 2014) |  |  | ☒ |  |  |  |  |  |
| 132 | (Bu et al., 2014) |  |  | ☒ |  |  |  |  |  |
| 133 | (Zhang et al., 2013) |  |  |  |  |  |  | ☒ |  |
| 134 | (Vandecasteele et al., 2013) |  |  |  |  |  | ☒ |  |  |
| 135 | (Sinning et al., 2013) |  |  | ☒ |  |  |  |  |  |
| 136 | (Sillano et al., 2013) |  |  | ☒ |  |  |  |  |  |
| 137 | (Schröder et al., 2013) |  |  |  |  |  | ☒ |  |  |
| 138 | (Puymirat et al., 2013) |  |  | ☒ |  |  |  |  |  |
| 139 | (Mahabaleshwarkar et al., 2013) |  |  | ☒ |  |  |  |  |  |
| 140 | (Khera et al., 2013) |  |  |  |  |  | ☒ |  |  |
| 141 | (Fosbol et al., 2013) |  |  | ☒ |  |  |  |  |  |
| 142 | (Ekmekci et al., 2013) |  |  |  |  |  |  | ☒ |  |
| 143 | (Dall'Orto et al., 2013) |  | ☒ |  |  |  |  |  |  |
| 144 | (Caballero et al., 2013) |  |  |  | ☒ |  |  |  |  |
| 145 | (Wang et al., 2012) |  |  | ☒ |  |  |  |  |  |
| 146 | (Murphy et al., 2012) |  | ☒ |  |  |  |  |  |  |
| 147 | (Medina et al., 2012) |  |  |  |  |  | ☒ |  |  |
| 148 | (Marcolino et al., 2012) |  |  | ☒ |  |  |  |  |  |
| 149 | (Jinnouchi et al., 2012) |  |  |  |  |  |  | ☒ |  |
| 150 | (Hillegass et al., 2012) |  |  |  |  | ☒ |  |  |  |
| 151 | (Thomas et al., 2011) |  | ☒ |  |  |  |  |  |  |
| 152 | (Shirasawa et al., 2011) |  |  |  |  |  | ☒ |  |  |
| 153 | (Rymuza et al., 2011) | ☒ |  |  |  |  |  |  |  |
| 154 | (Poorhosseini et al., 2011) |  |  | ☒ |  |  |  |  |  |
| 155 | (Oqueli & Dick, 2011) |  |  |  |  |  |  | ☒ |  |
| 156 | (Maekawa et al., 2011) |  |  | ☒ |  |  |  |  |  |
| 157 | (Kukreja et al., 2011) |  | ☒ |  |  |  |  |  |  |
| 158 | (Kuch et al., 2011) | ☒ |  |  |  |  |  |  |  |
| 159 | (Hafiz et al., 2011) |  | ☒ |  |  |  |  |  |  |
| 160 | (De Felice et al., 2011) |  | ☒ |  |  |  |  |  |  |
| 161 | (Bueno et al., 2011) | ☒ |  |  |  |  |  |  |  |
| 162 | (Sheridan et al., 2010) | ☒ |  |  |  |  |  |  |  |
| 163 | (Nowak et al., 2010) |  |  | ☒ |  |  |  |  |  |
| 164 | (Koutouzis et al., 2010) |  |  | ☒ |  |  |  |  |  |
| 165 | (Kiatchoosakun et al., 2010) |  |  |  |  |  |  | ☒ |  |
| 166 | (Johnman et al., 2010) |  |  | ☒ |  |  |  |  |  |
| 167 | (Danzi et al., 2010) |  |  |  |  |  | ☒ |  |  |
| 168 | (Varani et al., 2009) |  | ☒ |  |  |  |  |  |  |
| 169 | (Poludasu et al., 2009) |  | ☒ |  |  |  |  |  |  |
| 170 | (Lim et al., 2009) |  | ☒ |  |  |  |  |  |  |
| 171 | (Guo et al., 2009) |  |  | ☒ |  |  |  |  |  |
| 172 | (Harjai et al., 2008) |  | ☒ |  |  |  |  |  |  |
| 173 | (Ciszewski et al., 2008) |  |  |  |  |  | ☒ |  |  |
| 174 | (Wiemer et al., 2007) | Incomplete data; No estimates for predictors were provided. | | | | | | | |
| 175 | (Wenaweser et al., 2007) |  | ☒ |  |  |  |  |  |  |
| 176 | (Teplitsky et al., 2007) |  |  |  |  |  | ☒ |  |  |
| 177 | (Malpica et al., 2007) |  |  | ☒ |  |  |  |  |  |
| 178 | (Kabbani et al., 2007) |  |  |  |  |  |  | ☒ |  |
| 179 | (Wang et al., 2006) |  |  | ☒ |  |  |  |  |  |
| 180 | (Hassani et al., 2006) |  |  |  |  |  | ☒ |  |  |
| 181 | (Floyd et al., 2006) |  | ☒ |  |  |  |  |  |  |
| 182 | (Feldman et al., 2006) |  |  |  |  |  | ☒ |  |  |
| 183 | (Prasad et al., 2004) |  |  |  |  |  | ☒ |  |  |
| 184 | (Mehta et al., 2004) | ☒ |  |  |  |  |  |  |  |
| 185 | (Iwashita et al., 2004) | ☒ |  |  |  |  |  |  |  |
| 186 | (Guagliumi et al., 2004) |  | ☒ |  |  |  |  |  |  |
| 187 | (Sadeghi et al., 2003) |  |  |  |  |  |  | ☒ |  |
| 188 | (Eckart et al., 2003) |  |  | ☒ |  |  |  |  |  |
| 189 | (Dynina et al., 2003) |  |  |  |  |  |  | ☒ |  |
| 190 | (Fuchs et al., 2002) |  | ☒ |  |  |  |  |  |  |
| 191 | (McGrath et al., 2000) |  |  | ☒ |  |  |  |  |  |
| 192 | (Ang et al., 2000) |  |  |  |  |  |  | ☒ |  |

**Publication bias**

| **Predictors** | **Egger’s coefficient** | **95% confidence interval** | **P-value** |
| --- | --- | --- | --- |
| Female sex | -0.097 | -2.84, 2.64 | 0.94 |
| Age | 0.211 | -1.87, 2.29 | 0.84 |
| Diabetes | 1.074 | -0.65, 2.80 | 0.25 |
| Hypertension | 0.492 | -3.11, 4.10 | 0.80 |
| STEMI | 1.548 | -1.07, 4.16 | 0.33 |
| GFR | -2.427 | -4.32, -0.54 | 0.08 |
| Lower EF | -0.372 | -4.93, 4.18 | 0.88 |
| LMCA | -0.834 | -5.25, 3.58 | 0.73 |
| Successful PCI | -0.932 | -5.46, 3.60 | 0.70 |

**Supplementary Figure**


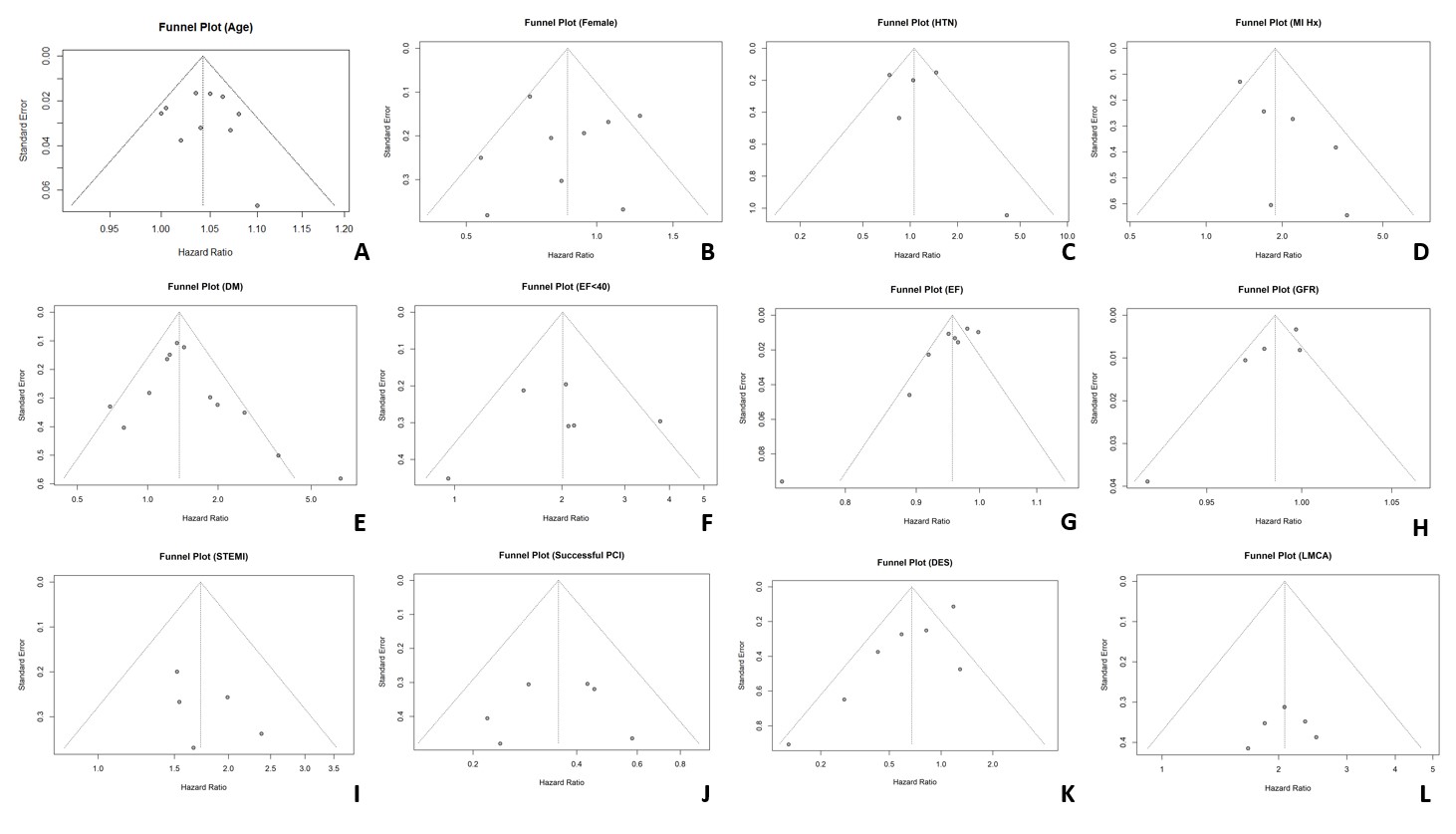


**Supplementary Figure S1.** Funnel plots for publication bias. A: age, B: female, C: hypertension (HTN), D: history of myocardial infarction (MI), E: diabetes, F: ejection fraction (EF)<40, G: continuous EF, H: glomerular filtration rate (GFR), I: ST-elevation MI, J: successful percutaneous coronary intervention, K: drug-eluting stents, L: left main coronary artery disease;

**References**

Ahn, W. J., Rha, S. W., Choi, B. G., Jeong, M. H., Ahn, T. H., Yoon, J., Kim, H. S., Seung, K. B., Gwon, H. C., Chae, S. C., Kim, C. J., Cha, K. S., Lee, J. H., Chae, J. K., Joo, S. J., Yoon, C. H., Hur, S. H., Seong, I. W., Hwang, K. K., . . . Hwang, J. Y. (2023). The impact of angiotensin-converting-enzyme inhibitors versus angiotensin receptor blockers on 3-year clinical outcomes in elderly (≥ 65) patients with acute myocardial infarction without hypertension [Article]. *Heart and Vessels*, *38*(7), 898-908. <https://doi.org/10.1007/s00380-023-02244-x>

Ali, Z. A., Qureshi, Y. H., Galougahi, K. K., Poludasu, S., Roy, S., Krishnan, P., Zalewski, A., Shah, Z. Z., Bhatti, N., Kalapatapu, K., Mehran, R., Dangas, G., Kini, A. S., & Sharma, S. K. (2016). Effects of baseline and early acquired thrombocytopaenia on long-term mortality in patients undergoing percutaneous coronary intervention with bivalirudin [Article]. *EuroIntervention*, *11*(14), e1627-e1638. <https://doi.org/10.4244/EIJV11I14A314>

Ang, P. C., Farouque, H. M., Harper, R. W., & Meredith, I. T. (2000). Percutaneous coronary intervention in the elderly: a comparison of procedural and clinical outcomes between the eighth and ninth decades. *J Invasive Cardiol*, *12*(10), 488-494.

Anzai, A., Maekawa, Y., Kodaira, M., Mogi, S., Arai, T., Kawakami, T., Kanazawa, H., Hayashida, K., Yuasa, S., Kawamura, A., & Fukuda, K. (2015). Prognostic implications of optimal medical therapy in patients undergoing percutaneous coronary intervention for acute coronary syndrome in octogenarians. *Heart Vessels*, *30*(2), 186-192. <https://doi.org/10.1007/s00380-014-0474-y>

Badings, E. A., Remkes, W. S., Dambrink, J. H., The, S. H., Van Wijngaarden, J., Tjeerdsma, G., Rasoul, S., Timmer, J. R., van der Wielen, M. L., Lok, D. J., & van 't Hof, A. W. (2016). Timing of intervention in high-risk non-ST-elevation acute coronary syndromes in PCI versus non-PCI centres : Sub-group analysis of the ELISA-3 trial. *Neth Heart J*, *24*(3), 181-187. <https://doi.org/10.1007/s12471-015-0801-7>

Bainey, K. R., Wood, D. A., Bossard, M., Campo, G., Cantor, W. J., Lavi, S., Madan, M., Mehran, R., Pinilla-Echeverri, N., Rao, S., Sarma, J., Sheth, T., Stankovic, G., Steg, P. G., Storey, R. F., Tanguay, J. F., Velianou, J. L., Welsh, R. C., Mani, T., . . . Mehta, S. R. (2023). Effects of Complete Revascularization According to Age in Patients with ST-Segment Elevation Myocardial Infarction and Multivessel Disease (COMPLETE-AGE). *Am Heart J*. <https://doi.org/10.1016/j.ahj.2023.10.004>

Balaji Srinivasan, S., Sehly, A., Jaltotage, B., Qin, S., Ihdayhid, A. R., Marangou, J., Rankin, J. M., Sanfilippo, F. M., & Dwivedi, G. (2023). Short-term DAPT after coronary stenting has similar ischemic and bleeding outcomes as long-term DAPT: a 5-year population-based cohort study. *Ir J Med Sci*, *192*(4), 1645-1647. <https://doi.org/10.1007/s11845-022-03171-y>

Barywani, S. B., Li, S., Lindh, M., Ekelund, J., Petzold, M., Albertsson, P., Lund, L. H., & Fu, M. L. (2015). Acute coronary syndrome in octogenarians: association between percutaneous coronary intervention and long-term mortality. *Clin Interv Aging*, *10*, 1547-1553. <https://doi.org/10.2147/cia.S89127>

Bianco, M., Careggio, A., Biolè, C. A., Quadri, G., Quiros, A., Raposeiras-Roubin, S., Abu-Assi, E., Kinnaird, T., Ariza-Solè, A., Liebetrau, C., Manzano-Fernàndez, S., Boccuzzi, G., Henriques, J. P. S., Spirito, A., Templin, C., Wilton, S. B., Velicki, L., Correia, L., Rognoni, A., . . . Cerrato, E. (2021). Ticagrelor or Clopidogrel After an Acute Coronary Syndrome in the Elderly: A Propensity Score Matching Analysis from 16,653 Patients Treated with PCI Included in Two Large Multinational Registries. *Cardiovasc Drugs Ther*, *35*(6), 1171-1182. <https://doi.org/10.1007/s10557-021-07213-y>

Bromage, D. I., Jones, D. A., Rathod, K. S., Grout, C., Iqbal, M. B., Lim, P., Jain, A., Kalra, S. S., Crake, T., Astroulakis, Z., Ozkor, M., Rakhit, R. D., Knight, C. J., Dalby, M. C., Malik, I. S., Mathur, A., Redwood, S., MacCarthy, P. A., & Wragg, A. (2016). Outcome of 1051 Octogenarian Patients With ST-Segment Elevation Myocardial Infarction Treated With Primary Percutaneous Coronary Intervention: Observational Cohort From the London Heart Attack Group. *J Am Heart Assoc*, *5*(6). <https://doi.org/10.1161/jaha.115.003027>

Bu, C., Zhao, Y., Ma, H., Han, H., Yang, S., Shi, D., Liu, Y., Fang, Z., Wang, Z., Ma, Q., Hu, B., Yang, Q., Li, Y., Liu, R., Nie, B., & Zhou, Y. (2014). Protective role of statins in patients with acute coronary syndrome aged ≥ 75 years with low LDL-C who underwent percutaneous coronary intervention. *Angiology*, *65*(7), 590-595. <https://doi.org/10.1177/0003319713500379>

Bueno, H., Betriu, A., Heras, M., Alonso, J. J., Cequier, A., García, E. J., López-Sendón, J. L., Macaya, C., & Hernández-Antolín, R. (2011). Primary angioplasty vs. fibrinolysis in very old patients with acute myocardial infarction: TRIANA (TRatamiento del Infarto Agudo de miocardio eN Ancianos) randomized trial and pooled analysis with previous studies. *Eur Heart J*, *32*(1), 51-60. <https://doi.org/10.1093/eurheartj/ehq375>

Caballero, L., Ruiz-Nodar, J. M., Marín, F., Roldán, V., Hurtado, J. A., Valencia, J., Manzano-Fernandez, S., Sogorb, F., Valdes, M., & Lip, G. Y. H. (2013). Oral anticoagulation improves the prognosis of octogenarian patients with atrial fibrillation undergoing percutaneous coronary intervention and stenting [Article]. *Age and Ageing*, *42*(1), 70-75. <https://doi.org/10.1093/ageing/afs121>

Cantor, W. J., Mehta, S. R., Yuan, F., Džavík, V., Worthley, M., Niemelä, K., Valentin, V., Fung, A., Cheema, A. N., Widimsky, P., Natarajan, M., Jedrzejowski, B., & Jolly, S. S. (2015). Radial versus femoral access for elderly patients with acute coronary syndrome undergoing coronary angiography and intervention: insights from the RIVAL trial. *Am Heart J*, *170*(5), 880-886. <https://doi.org/10.1016/j.ahj.2015.08.011>

Căruntu, F., Bordejevic, D. A., Buz, B., Gheorghiu, A., & Tomescu, M. C. (2021). Independent predictors of in-hospital and 1-year mortality rates in octogenarians with acute myocardial infarction. *Rev Cardiovasc Med*, *22*(2), 489-497. <https://doi.org/10.31083/j.rcm2202056>

Cenko, E., Yoon, J., Kedev, S., Stankovic, G., Vasiljevic, Z., Krljanac, G., Kalpak, O., Ricci, B., Milicic, D., Manfrini, O., van der Schaar, M., Badimon, L., & Bugiardini, R. (2018). Sex Differences in Outcomes After STEMI: Effect Modification by Treatment Strategy and Age. *JAMA Intern Med*, *178*(5), 632-639. <https://doi.org/10.1001/jamainternmed.2018.0514>

Cepas-Guillen, P. L., Echarte-Morales, J., Flores-Umanzor, E., Fernandez-Valledor, A., Caldentey, G., Viana-Tejedor, A., Martinez Gomez, E., Tundidor-Sanz, E., Borrego-Rodriguez, J., Vidal, P., Llagostera, M., Quiroga, X., Freixa, X., Fernández-Vázquez, F., & Sabate, M. (2021). Sex-gender disparities in nonagenarians with acute coronary syndrome. *Clin Cardiol*, *44*(3), 371-378. <https://doi.org/10.1002/clc.23545>

Cha, J. J., Park, J. H., Joo, H. J., Hong, S. J., Ahn, T. H., Kim, B. K., Shin, W., Ahn, S. G., Yoon, J., Kim, Y. H., Cho, Y. H., Kang, W. C., Kim, W., Lim, Y. H., Gwon, H. C., Choi, W. G., & Lim, D. S. (2021). Impact of genetic variants on clinical outcome after percutaneous coronary intervention in elderly patients. *Aging (Albany NY)*, *13*(5), 6506-6524. <https://doi.org/10.18632/aging.202799>

Chen, L., Huang, Z., Lu, J., Yang, Y., Pan, Y., Bao, K., Wang, J., Chen, W., Liu, J., Liu, Y., Chen, K., Li, W., & Chen, S. (2021). Impact of the Malnutrition on Mortality in Elderly Patients Undergoing Percutaneous Coronary Intervention. *Clin Interv Aging*, *16*, 1347-1356. <https://doi.org/10.2147/cia.S308569>

Chen, Y., Han, M., Zheng, Y. Y., Zhu, F., Aisan, A., Maheshati, T., Ma, Y. T., & Xie, X. (2021). Model for End-Stage Liver Disease Score Predicts the Mortality of Patients with Coronary Heart Disease Who Underwent Percutaneous Coronary Intervention [Article]. *Cardiology Research and Practice*, *2021*, Article 6401092. <https://doi.org/10.1155/2021/6401092>

Ciszewski, A., Karcz, M., Kȩpka, C., Bekta, P., Ksiȩzycka, E., Przyłuski, J., Dȩbski, A., Witkowski, A., & Ruzyłło, W. (2008). Primary angioplasty in patients ≥75 years old with ST-elevation myocardial infarction - One-year follow-up results [Article]. *Kardiologia Polska*, *66*(8), 828-833. <https://www.embase.com/search/results?subaction=viewrecord&id=L352522649&from=export>

Córdoba-Soriano, J. G., Gutiérrez-Díez, A., Del Blanco, B. G., Núñez, J., Amat-Santos, I. J., Oteo, J. F., Romaguera, R., Gallardo-López, A., Lozano Ruíz-Poveda, F., Baello, P., Aguar, P., Jerez-Valero, M., Jiménez-Díaz, V. A., Serra, B., Cascon, J. D., Morales-Ponce, F. J., Portero-Portaz, J. J., Melehi El Assali, D., Cerrato-García, P., & Jiménez-Mazuecos, J. (2022). Bioactive or Drug-Eluting Stents in 75 Years or Older Patients: The BIODES-75 Registry. *Cardiovasc Revasc Med*, *42*, 114-120. <https://doi.org/10.1016/j.carrev.2022.02.001>

D'Ascenzo, F., Elia, E., de Filippo, O., Manai, R., Breviario, S., Bruno, F., Iannaccone, M., Wańha, W., Bianco, M., Patti, G., Raposeiras-Roubin, S., Abu-Assi, E., Bo, M., De Ferrari, G. M., & Conrotto, F. (2022). Net clinical benefit of different strategies of dual antiplatelet therapy in elderly patients: Data from the praise registry. *Int J Cardiol*, *353*, 9-14. <https://doi.org/10.1016/j.ijcard.2022.01.019>

D'Avila, A. C., Filho, R. R., Schmidt, M. M., Melleu, K., de Oliveira Cardoso, C., Gottschall, C. A. M., de Quadros, A. S., & Azmus, A. D. (2015). Primary coronary angioplasty in patients over 80 years of age [Article]. *Revista Brasileira de Cardiologia Invasiva*, *23*(4), 261-265. <https://doi.org/10.1016/j.rbciev.2017.02.012>

Dall'Orto, C. C., Ferreira Lopes, R. P., Teixeira Alcântara, C., Cisari, G., De Souza Marques, A., Castilho Perea, J. C., & Silveira Costa, G. O. (2013). Percutaneous coronary intervention using transradial access in elderly vs. non-elderly patients [Article]. *Revista Brasileira de Cardiologia Invasiva*, *21*(1), 36-42. <https://doi.org/10.1016/s0104-1843(13)50009-9>

Danzi, G. B., Centola, M., Pomidossi, G. A., Consonni, D., De Matteis, S., Stabile, A., Sesana, M., Anzuini, A., Sganzerla, P., Cortese, B., Migliorini, A., & Antoniucci, D. (2010). Usefulness of primary angioplasty in nonagenarians with acute myocardial infarction. *Am J Cardiol*, *106*(6), 770-773. <https://doi.org/10.1016/j.amjcard.2010.04.041>

De Belder, A., De La Torre Hernandez, J. M., Lopez-Palop, R., O'Kane, P., Hernandez, F. H., Strange, J., Gimeno, F., Cotton, J., Diaz Fernandez, J. F., Carrillo Saez, P., Thomas, M., Pinar, E., Curzen, N., Baz, J. A., Cooter, N., Lozano, I., Skipper, N., Robinson, D., & Hildick-Smith, D. (2014). A prospective randomized trial of everolimus-eluting stents versus bare-metal stents in octogenarians: The XIMA trial (Xience or vision stents for the management of angina in the elderly) [Article]. *Journal of the American College of Cardiology*, *63*(14), 1371-1375. <https://doi.org/10.1016/j.jacc.2013.10.053>

De Felice, F., Fiorilli, R., Parma, A., Musto, C., Nazzaro, M. S., Confessore, P., Scappaticci, M., Cifarelli, A., & Violini, R. (2011). Comparison of one-year outcome of patients aged <75 years versus <75 years undergoing "rescue" percutaneous coronary intervention [Article]. *American Journal of Cardiology*, *108*(8), 1075-1080. <https://doi.org/10.1016/j.amjcard.2011.06.006>

de la Torre Hernandez, J. M., Palop, R. L., Jimenez Mazuecos, J. M., Sáez, P. C., Gutierez-Barrios, A., Pinar, E., Cid, B., Fernandez, L., Camarero, T. G., Urbano-Carrillo, C., Oteo Dominguez, J. F., Jimenez Diaz, V. A., Gomez Menchero, A. E., Fernández, E. G., Córdoba Soriano, J. G., Ocaranza, R., Úcar, E. A., Roman, K. G. S., Leal, S., . . . de Prado, A. P. (2022). Prospective application of a bleeding and ischemic risks-adjusted antithrombotic protocol in elderly patients revascularized with everolimus-eluting stents: EPIC05-Sierra75 study. *J Geriatr Cardiol*, *19*(5), 354-366. <https://doi.org/10.11909/j.issn.1671-5411.2022.05.009>

De Luca, G., Verdoia, M., Savonitto, S., Ferri, L. A., Piatti, L., Grosseto, D., Morici, N., Bossi, I., Sganzerla, P., Tortorella, G., Cacucci, M., Ferrario, M., Murena, E., Sibilio, G., Tondi, S., Toso, A., Bongioanni, S., Ravera, A., Corrada, E., . . . De Servi, S. (2020). Impact of body mass index on clinical outcome among elderly patients with acute coronary syndrome treated with percutaneous coronary intervention: Insights from the ELDERLY ACS 2 trial. *Nutr Metab Cardiovasc Dis*, *30*(5), 730-737. <https://doi.org/10.1016/j.numecd.2020.01.001>

De Luca, G., Verdoia, M., Savonitto, S., Piatti, L., Grosseto, D., Morici, N., Bossi, I., Sganzerla, P., Tortorella, G., Cacucci, M., Murena, E., Toso, A., Bongioanni, S., Ravera, A., Corrada, E., Mariani, M., Di Ascenzo, L., Petronio, A. S., Cavallini, C., . . . De Servi, S. (2020). Impact of diabetes on clinical outcome among elderly patients with acute coronary syndrome treated with percutaneous coronary intervention: insights from the ELDERLY ACS 2 trial. *J Cardiovasc Med (Hagerstown)*, *21*(6), 453-459. <https://doi.org/10.2459/jcm.0000000000000978>

de Miguel-Yanes, J. M., Jiménez-García, R., Hernandez-Barrera, V., de Miguel-Díez, J., Muñoz-Rivas, N., Méndez-Bailón, M., Pérez-Farinós, N., López-Herranz, M., & Lopez-de-Andres, A. (2021). Sex Differences in the Incidence and Outcomes of Acute Myocardial Infarction in Spain, 2016-2018: A Matched-Pair Analysis. *J Clin Med*, *10*(8). <https://doi.org/10.3390/jcm10081795>

De Rosa, R., Morici, N., De Luca, G., De Luca, L., Ferri, L. A., Piatti, L., Tortorella, G., Grosseto, D., Franco, N., Misuraca, L., Sganzerla, P., Cacucci, M., Antonicelli, R., Cavallini, C., Lenatti, L., Leuzzi, C., Murena, E., Ravera, A., Ferrario, M., . . . Savonitto, S. (2021). Association of Sex with Outcome in Elderly Patients with Acute Coronary Syndrome Undergoing Percutaneous Coronary Intervention. *Am J Med*, *134*(9), 1135-1141.e1131. <https://doi.org/10.1016/j.amjmed.2021.03.025>

De Rosa, R., Morici, N., De Servi, S., De Luca, G., Galasso, G., Piscione, F., Ferri, L. A., Piatti, L., Grosseto, D., Tortorella, G., Franco, N., Lenatti, L., Misuraca, L., Leuzzi, C., Verdoia, M., Sganzerla, P., Cacucci, M., Ferrario, M., Murena, E., . . . Savonitto, S. (2020). Impact of renal dysfunction and acute kidney injury on outcome in elderly patients with acute coronary syndrome undergoing percutaneous coronary intervention. *Eur Heart J Acute Cardiovasc Care*. <https://doi.org/10.1177/2048872620920475>

Dong, H., Hachinohe, D., Nie, Z., Kashima, Y., Luo, J., Haraguchi, T., Shitan, H., Watanabe, T., Tadano, Y., Kaneko, U., Sugie, T., Kobayashi, K., Kanno, D., Enomoto, M., Sato, K., & Fujita, T. (2020). Reappraisal Value of a Modified Rotational Atherectomy Technique in Contemporary Coronary Angioplasty Era. *J Interv Cardiol*, *2020*, 9190702. <https://doi.org/10.1155/2020/9190702>

Doolub, G., Tonino, P. A. L., Kedev, S., Monségu, J., Paradies, V., Austin, D., Spanó, F., Roffi, M., Fröbert, O., von Birgelen, C., Buchanan, L., & Mamas, M. A. P. (2023). Impact of Sex on Clinical Outcomes in Patients undergoing Complex Percutaneous Coronary Angioplasty (from the e-ULTIMASTER Study). *Am J Cardiol*, *186*, 71-79. <https://doi.org/10.1016/j.amjcard.2022.10.023>

Duggal, B., Subramanian, J., Duggal, M., Singh, P., Rajivlochan, M., Saunik, S., Desiraju, K., Avhad, A., Ram, U., Sen, S., & Agrawal, A. (2018). Survival outcomes post percutaneous coronary intervention: Why the hype about stent type? Lessons from a healthcare system in India. *PLoS ONE*, *13*(5), e0196830. <https://doi.org/10.1371/journal.pone.0196830>

Dynina, O., Vakili, B. A., Slater, J. N., Sherman, W., Ravi, K. L., Green, S. J., Sanborn, T. A., & Brown, D. L. (2003). In-hospital outcomes of contemporary percutaneous coronary interventions in the very elderly. *Catheter Cardiovasc Interv*, *58*(3), 351-357. <https://doi.org/10.1002/ccd.10437>

Eckart, R. E., Shry, E. A., Simpson, D. E., & Stajduhar, K. C. (2003). Percutaneous coronary intervention in the elderly: procedural success and 1-year outcomes. *Am J Geriatr Cardiol*, *12*(6), 366-368. <https://doi.org/10.1111/j.1076-7460.2003.02505.x>

Effron, M. B., Nair, K. V., Molife, C., Keller, S. Y., Page, R. L., 2nd, Simeone, J. C., Murphy, B., Nordstrom, B. L., Zhu, Y., McCollam, P. L., & Vetrovec, G. W. (2018). One-Year Clinical Effectiveness Comparison of Prasugrel with Ticagrelor: Results from a Retrospective Observational Study using an Integrated Claims Database. *Am J Cardiovasc Drugs*, *18*(2), 129-141. <https://doi.org/10.1007/s40256-017-0255-y>

Ekmekci, A., Uluganyan, M., Tufan, F., Uyarel, H., Karaca, G., Kul, S., Gungor, B., Ertas, G., Erer, B., Sayar, N., Gul, M., & Eren, M. (2013). Impact of admission blood glucose levels on prognosis of elderly patients with ST elevation myocardial infarction treated by primary percutaneous coronary intervention. *J Geriatr Cardiol*, *10*(4), 310-316. <https://doi.org/10.3969/j.issn.1671-5411.2013.04.002>

Emre, E., Hancı, K., Gökçek, M. D., Aktaş, M., Kalaycıoğlu, E., Çetin, M., Karaüzüm, K., Karaüzüm, İ., & Ural, E. (2023). Thyroid Functions Are Associated with All-Cause Long-Term Mortality in Elderly Patients with ST-Segment Elevation Myocardial Infarction Undergoing Primary Percutaneous Coronary Intervention. *Turk Kardiyol Dern Ars*, *51*(6), 387-393. <https://doi.org/10.5543/tkda.2023.53389> (Primer Perkütan Koroner Girişim Uygulanan ST Segmenti Yükselmeli Miyokard Enfarktüslü Yaşlı Hastalarda Tiroid Fonksiyonları Tüm Nedenlere Bağlı Uzun Dönem Mortalite ile İlişkilidir.)

Feldman, D. N., Gade, C. L., Slotwiner, A. J., Parikh, M., Bergman, G., Wong, S. C., & Minutello, R. M. (2006). Comparison of Outcomes of Percutaneous Coronary Interventions in Patients of Three Age Groups (<60, 60 to 80, and >80 Years) (from the New York State Angioplasty Registry†)†This study was performed with the permission of the New York State Department of Health, which retains ownership of the Registry [Article]. *American Journal of Cardiology*, *98*(10), 1334-1339. <https://doi.org/10.1016/j.amjcard.2006.06.026>

Fernández-Bergés, D., Degano, I. R., Gonzalez Fernandez, R., Subirana, I., Vila, J., Jiménez-Navarro, M., Perez-Fernandez, S., Roqué, M., Bayes-Genis, A., Fernandez-Aviles, F., Mayorga, A., Bertomeu-Gonzalez, V., Sanchis, J., Rodríguez Esteban, M., Sanchez-Hidalgo, A., Sanchez-Insa, E., Elorriaga, A., Abu Assi, E., Nuñez, A., . . . Marrugat, J. (2020). Benefit of primary percutaneous coronary interventions in the elderly with ST segment elevation myocardial infarction. *Open Heart*, *7*(2). <https://doi.org/10.1136/openhrt-2019-001169>

Floyd, K. C., Jayne, J. E., Kaplan, A. V., Friedman, B. J., Niles, N. W., Hettleman, B. D., Robb, J. F., & Thompson, C. A. (2006). Age-based differences of percutaneous coronary intervention in the drug-eluting stent era. *J Interv Cardiol*, *19*(5), 381-387. <https://doi.org/10.1111/j.1540-8183.2006.00192.x>

Fosbol, E. L., Wang, T. Y., Li, S., Piccini, J., Lopes, R. D., Mills, R. M., Klaskala, W., Thomas, L., Roe, M. T., & Peterson, E. D. (2013). Warfarin use among older atrial fibrillation patients with non-ST-segment elevation myocardial infarction managed with coronary stenting and dual antiplatelet therapy. *Am Heart J*, *166*(5), 864-870. <https://doi.org/10.1016/j.ahj.2013.08.005>

Fu, B., Wei, X., Wang, Q., Yang, Z., Chen, J., & Yu, D. (2021). Use of the Thrombolysis in Myocardial Infarction Risk Index for Elderly Patients With ST-Segment Elevation Myocardial Infarction. *Front Cardiovasc Med*, *8*, 743678. <https://doi.org/10.3389/fcvm.2021.743678>

Fuchs, S., Stabile, E., Kinnaird, T. D., Mintz, G. S., Gruberg, L., Canos, D. A., Pinnow, E. E., Kornowski, R., Suddath, W. O., Satler, L. F., Pichard, A. D., Kent, K. M., & Weissman, N. J. (2002). Stroke complicating percutaneous coronary interventions: incidence, predictors, and prognostic implications. *Circulation*, *106*(1), 86-91. <https://doi.org/10.1161/01.cir.0000020678.16325.e0>

Fukuoka, S., Kurita, T., Dohi, K., Masuda, J., Seko, T., Tanigawa, T., Saito, Y., Kakimoto, H., Makino, K., & Ito, M. (2019). Untangling the obesity paradox in patients with acute myocardial infarction after primary percutaneous coronary intervention (detail analysis by age). *Int J Cardiol*, *289*, 12-18. <https://doi.org/10.1016/j.ijcard.2019.01.011>

Gao, Z., Yuan, J. Q., Xu, B., Yang, Y. J., Chen, J., Chen, J. L., Qiao, S. B., Wu, Y. J., Yan, H. B., & Gao, R. L. (2014). Is being an elderly woman a risk factor for worse outcomes after percutaneous coronary intervention? A large cohort study from one center. *Angiology*, *65*(7), 596-601. <https://doi.org/10.1177/0003319713512940>

Gimbel, M. E., Willemsen, L. M., Daggelders, M. C., Kelder, J. C., Oirbans, T., Beukema, K. F., Daeter, E. J., & Ten Berg, J. M. (2020). Long-term follow-up after bypass surgery or coronary stenting in elderly with multivessel disease. *Neth Heart J*, *28*(9), 467-477. <https://doi.org/10.1007/s12471-020-01415-z>

Guagliumi, G., Stone, G. W., Cox, D. A., Stuckey, T., Tcheng, J. E., Turco, M., Musumeci, G., Griffin, J. J., Lansky, A. J., Mehran, R., Grines, C. L., & Garcia, E. (2004). Outcome in elderly patients undergoing primary coronary intervention for acute myocardial infarction: results from the Controlled Abciximab and Device Investigation to Lower Late Angioplasty Complications (CADILLAC) trial. *Circulation*, *110*(12), 1598-1604. <https://doi.org/10.1161/01.Cir.0000142862.98817.1f>

Guo, F., Wang, X., Li, G., Chen, X., & Jin, Y. (2009). Risk factors of acute myocardial infarction following primary percutaneous coronary intervention among elderly patients [Article]. *Journal of Geriatric Cardiology*, *6*(2), 67-70. <https://www.embase.com/search/results?subaction=viewrecord&id=L360015815&from=export>

Guo, L., Lv, H., Zhong, L., Wu, J., Ding, H., Xu, J., & Huang, R. (2019). Comparison of long-term outcomes of medical therapy and successful recanalisation for coronary chronic total occlusions in elderly patients: a report of 1,294 patients. *Cardiovasc Diagn Ther*, *9*(6), 586-595. <https://doi.org/10.21037/cdt.2019.11.01>

Hafiz, A. M., Jan, M. F., Mori, N., Gupta, A., Bajwa, T., & Allaqaband, S. (2011). Contemporary clinical outcomes of primary percutaneous coronary intervention in elderly versus younger patients presenting with acute ST-segment elevation myocardial infarction. *J Interv Cardiol*, *24*(4), 357-365. <https://doi.org/10.1111/j.1540-8183.2011.00634.x>

Hamonangan, R., Wijaya, I. P., Setiati, S., & Harimurti, K. (2016). Impact of Frailty on the First 30 Days of Major Cardiac Events in Elderly Patients with Coronary Artery Disease Undergoing Elective Percutaneous Coronary Intervention. *Acta Med Indones*, *48*(2), 91-98.

Harada, M., Miura, T., Kobayashi, T., Kobayashi, H., Kobayashi, M., Nakajima, H., Kimura, H., Akanuma, H., Mawatari, E., Sato, T., Hotta, S., Kamiyoshi, Y., Maruyama, T., Watanabe, N., Eisawa, T., Hashizume, N., Ebisawa, S., Miyashita, Y., & Ikeda, U. (2017). Clinical impact of complete revascularization in elderly patients with multi-vessel coronary artery disease undergoing percutaneous coronary intervention: A sub-analysis of the SHINANO registry. *Int J Cardiol*, *230*, 413-419. <https://doi.org/10.1016/j.ijcard.2016.12.093>

Harjai, K., Shenoy, C., Raizada, A., Eswaran, M., Acharji, S., Sattur, S., Orshaw, P., & Devarakonda, S. (2008). Major adverse noncardiac events after PCI as predictors of long-term mortality [Article]. *Journal of Interventional Cardiology*, *21*(5), 395-402. <https://doi.org/10.1111/j.1540-8183.2008.00387.x>

Hassani, S. E., Wolfram, R. M., Kuchulakanti, P. K., Xue, Z., Gevorkian, N., Suddath, W. O., Satler, L. F., Kent, K. M., Pichard, A. D., Weissman, N. J., & Waksman, R. (2006). Percutaneous coronary intervention with drug-eluting stents in octogenarians: characteristics, clinical presentation, and outcomes. *Catheter Cardiovasc Interv*, *68*(1), 36-43. <https://doi.org/10.1002/ccd.20768>

He, J., Zhao, H., Yu, X., Li, Q., Lv, S., Chen, F., & Jiang, T. (2017). SYNTAX score-II predicts long-term mortality in patients who underwent left main percutaneous coronary intervention treated with second-generation drug-eluting stents [Article]. *International Heart Journal*, *58*(3), 344-350. <https://doi.org/10.1536/ihj.16-292>

He, P. Y., Yang, Y. J., Qiao, S. B., Xu, B., Yao, M., Wu, Y. J., Wu, Y., Yuan, J. Q., Chen, J., Liu, H. B., Dai, J., Li, W., Tang, Y. D., Yang, J. G., & Gao, R. L. (2015). Impact of body mass index on the clinical outcomes after percutaneous coronary intervention in patients ≥ 75 years old. *Chin Med J (Engl)*, *128*(5), 638-643. <https://doi.org/10.4103/0366-6999.151662>

He, W., Li, C., Chen, Q., Xiang, T., Wang, P., & Pang, J. (2020). Serum sclerostin and adverse outcomes in elderly patients with stable coronary artery disease undergoing percutaneous coronary intervention. *Aging Clin Exp Res*, *32*(10), 2065-2072. <https://doi.org/10.1007/s40520-019-01393-2>

Hejazi, S. F., Iranirad, L., Doostali, K., Khodadadi, N., & Norouzi, S. (2017). In-Hospital Clinical Outcomes and Procedural Complications of Percutaneous Coronary Intervention in Elderly Patients. *Cardiol Res*, *8*(5), 199-205. <https://doi.org/10.14740/cr582e>

Hermans, M. P. J., Eindhoven, D. C., van Winden, L. A. M., de Grooth, G. J., Blauw, G. J., Muller, M., & Schalij, M. J. (2019). Frailty score for elderly patients is associated with short-term clinical outcomes in patients with ST-segment elevated myocardial infarction treated with primary percutaneous coronary intervention. *Neth Heart J*, *27*(3), 127-133. <https://doi.org/10.1007/s12471-019-1240-7>

Hillegass, W. B., Patel, M. R., Klein, L. W., Gurm, H. S., Brennan, J. M., Anstrom, K. J., Dai, D., Eisenstein, E. L., Peterson, E. D., Messenger, J. C., & Douglas, P. S. (2012). Long-term outcomes of older diabetic patients after percutaneous coronary stenting in the United States: a report from the National Cardiovascular Data Registry, 2004 to 2008. *J Am Coll Cardiol*, *60*(22), 2280-2289. <https://doi.org/10.1016/j.jacc.2012.08.993>

Horikoshi, T., Nakamura, T., Yoshizaki, T., Nakamura, J., Watanabe, Y., Uematsu, M., Makino, A., Kobayashi, T., Saito, Y., Obata, J. E., Sawanobori, T., Takano, H., Umetani, K., Watanabe, A., Asakawa, T., & Sato, A. (2023). A Stratified Analysis of the Risk Associated With Low Body Mass Index for Patients After Percutaneous Coronary Intervention. *J Atheroscler Thromb*, *30*(5), 502-514. <https://doi.org/10.5551/jat.63650>

Iwashita, M., Matsushita, Y., Sasaki, J., Arakawa, K., & Kono, S. (2004). Relation of serum total cholesterol and other risk factors to risk of coronary events in middle-aged and elderly Japanese men with hypercholesterolemia: the Kyushu Lipid Intervention Study. *Circ J*, *68*(5), 405-409. <https://doi.org/10.1253/circj.68.405>

Jan, M. W., Ali, A., Shah, A. A., Soomro, T. H., Zaheer, A. B., & Sakthivel, S. (2023). IMPACT OF ACUTE KIDNEY INJURY IN ELDERLY (≥80 YEARS) PATIENTS UNDERGOING PERCUTANEOUS CORONARY INTERVENTION [Article]. *Journal of Population Therapeutics and Clinical Pharmacology*, *30*(18), 1106-1112. <https://doi.org/10.53555/jptcp.v30i18.3243>

Jeong, C., Kim, B., Kim, J., Baek, H., Kim, M. K., Sohn, T. S., Baek, K. H., Song, K. H., Son, H. S., Han, K., & Kwon, H. S. (2023). Optimal LDL cholesterol levels in young and old patients with type 2 diabetes for secondary prevention of cardiovascular diseases are different. *Endocr Connect*, *12*(11). <https://doi.org/10.1530/ec-23-0142>

Jin, C., Xu, Y., Qiao, S. B., Tang, X. R., Wu, Y. J., Yan, H. B., Dou, K. F., Xu, B., Yang, J. G., & Yang, Y. J. (2017). Transradial Versus Transfemoral Approach for Percutaneous Coronary Intervention in Elderly Patients in China: A Retrospective Analysis. *Chin Med Sci J*, *32*(3), 161-170. <https://doi.org/10.24920/j1001-9294.2017.023>

Jinnouchi, H., Sakakura, K., Wada, H., Kubo, N., Sugawara, Y., Nakamura, T., Funayama, H., Ako, J., & Momomura, S. (2012). Transradial percutaneous coronary intervention for acute myocardial infarction reduces CCU stay in patients 80 or older. *Int Heart J*, *53*(2), 79-84. <https://doi.org/10.1536/ihj.53.79>

Johnman, C., Oldroyd, K. G., Mackay, D. F., Slack, R., Pell, A. C., Flapan, A. D., Jennings, K. P., Eteiba, H., Irving, J., & Pell, J. P. (2010). Percutaneous coronary intervention in the elderly: changes in case-mix and periprocedural outcomes in 31,758 patients treated between 2000 and 2007. *Circ Cardiovasc Interv*, *3*(4), 341-345. <https://doi.org/10.1161/circinterventions.109.928705>

Jomaa, W., Hamdi, S., Ben Ali, I., Azaiez, M. A., El Hraiech, A., Ben Hamda, K., & Maatouk, F. (2016). Risk profile and in-hospital prognosis in elderly patients presenting for acute ST-elevation myocardial infarction in the Tunisian context. *Indian Heart J*, *68*(6), 760-765. <https://doi.org/10.1016/j.ihj.2016.01.020>

Joshi, F. R., Lønborg, J., Sadjadieh, G., Helqvist, S., Holmvang, L., Sørensen, R., Jørgensen, E., Pedersen, F., Tilsted, H. H., Høfsten, D., Køber, L., Kelbaek, H., & Engstrøm, T. (2021). The benefit of complete revascularization after primary PCI for STEMI is attenuated by increasing age: Results from the DANAMI-3-PRIMULTI randomized study. *Catheter Cardiovasc Interv*, *97*(4), E467-e474. <https://doi.org/10.1002/ccd.29131>

Kabbani, Z., Garcia-Nielsen, L., Martinez, E., Febles, T., & Castro, A. (2007). Percutaneous coronary intervention in the elderly for myocardial infarction in the poststent era. *Crit Pathw Cardiol*, *6*(4), 180-182. <https://doi.org/10.1097/HPC.0b013e31815991e8>

Kaneko, H., Yajima, J., Oikawa, Y., Tanaka, S., Fukamachi, D., Suzuki, S., Sagara, K., Otsuka, T., Matsuno, S., Funada, R., Kano, H., Uejima, T., Koike, A., Nagashima, K., Kirigaya, H., Sawada, H., Aizawa, T., & Yamashita, T. (2014). Impact of aging on the clinical outcomes of Japanese patients with coronary artery disease after percutaneous coronary intervention. *Heart Vessels*, *29*(2), 156-164. <https://doi.org/10.1007/s00380-013-0339-9>

Kang, S. H., Ahn, J. M., Lee, C. H., Lee, P. H., Kang, S. J., Lee, S. W., Kim, Y. H., Lee, C. W., Park, S. W., Park, D. W., & Park, S. J. (2017). Differential Event Rates and Independent Predictors of Long-Term Major Cardiovascular Events and Death in 5795 Patients With Unprotected Left Main Coronary Artery Disease Treated With Stents, Bypass Surgery, or Medication: Insights From a Large International Multicenter Registry. *Circ Cardiovasc Interv*, *10*(7). <https://doi.org/10.1161/circinterventions.116.004988>

Kedhi, E., Verdoia, M., Suryapranata, H., Damen, S., Camaro, C., Benit, E., Barbieri, L., Rasoul, S., Liew, H. B., Polad, J., Ahmad, W. A., Zambahari, R., Lalmand, J., van der Schaaf, R. J., Koh, T. H., Timmermans, P., Sr., Dilling-Boer, D., Veenstra, L. F., Van, T. H. A. W., . . . De Luca, G. (2021). Impact of age on the comparison between short-term vs 12-month dual antiplatelet therapy in patients with acute coronary syndrome treated with the COMBO dual therapy stent: 2-Year follow-up results of the REDUCE trial. *Atherosclerosis*, *321*, 39-44. <https://doi.org/10.1016/j.atherosclerosis.2021.02.006>

Khera, S., Kolte, D., Palaniswamy, C., Mujib, M., Aronow, W. S., Singh, T., Gotsis, W., Silverman, G., & Frishman, W. H. (2013). ST-elevation myocardial infarction in the elderly--temporal trends in incidence, utilization of percutaneous coronary intervention and outcomes in the United States. *Int J Cardiol*, *168*(4), 3683-3690. <https://doi.org/10.1016/j.ijcard.2013.06.021>

Kiatchoosakun, S., Keelapang, P., Kaewsuwana, P., Chotinaiwattarakul, C., & Piumsomboon, C. (2010). Percutaneous coronary intervention in the elderly: results from the Thai National Percutaneous Coronary Intervention Registry (TPCIR). *EuroIntervention*, *6*(5), 611-615. <https://doi.org/10.4244/eijv6i5a102>

Kiliç, O., Özpamuk Karadeniz, F., & Kahraman, F. (2023). THE RELATIONSHIP OF THE C-REACTIVE PROTEIN /ALBUMIN RATIO TO IN-HOSPITAL MORTALITY IN ELDERLY PATIENTS WITH NONST-ELEVATION MYOCARDIAL INFARCTION WHO HAVE UNDERGONE PERCUTANEOUS CORONARY INTERVENTION [Article]. *Turk Geriatri Dergisi*, *26*(1), 12-19. <https://doi.org/10.29400/tjgeri.2023.326>

Kim, D. W., Her, S. H., Park, H. W., Chang, K., Chung, W. S., Seung, K. B., Jeong, M. H., Kim, H. S., Gwon, H. C., Seong, I. W., Hwang, K. K., Chae, S. C., Kim, K. B., Kim, Y. J., Cha, K. S., Oh, S. K., Chae, J. K., & Jung, J. H. (2018). Incremental age-related one-year MACCE after acute myocardial infarction in the drug-eluting stent era (from KAMIR-NIH registry). *J Geriatr Cardiol*, *15*(9), 574-584. <https://doi.org/10.11909/j.issn.1671-5411.2018.09.005>

Kim, J. Y., Jeong, M. H., Choi, Y. W., Ahn, Y. K., Chae, S. C., Hur, S. H., Hong, T. J., Kim, Y. J., Seong, I. W., Chae, I. H., Cho, M. C., Yoon, J. H., & Seung, K. B. (2015). Temporal trends and in-hospital outcomes of primary percutaneous coronary intervention in nonagenarians with ST-segment elevation myocardial infarction. *Korean J Intern Med*, *30*(6), 821-828. <https://doi.org/10.3904/kjim.2015.30.6.821>

Kim, Y. H., Her, A. Y., Kim, B. K., Shin, D. H., Kim, J. S., Ko, Y. G., Choi, D., Hong, M. K., & Jang, Y. (2017). Previous cerebrovascular disease is an important predictor of clinical outcomes in elderly patients with percutaneous coronary interventions: The Nobori-Biolimus eluting stent prospective multicenter 1-year observational registry in South Korea. *Anatol J Cardiol*, *18*(2), 128-135. <https://doi.org/10.14744/AnatolJCardiol.2017.7670>

Kim, Y. H., Her, A. Y., Rha, S. W., Choi, C. U., Choi, B. G., Kim, J. B., Park, S., Kang, D. O., Park, J. Y., Park, S. H., & Jeong, M. H. (2022). Comparison of 3-Year Outcomes between Early and Delayed Invasive Strategies in Older and Younger Adults with Non-ST-Segment Elevation Myocardial Infarction Undergoing New-Generation Drug-Eluting Stent Implantation. *J Clin Med*, *11*(16). <https://doi.org/10.3390/jcm11164780>

Koutouzis, M., Matejka, G., Olivecrona, G., Grip, L., & Albertsson, P. (2010). Radial vs. femoral approach for primary percutaneous coronary intervention in octogenarians. *Cardiovasc Revasc Med*, *11*(2), 79-83. <https://doi.org/10.1016/j.carrev.2009.04.107>

Krivosheeva, E. N., Panchenko, E. P., Kropacheva, E. S., Dobrovolsky, A. B., Titaeva, E. V., Mironov, V. M., & Samko, A. N. (2020). Prediction-Determining Outcomes and Their Predictors in Atrial Fibrillation Patients Receiving Multicomponent Antithrombotic Therapy in Real Clinical Practice. *Kardiologiia*, *60*(8), 33-45. <https://doi.org/10.18087/cardio.2020.8.n1123>

Kuch, B., Wende, R., Barac, M., von Scheidt, W., Kling, B., Greschik, C., & Meisinger, C. (2011). Prognosis and outcomes of elderly (75-84 years) patients with acute myocardial infarction 1-2 years after the event: AMI-elderly study of the MONICA/KORA Myocardial Infarction Registry. *Int J Cardiol*, *149*(2), 205-210. <https://doi.org/10.1016/j.ijcard.2010.01.010>

Kukreja, N., Onuma, Y., Garcia-Garcia, H., van Nierop, J., Daemen, J., van Domburg, R., & Serruys, P. W. (2011). Three-year clinical event rates in different age groups after contemporary percutaneous coronary intervention. *EuroIntervention*, *7*(8), 969-976. <https://doi.org/10.4244/eijv7i8a153>

Kumar, R., O'Connor, C., Kumar, J., Kerr, B., Malik, I., Homer, C., Abbas, S., Arnous, S., Ullah, I., & Kiernan, T. J. (2021). Activation of PPCI team in the octogenarian and nonagenarians population: real-world single-centre experience. *Open Heart*, *8*(2). <https://doi.org/10.1136/openhrt-2021-001709>

Kurobe, M., Uchida, Y., Ishii, H., Yamashita, D., Yonekawa, J., Satake, A., Makino, Y., Hiramatsu, T., Mizutani, K., Mizutani, Y., Ichimiya, H., Amano, T., Watanabe, J., Kanashiro, M., Matsubara, T., Ichimiya, S., & Murohara, T. (2021). Impact of the clinical frailty scale on clinical outcomes and bleeding events in patients with ST-segment elevation myocardial infarction. *Heart Vessels*, *36*(6), 799-808. <https://doi.org/10.1007/s00380-020-01764-0>

Lafont, A., Sinnaeve, P. R., Cuisset, T., Cook, S., Sideris, G., Kedev, S., Carrie, D., Hovasse, T., Garot, P., El Mahmoud, R., Spaulding, C., Helft, G., Diaz Fernandez, J. F., Brugaletta, S., Pinar-Bermudez, E., Ferre, J. M., Commeau, P., Teiger, E., Bogaerts, K., . . . Varenne, O. (2021). Two-year outcomes after percutaneous coronary intervention with drug-eluting stents or bare-metal stents in elderly patients with coronary artery disease. *Catheter Cardiovasc Interv*, *97*(5), E607-e613. <https://doi.org/10.1002/ccd.29159>

Lang, J., Wang, C., Zhang, J., Hu, Y., Wang, L., Liu, Y., Xu, R., Wu, J., Qi, W., Liu, C., Li, W., Li, T., Jin, D., Wei, A., Wang, L., & Cong, H. (2023). Early versus late delayed percutaneous coronary intervention in elderly patients with ST-segment elevation myocardial infarction. *Aging Clin Exp Res*, *35*(6), 1317-1324. <https://doi.org/10.1007/s40520-023-02417-8>

Lee, O. H., Kim, Y., Son, N. H., Cho, D. K., Kim, J. S., Kim, B. K., Choi, D., Hong, M. K., Jeong, M. H., & Jang, Y. (2022). Safety and Efficacy of Contemporary Drug-Eluting Stents in Patients With ST-Segment Elevation Myocardial Infarction and a High Ischemic Risk. *Front Cardiovasc Med*, *9*, 880351. <https://doi.org/10.3389/fcvm.2022.880351>

Lee, S., Miller, R., Lee, M., White, H., & Kerr, A. (2020). Outcomes after ST-elevation myocardial infarction presentation to hospitals with or without a routine primary percutaneous coronary intervention service (ANZACS-QI 46). *N Z Med J*, *133*(1524), 64-81.

Leistner, D. M., Münch, C., Steiner, J., Erbay, A., Riedel, M., Gebhard, C., Lauten, A., Landmesser, U., & Stähli, B. E. (2018). Impact of acute kidney injury in elderly (≥80 years) patients undergoing percutaneous coronary intervention. *J Interv Cardiol*, *31*(6), 792-798. <https://doi.org/10.1111/joic.12547>

Leistner, D. M., Münch, C., Steiner, J., Jakob, P., Reinthaler, M., Sinning, D., Fröhlich, G. M., Mochmann, H. C., Rauch-Kröhnert, U., Skurk, C., Lauten, A., Landmesser, U., & Stähli, B. E. (2018). Effect of Physical Disability on Mortality in Elderly Patients of ≥80 Years of Age Undergoing Percutaneous Coronary Intervention. *Am J Cardiol*, *122*(4), 537-541. <https://doi.org/10.1016/j.amjcard.2018.04.055>

Li, Z. Z., Wu, X. Y., Tao, Y., Wang, S., Yin, C. Q., Gao, Y. L., Cheng, Y. T., Li, Z., & Ma, C. S. (2020). Revascularization versus drug therapy for coronary artery disease in patients aged over 80 years: a real-world study. *Cardiovasc Diagn Ther*, *10*(3), 512-519. <https://doi.org/10.21037/cdt-20-185>

Lim, H. S., Andrianopoulos, N., Sugumar, H., Stub, D., Brennan, A. L., Lim, C. C. S., Barlis, P., Van Gaal, W., Reid, C. M., Charter, K., Sebastian, M., New, G., Ajani, A. E., Farouque, O., Duffy, S. J., & Clark, D. J. (2015). Long-term survival of elderly patients undergoing percutaneous coronary intervention for myocardial infarction complicated by cardiogenic shock [Article]. *International Journal of Cardiology*, *195*, 259-264. <https://doi.org/10.1016/j.ijcard.2015.05.130>

Lim, H. S., Farouque, O., Andrianopoulos, N., Yan, B. P., Lim, C. C., Brennan, A. L., Reid, C. M., Freeman, M., Charter, K., Black, A., New, G., Ajani, A. E., Duffy, S. J., & Clark, D. J. (2009). Survival of elderly patients undergoing percutaneous coronary intervention for acute myocardial infarction complicated by cardiogenic shock. *JACC Cardiovasc Interv*, *2*(2), 146-152. <https://doi.org/10.1016/j.jcin.2008.11.006>

Liu, X. M., Ma, C. S., Liu, X. H., Du, X., Kang, J. P., Zhang, Y., & Wu, J. H. (2015). Relationship between red blood cell distribution width and intermediate-term mortality in elderly patients after percutaneous coronary intervention. *J Geriatr Cardiol*, *12*(1), 17-22. <https://doi.org/10.11909/j.issn.1671-5411.2015.01.013>

Liu, Y., Liu, Y. H., Chen, J. Y., Tan, N., Zhou, Y. L., Duan, C. Y., Yu, D. Q., Xie, N. J., Li, H. L., & Chen, P. Y. (2015). Safe contrast volumes for preventing contrast-induced nephropathy in elderly patients with relatively normal renal function during percutaneous coronary intervention. *Medicine (Baltimore)*, *94*(12), e615. <https://doi.org/10.1097/md.0000000000000615>

Liu, Y., Zhang, C., Jiang, L., Xu, L., Tian, J., Zhao, X., Feng, X., Wang, D., Zhang, Y., Sun, K., Xu, J., Liu, R., Xu, B., Zhao, W., Hui, R., Gao, R., Gao, Z., Song, L., & Yuan, J. (2022). Relationship Between High-Sensitivity C-Reactive Protein and Long-Term Outcomes in Elderly Patients With 3-Vessel Disease. *Angiology*, *73*(1), 60-67. <https://doi.org/10.1177/00033197211021195>

Lu, Y. Y., Lee, C. H., Chen, C. C., Chen, D. Y., Ho, M. Y., Yeh, J. K., Huang, Y. C., Chang, C. Y., Wang, C. Y., Chang, S. H., Hsieh, I. C., & Hsieh, M. J. (2023). Comparison of long-term outcomes of complete vs. incomplete revascularization in elderly patients (≥75 years) with acute coronary syndrome and multi-vessel disease undergoing percutaneous coronary intervention. *Front Cardiovasc Med*, *10*, 1037392. <https://doi.org/10.3389/fcvm.2023.1037392>

Maekawa, Y., Kawamura, A., Yuasa, S., Ohno, Y., Arai, T., Numasawa, Y., Endo, A., & Fukuda, K. (2011). Outcomes of intravascular ultrasound-guided percutaneous coronary intervention with drug-eluting stents versus bare metal stents for acute coronary syndrome in octogenarians. *Angiology*, *62*(8), 620-624. <https://doi.org/10.1177/0003319711403733>

Mahabaleshwarkar, R. K., Yang, Y., Datar, M. V., Bentley, J. P., Strum, M. W., Banahan, B. F., & Null, K. D. (2013). Risk of adverse cardiovascular outcomes and all-cause mortality associated with concomitant use of clopidogrel and proton pump inhibitors in elderly patients. *Curr Med Res Opin*, *29*(4), 315-323. <https://doi.org/10.1185/03007995.2013.772051>

Mahadevappa, M., Desai, N., Kumar, S. S., & Kulkarni, P. (2020). Primary percutaneous coronary intervention in elderly patients with acute myocardial infarction: A single centre experience from Southern India [Article]. *Journal of Clinical and Diagnostic Research*, *14*(4), OC16-OC20. <https://doi.org/10.7860/JCDR/2020/44116.13642>

Malpica, E. M., Duque, M. A. P., Castellanos, J., Exaire, E., Arrieta, O., Dávila, E. S., Fernández, R. V., Delgadillo-Rodríguez, H., González-Quesada, C. J., & Martínez-Ríos, M. A. (2007). Predictors of mortality and adverse outcome in elderly high-risk patients undergoing percutaneous coronary intervention [Article]. *Archivos de Cardiologia de Mexico*, *77*(3), 194-199. <https://www.embase.com/search/results?subaction=viewrecord&id=L47584512&from=export>

Mangalesh, S., Daniel, K. V., Dudani, S., & Joshi, A. (2023). Combined nutritional and frailty screening improves assessment of short-term prognosis in older adults following percutaneous coronary intervention. *Coron Artery Dis*, *34*(3), 185-194. <https://doi.org/10.1097/mca.0000000000001221>

Marcolino, M. S., Simsek, C., de Boer, S. P., van Domburg, R. T., van Geuns, R. J., de Jaegere, P., Akkerhuis, K. M., Daemen, J., Serruys, P. W., & Boersma, E. (2012). Short- and long-term outcomes in octogenarians undergoing percutaneous coronary intervention with stenting. *EuroIntervention*, *8*(8), 920-928. <https://doi.org/10.4244/eijv8i8a141>

McGrath, P. D., Wennberg, D. E., Dickens, J. D., Jr., Siewers, A. E., Lucas, F. L., Malenka, D. J., Kellett, M. A., Jr., & Ryan, T. J., Jr. (2000). Relation between operator and hospital volume and outcomes following percutaneous coronary interventions in the era of the coronary stent. *Jama*, *284*(24), 3139-3144. <https://doi.org/10.1001/jama.284.24.3139>

Medina, H. M., Cannon, C. P., Fonarow, G. C., Grau-Sepulveda, M. V., Hernandez, A. F., Frank Peacock, W., Laskey, W., Peterson, E. D., Schwamm, L., & Bhatt, D. L. (2012). Reperfusion strategies and quality of care in 5339 patients age 80 years or older presenting with ST-elevation myocardial infarction: analysis from get with the guidelines-coronary artery disease. *Clin Cardiol*, *35*(10), 632-640. <https://doi.org/10.1002/clc.22036>

Mehta, R. H., Sadiq, I., Goldberg, R. J., Gore, J. M., Avezum, A., Spencer, F., Kline-Rogers, E., Allegrone, J., Pieper, K., Fox, K. A., & Eagle, K. A. (2004). Effectiveness of primary percutaneous coronary intervention compared with that of thrombolytic therapy in elderly patients with acute myocardial infarction. *Am Heart J*, *147*(2), 253-259. <https://doi.org/10.1016/j.ahj.2003.08.007>

Miura, T., Miyashita, Y., Motoki, H., Shimada, K., Kobayashi, M., Nakajima, H., Kimura, H., Akanuma, H., Mawatari, E., Sato, T., Hotta, S., Kamiyoshi, Y., Maruyama, T., Watanabe, N., Eisawa, T., Aso, S., Uchikawa, S., Hashizume, N., Sekimura, N., . . . Ikeda, U. (2014). In-hospital clinical outcomes of elderly patients (≥80 years) undergoing percutaneous coronary intervention. *Circ J*, *78*(5), 1097-1103. <https://doi.org/10.1253/circj.cj-14-0129>

Moon, I. T., Kang, S. H., Lee, W., Cho, Y., Park, J. J., Yoon, Y. E., Oh, I. Y., Yoon, C. H., Suh, J. W., Youn, T. J., Chae, I. H., Choi, D. J., & Cho, Y. S. (2021). Impact of statin intensity on adverse cardiac and cerebrovascular events in older adult patients with myocardial infarction. *J Geriatr Cardiol*, *18*(8), 609-622. <https://doi.org/10.11909/j.issn.1671-5411.2021.08.005>

Moon, J., Suh, J., Oh, P. C., Lee, K., Park, H. W., Jang, H. J., Kim, T. H., Park, S. D., Kwon, S. W., & Kang, W. C. (2016). Relation of Stature to Outcomes in Korean Patients Undergoing Primary Percutaneous Coronary Intervention for Acute ST-Elevation Myocardial Infarction (from the INTERSTELLAR Registry). *Am J Cardiol*, *118*(2), 177-182. <https://doi.org/10.1016/j.amjcard.2016.04.046>

Morici, N., De Servi, S., Toso, A., Murena, E., Piscione, F., Bolognese, L., Petronio, A. S., Antonicelli, R., Cavallini, C., Angeli, F., & Savonitto, S. (2015). Renal dysfunction, coronary revascularization and mortality among elderly patients with non ST elevation acute coronary syndrome. *Eur Heart J Acute Cardiovasc Care*, *4*(5), 453-460. <https://doi.org/10.1177/2048872614557221>

Murphy, J. C., Kozor, R. A., Figtree, G., Hansen, P. S., Rasmussen, H. H., Ward, M. R., Nelson, G. I., & Bhindi, R. (2012). Procedural and in-patient outcomes in patients aged 80 years or older undergoing contemporary primary percutaneous coronary intervention. *EuroIntervention*, *8*(8), 912-919. <https://doi.org/10.4244/eijv8i8a140>

Nakamura, M., Iizuka, T., Sagawa, K., Abe, K., Chikada, S., & Arai, M. (2018). Prasugrel for Japanese patients with acute coronary syndrome in short-term clinical practice (PRASFIT-Practice I): a postmarketing observational study. *Cardiovasc Interv Ther*, *33*(2), 135-145. <https://doi.org/10.1007/s12928-017-0459-8>

Nakamura, T., Horikoshi, T., Kobayahi, T., Yoshizaki, T., Uematsu, M., Watanabe, Y., Nakamura, J., Makino, A., Saito, Y., Obata, J. E., Sawanobori, T., Takano, H., Umetani, K., Watanabe, A., Asakawa, T., & Sato, A. (2023). Optimal medical therapy after percutaneous coronary intervention in very elderly patients with coronary artery disease. *Int J Cardiol Cardiovasc Risk Prev*, *16*, 200162. <https://doi.org/10.1016/j.ijcrp.2022.200162>

Nammas, W., de Belder, A., Niemelä, M., Sia, J., Romppanen, H., Laine, M., & Karjalainen, P. P. (2017). Long-term clinical outcome of elderly patients with acute coronary syndrome treated with early percutaneous coronary intervention: Insights from the BASE ACS randomized controlled trial: Bioactive versus everolimus-eluting stents in elderly patients. *Eur J Intern Med*, *37*, 43-48. <https://doi.org/10.1016/j.ejim.2016.07.027>

Nguyen, T. V., Bui, K. X., Tran, K. D., Le, D., & Nguyen, T. N. (2020). Non-ST elevation acute coronary syndrome in patients aged 80 years or older in Vietnam: An observational study. *PLoS ONE*, *15*(6), e0233272. <https://doi.org/10.1371/journal.pone.0233272>

Nishihira, K., Yoshioka, G., Kuriyama, N., Ogata, K., Kimura, T., Matsuura, H., Furugen, M., Koiwaya, H., Watanabe, N., & Shibata, Y. (2021). Impact of frailty on outcomes in elderly patients with acute myocardial infarction who undergo percutaneous coronary intervention. *Eur Heart J Qual Care Clin Outcomes*, *7*(2), 189-197. <https://doi.org/10.1093/ehjqcco/qcaa018>

Nowak, Z., Plewa, M., Skowron, M., Markiewicz, A., Kucio, C., & Osiadło, G. (2010). Paffenbarger Physical Activity Questionnaire as an additional tool in clinical assessment of patients with coronary artery disease treated with angioplasty. *Kardiol Pol*, *68*(1), 32-39.

Oh, S., Jeong, M. H., Cho, K. H., Kim, M. C., Sim, D. S., Hong, Y. J., Kim, J. H., & Ahn, Y. (2022). Outcomes of Nonagenarians with Acute Myocardial Infarction with or without Coronary Intervention. *J Clin Med*, *11*(6). <https://doi.org/10.3390/jcm11061593>

Ono, M., Serruys, P. W., Hara, H., Kawashima, H., Gao, C., Wang, R., Takahashi, K., O'Leary, N., Wykrzykowska, J. J., Sharif, F., Piek, J. J., Garg, S., Mack, M. J., Holmes, D. R., Morice, M. C., Head, S. J., Kappetein, A. P., Thuijs, D., Noack, T., . . . Onuma, Y. (2021). 10-Year Follow-Up After Revascularization in Elderly Patients With Complex Coronary Artery Disease. *J Am Coll Cardiol*, *77*(22), 2761-2773. <https://doi.org/10.1016/j.jacc.2021.04.016>

Oqueli, E., & Dick, R. (2011). Percutaneous coronary intervention in very elderly patients. In-hospital mortality and clinical outcome. *Heart Lung Circ*, *20*(10), 622-628. <https://doi.org/10.1016/j.hlc.2010.08.012>

Park, D. Y., Jamil, Y., Hu, J. R., Lowenstern, A., Frampton, J., Abdullah, A., Damluji, A. A., Ahmad, Y., Soufer, R., & Nanna, M. G. (2023). Delirium in older adults after percutaneous coronary intervention: Prevalence, risks, and clinical phenotypes. *Cardiovasc Revasc Med*. <https://doi.org/10.1016/j.carrev.2023.06.010>

Park, H., Ahn, J. M., Yoon, Y. H., Kwon, O., Lee, K., Kang, D. Y., Lee, P. H., Lee, S. W., Park, S. W., Park, D. W., & Park, S. J. (2019). Effect of Age and Sex on Outcomes After Stenting or Bypass Surgery in Left Main Coronary Artery Disease. *Am J Cardiol*, *124*(5), 678-687. <https://doi.org/10.1016/j.amjcard.2019.05.061>

Phan, D. Q., Zadegan, R., & Lee, M. S. (2021). Revascularization versus medical therapy in patients aged 80 and older with stable ischemic heart disease. *J Am Geriatr Soc*, *69*(12), 3457-3467. <https://doi.org/10.1111/jgs.17404>

Poludasu, S., Cavusoglu, E., Khan, W., & Marmur, J. D. (2009). Neutrophil to lymphocyte ratio as a predictor of long-term mortality in African Americans undergoing percutaneous coronary intervention [Article]. *Clinical Cardiology*, *32*(12), E6-E10. <https://doi.org/10.1002/clc.20503>

Poorhosseini, H., Mousavi, M., Nematipour, E., Kassaian, S. E., Salarifar, M., Alidoosti, M., Hajizeinali, A., Nozari, Y., Amirzadegan, A., Hosseini, S. K., & Sheikhfathollahi, M. (2011). Success rate, procedural complications and clinical outcomes of coronary interventions in octogenarians: a case-control study. *J Tehran Heart Cent*, *6*(3), 126-133. <https://www.ncbi.nlm.nih.gov/pmc/articles/PMC3466891/pdf/jthc-6-126.pdf>

Prasad, A., Lennon, R. J., Rihal, C. S., Berger, P. B., & Holmes, D. R., Jr. (2004). Outcomes of elderly patients with cardiogenic shock treated with early percutaneous revascularization. *Am Heart J*, *147*(6), 1066-1070. <https://doi.org/10.1016/j.ahj.2003.07.030>

Puymirat, E., Mangiacapra, F., Peace, A., Ntarladimas, Y., Conte, M., Bartunek, J., Vanderheyden, M., Wijns, W., de Bruyne, B., & Barbato, E. (2013). Safety and effectiveness of drug-eluting stents versus bare-metal stents in elderly patients with small coronary vessel disease. *Arch Cardiovasc Dis*, *106*(11), 554-561. <https://doi.org/10.1016/j.acvd.2013.06.056>

Qaderdan, K., Vos, G. A., McAndrew, T., Steg, P. G., Hamm, C. W., Van't Hof, A., Mehran, R., Deliargyris, E. N., Bernstein, D., Stone, G. W., & Ten Berg, J. M. (2017). Outcomes in elderly and young patients with ST-segment elevation myocardial infarction undergoing primary percutaneous coronary intervention with bivalirudin versus heparin: Pooled analysis from the EUROMAX and HORIZONS-AMI trials. *Am Heart J*, *194*, 73-82. <https://doi.org/10.1016/j.ahj.2017.08.009>

Ramakrishna, A. S., Rao, V. S. K., & Indrani, G. (2020). Gender-wise Long-term Predictors for Major Adverse Cardiac Events following Percutaneous Coronary Intervention in the Elderly Population [Article]. *Indian Journal of Cardiovascular Disease in Women - WINCARS*, *5*(1), 18-24. <https://doi.org/10.1055/s-0040-1709921>

Räsänen, A., Kärkkäinen, J. M., Eranti, A., Eränen, J., & Rissanen, T. T. (2023). Percutaneous coronary intervention with drug-coated balloon-only strategy combined with single antiplatelet treatment in patients at high bleeding risk: Single center experience of a novel concept. *Catheter Cardiovasc Interv*, *101*(3), 569-578. <https://doi.org/10.1002/ccd.30558>

Rencuzogullari, I., Çağdaş, M., Karabağ, Y., Karakoyun, S., Yesin, M., Çinar, T., Tanik, V. O., Burak, C., & Tanboğa İ, H. (2019). Value of syntax score II for predicting in-hospital and long-term survival in octogenarians with ST-segment elevation myocardial infarction: A comparison of six different risk scores. *Arch Gerontol Geriatr*, *83*, 37-43. <https://doi.org/10.1016/j.archger.2019.03.016>

Rittger, H., Hochadel, M., Behrens, S., Hauptmann, K. E., Zahn, R., Mudra, H., Brachmann, J., & Zeymer, U. (2014). Interventional treatment and outcome in elderly patients with stable coronary artery disease. Results from the German ALKK registry. *Herz*, *39*(2), 212-218. <https://doi.org/10.1007/s00059-013-3822-2>

Rodriguez-Leor, O., Fernandez-Nofrerias, E., Carrillo, X., Mauri, J., Labata, C., Oliete, C., Rivas Mdel, C., & Bayes-Genis, A. (2014). Results of primary percutaneous coronary intervention in patients ≥75 years treated by the transradial approach. *Am J Cardiol*, *113*(3), 452-456. <https://doi.org/10.1016/j.amjcard.2013.10.030>

Roubelakis, A., Casselman, F., van der Merwe, J., Stockman, B., Degrieck, I., & Van Praet, F. (2017). Robotic-enhanced coronary surgery in octogenarians. *Interact Cardiovasc Thorac Surg*, *24*(3), 384-387. <https://doi.org/10.1093/icvts/ivw369>

Rozenfeld, K. L., Lupu, L., Merdler, I., Morgan, S., Banai, S., & Shacham, Y. (2022). Invasive versus Conservative Treatment Approach among Older Adult Patients Admitted with Acute ST-Segment Elevation Myocardial Infarction. *Ann Geriatr Med Res*, *26*(4), 347-353. <https://doi.org/10.4235/agmr.22.0079>

Rumiz, E., Berenguer, A., Vilar, J. V., Valero, E., Facila, L., Cubillos, A., Sanmiguel, D., Almela, P., & Morell, S. (2018). Long-term outcomes and predictors of morbi-mortality according to age in stemi patients with multivessel disease: Impact of an incomplete revascularization. *Catheter Cardiovasc Interv*, *92*(7), E512-e517. <https://doi.org/10.1002/ccd.27691>

Rymuza, H., Kowalik, I., Drzewiecki, A., Krzyżanowski, W., Olszewski, M., Dąbrowski, R., Jędrzejczyk, B., Woźniak, J., Sosnowski, C., & Szwed, H. (2011). Successful primary coronary angioplasty improves early and long-term outcomes in ST segment elevation acute coronary syndromes in patients above 80 years of age. *Kardiol Pol*, *69*(4), 346-354.

Rynkowska-Kidawa, M., Zielińska, M., Chiżyński, K., & Kidawa, M. (2015). In-hospital outcomes and mortality in octogenarians after percutaneous coronary intervention. *Kardiol Pol*, *73*(6), 396-403. <https://doi.org/10.5603/KP.a2014.0247>

Sadeghi, H. M., Grines, C. L., Chandra, H. R., Dixon, S. R., Boura, J. A., Dukkipati, S., Harjai, K. J., & O'Neill, W. W. (2003). Percutaneous coronary interventions in octogenarians. glycoprotein IIb/IIIa receptor inhibitors' safety profile. *J Am Coll Cardiol*, *42*(3), 428-432. <https://doi.org/10.1016/s0735-1097(03)00657-0>

Sanz-Girgas, E., Peiró Ó, M., Bonet, G., Rodríguez-López, J., Scardino, C., Ferrero-Guillem, M., Vásquez, K., Romeu-Nieto, A., & Bardají, A. (2021). A simple combination of biomarkers for risk stratification in octogenarians with acute myocardial infarction. *Rev Cardiovasc Med*, *22*(4), 1711-1720. <https://doi.org/10.31083/j.rcm2204179>

Sappa, R., Grillo, M. T., Cinquetti, M., Prati, G., Spedicato, L., Nucifora, G., Perkan, A., Zanuttini, D., Sinagra, G., & Proclemer, A. (2017). Short and long-term outcome in very old patients with ST-elevation myocardial infarction after primary percutaneous coronary intervention. *Int J Cardiol*, *249*, 112-118. <https://doi.org/10.1016/j.ijcard.2017.09.025>

Sawayama, Y., Yamaji, K., Kohsaka, S., Yamamoto, T., Higo, Y., Numasawa, Y., Inohara, T., Ishii, H., Amano, T., Ikari, Y., & Nakagawa, Y. (2021). Variation in in-hospital mortality and its association with percutaneous coronary intervention-related bleeding complications: A report from nationwide registry in Japan [Article]. *PLoS ONE*, *16*(12 December), Article e0261371. <https://doi.org/10.1371/journal.pone.0261371>

Schmucker, J., Fach, A., Mata Marin, L. A., Retzlaff, T., Osteresch, R., Kollhorst, B., Hambrecht, R., Pohlabeln, H., & Wienbergen, H. (2019). Efficacy and Safety of Ticagrelor in Comparison to Clopidogrel in Elderly Patients With ST-Segment-Elevation Myocardial Infarctions. *J Am Heart Assoc*, *8*(18), e012530. <https://doi.org/10.1161/jaha.119.012530>

Schröder, J., Müller-Werdan, U., Reuter, S., Vogt, A., Schlitt, M., Raaz, U., Reindl, I., Buerke, M., Werdan, K., & Schlitt, A. (2013). Are the elderly different? Factors influencing mortality after percutaneous coronary intervention with stent implantation. *Z Gerontol Geriatr*, *46*(2), 144-150. <https://doi.org/10.1007/s00391-012-0338-y>

Sheridan, B. C., Stearns, S. C., Rossi, J. S., D'Arcy, L. P., Federspiel, J. J., & Carey, T. S. (2010). Three-year outcomes of multivessel revascularization in very elderly acute coronary syndrome patients. *Ann Thorac Surg*, *89*(6), 1889-1894; discussion 1894-1885. <https://doi.org/10.1016/j.athoracsur.2010.03.003>

Shih, J. Y., Chen, Z. C., Chang, H. Y., Liu, Y. W., Ho, C. H., & Chang, W. T. (2019). Risks of age and sex on clinical outcomes post myocardial infarction. *Int J Cardiol Heart Vasc*, *23*, 100350. <https://doi.org/10.1016/j.ijcha.2019.100350>

Shirasawa, K., Hwang, M. W., Sasaki, Y., Takeda, S., Inenaga-Kitaura, K., Kitaura, Y., & Kawai, C. (2011). Survival and changes in physical ability after coronary revascularization for octa-nonagenerian patients with acute coronary syndrome. *Heart Vessels*, *26*(4), 385-391. <https://doi.org/10.1007/s00380-010-0067-3>

Sigurjonsdottir, R., Barywani, S., Albertsson, P., & Fu, M. (2016). Long-term major adverse cardiovascular events and quality of life after coronary angiography in elderly patients with acute coronary syndrome. *Int J Cardiol*, *222*, 481-485. <https://doi.org/10.1016/j.ijcard.2016.07.237>

Sillano, D., Resmini, C., Meliga, E., Boccuzzi, G., Zuffi, A., Barbato, E., Gunn, J., Price, M., Gaita, F., & Sheiban, I. (2013). Retrospective multicenter observational study of the interventional management of coronary disease in the very elderly: the NINETY. *Catheter Cardiovasc Interv*, *82*(3), 414-421. <https://doi.org/10.1002/ccd.24406>

Silva, C., Klein, C. H., Godoy, P. H., Salis, L. H. A., & Silva, N. (2018). Up to 15-Year Survival of Men and Women after Percutaneous Coronary Intervention Paid by the Brazilian Public Healthcare System in the State of Rio de Janeiro, 1999-2010. *Arq Bras Cardiol*, *111*(4), 553-561. <https://doi.org/10.5935/abc.20180184> (Sobrevida em até 15 Anos de Homens e Mulheres após Intervenção Coronariana Percutânea Paga pelo Sistema Único de Saúde no Estado do Rio de Janeiro, 1999-2010.)

Sinning, J. M., Asdonk, T., Erlhöfer, C., Vasa-Nicotera, M., Grube, E., Nickenig, G., & Werner, N. (2013). Combination of angiographic and clinical characteristics for the prediction of clinical outcomes in elderly patients undergoing multivessel PCI. *Clin Res Cardiol*, *102*(12), 865-873. <https://doi.org/10.1007/s00392-013-0599-5>

Sliman, H., Jaffe, R., Rubinshtein, R., Karkabi, B., Zissman, K., Flugelman, M. Y., & Zafrir, B. (2019). Clinical features and outcomes of revascularization in very old patients with left main coronary artery disease. *Coron Artery Dis*, *30*(8), 584-589. <https://doi.org/10.1097/mca.0000000000000744>

Son, Y. J., Shim, S. K., Hwang, S. Y., Ahn, J. H., & Yu, H. Y. (2016). Impact of left ventricular ejection fraction and medication adherence on major adverse cardiac events during the first year after successful primary percutaneous coronary interventions [Article]. *Journal of Clinical Nursing*, *25*(7-8), 1101-1111. <https://doi.org/10.1111/jocn.13109>

Spyridopoulos, I., Noman, A., Ahmed, J. M., Das, R., Edwards, R., Purcell, I., Bagnall, A., Zaman, A., & Egred, M. (2015). Shock-index as a novel predictor of long-term outcome following primary percutaneous coronary intervention. *Eur Heart J Acute Cardiovasc Care*, *4*(3), 270-277. <https://doi.org/10.1177/2048872614561480>

Su, Y. M., Cai, X. X., Geng, H. H., Sheng, H. Z., Fan, M. K., & Pan, M. (2015). In-hospital clinical outcomes of elderly patients (≥60 years) undergoing primary percutaneous coronary intervention. *Int J Clin Exp Med*, *8*(7), 11244-11251. <https://www.ncbi.nlm.nih.gov/pmc/articles/PMC4565314/pdf/ijcem0008-11244.pdf>

Su, Y. M., Pan, M., Geng, H. H., Zhang, R., Qu, Y. Y., & Ma, G. S. (2019). Outcomes after percutaneous coronary intervention and comparison among scoring systems in predicting procedural success in elderly patients (≥ 75 years) with chronic total occlusion. *Coron Artery Dis*, *30*(7), 481-487. <https://doi.org/10.1097/mca.0000000000000765>

Tamez, H., Secemsky, E. A., Valsdottir, L. R., Moussa, I. D., Song, Y., Simonton, C. A., Gibson, C. M., Popma, J. J., & Yeh, R. W. (2021). Long-term outcomes of percutaneous coronary intervention for in-stent restenosis among Medicare beneficiaries. *EuroIntervention*, *17*(5), e380-e387. <https://doi.org/10.4244/eij-d-19-01031>

Tanaka, A., Ishii, H., Tatami, Y., Shibata, Y., Osugi, N., Ota, T., Okumura, S., Suzuki, S., Inoue, Y., & Murohara, T. (2014). Impact of diabetic retinopathy on late cardiac events after percutaneous coronary intervention [Article]. *Journal of Cardiology*, *64*(3), 175-178. <https://doi.org/10.1016/j.jjcc.2013.12.006>

Teplitsky, I., Assali, A., Lev, E., Brosh, D., Vaknin-Assa, H., & Kornowski, R. (2007). Results of percutaneous coronary interventions in patients > or =90 years of age. *Catheter Cardiovasc Interv*, *70*(7), 937-943. <https://doi.org/10.1002/ccd.21263>

Thomas, M. P., Moscucci, M., Smith, D. E., Aronow, H., Share, D., Kraft, P., & Gurm, H. S. (2011). Outcome of contemporary percutaneous coronary intervention in the elderly and the very elderly: insights from the Blue Cross Blue Shield of Michigan Cardiovascular Consortium. *Clin Cardiol*, *34*(9), 549-554. <https://doi.org/10.1002/clc.20926>

Tomaniak, M., Chichareon, P., Modolo, R., Takahashi, K., Chang, C. C., Kogame, N., Spitzer, E., Buszman, P. E., van Geuns, R. M., Valkov, V., Steinwender, C., Geisler, T., Prokopczuk, J., Sabaté, M., Zmudka, K., Rademaker-Havinga, T., Tijssen, J. G. P., Jüni, P., Hamm, C., . . . Serruys, P. W. (2020). Ticagrelor monotherapy beyond one month after PCI in ACS or stable CAD in elderly patients: a pre-specified analysis of the GLOBAL LEADERS trial. *EuroIntervention*, *15*(18), e1605-e1614. <https://doi.org/10.4244/eij-d-19-00699>

Tong, J., Xiang, W. W., Ang, A. S., Sim, W. J., Quah, K. H., Foo, D., Ong, P. J., & Ho, H. H. (2016). Clinical outcomes of elderly South-East Asian patients in primary percutaneous coronary intervention for ST-elevation myocardial infarction. *J Geriatr Cardiol*, *13*(10), 830-835. <https://doi.org/10.11909/j.issn.1671-5411.2016.10.001>

Ueki, Y., Miura, T., Miyashita, Y., Motoki, H., Shimada, K., Kobayashi, M., Nakajima, H., Kimura, H., Akanuma, H., Mawatari, E., Sato, T., Hotta, S., Kamiyoshi, Y., Maruyama, T., Watanabe, N., Eisawa, T., Aso, S., Uchikawa, S., Hashizume, N., . . . Ikeda, U. (2016). Predictive Value of Combining the Ankle-Brachial Index and SYNTAX Score for the Prediction of Outcome After Percutaneous Coronary Intervention (from the SHINANO Registry). *Am J Cardiol*, *117*(2), 179-185. <https://doi.org/10.1016/j.amjcard.2015.10.042>

Valle, J. A., McCoy, L. A., Maddox, T. M., Rumsfeld, J. S., Ho, P. M., Casserly, I. P., Nallamothu, B. K., Roe, M. T., Tsai, T. T., & Messenger, J. C. (2017). Longitudinal Risk of Adverse Events in Patients With Acute Kidney Injury After Percutaneous Coronary Intervention: Insights From the National Cardiovascular Data Registry. *Circ Cardiovasc Interv*, *10*(4). <https://doi.org/10.1161/circinterventions.116.004439>

Van de Werf, F., Ristić, A. D., Averkov, O. V., Arias-Mendoza, A., Lambert, Y., Kerr Saraiva, J. F., Sepulveda, P., Rosell-Ortiz, F., French, J. K., Musić, L. B., Vandenberghe, K., Bogaerts, K., Westerhout, C. M., Pagès, A., Danays, T., Bainey, K. R., Sinnaeve, P., Goldstein, P., Welsh, R. C., & Armstrong, P. W. (2023). STREAM-2: Half-Dose Tenecteplase or Primary Percutaneous Coronary Intervention in Older Patients With ST-Segment-Elevation Myocardial Infarction: A Randomized, Open-Label Trial. *Circulation*, *148*(9), 753-764. <https://doi.org/10.1161/circulationaha.123.064521>

Vandecasteele, E. H., De Buyzere, M., Gevaert, S., de Meester, A., Convens, C., Dubois, P., Boland, J., Sinnaeve, P., De Raedt, H., Vranckx, P., Coussement, P., Evrard, P., Beauloye, C., Renard, M., & Claeys, M. J. (2013). Reperfusion therapy and mortality in octogenarian STEMI patients: results from the Belgian STEMI registry. *Clin Res Cardiol*, *102*(11), 837-845. <https://doi.org/10.1007/s00392-013-0600-3>

Varani, E., Aquilina, M., Balducelli, M., Vecchi, G., Frassineti, V., & Maresta, A. (2009). Percutaneous coronary interventions in octogenarians: Acute and 12 month results in a large single-centre experience. *Catheter Cardiovasc Interv*, *73*(4), 449-454. <https://doi.org/10.1002/ccd.21852>

Varenne, O., Cook, S., Sideris, G., Kedev, S., Cuisset, T., Carrié, D., Hovasse, T., Garot, P., El Mahmoud, R., Spaulding, C., Helft, G., Diaz Fernandez, J. F., Brugaletta, S., Pinar-Bermudez, E., Mauri Ferre, J., Commeau, P., Teiger, E., Bogaerts, K., Sabate, M., . . . Sinnaeve, P. R. (2018). Drug-eluting stents in elderly patients with coronary artery disease (SENIOR): a randomised single-blind trial. *Lancet*, *391*(10115), 41-50. <https://doi.org/10.1016/s0140-6736(17)32713-7>

Velders, M. A., James, S. K., Libungan, B., Sarno, G., Fröbert, O., Carlsson, J., Schalij, M. J., Albertsson, P., & Lagerqvist, B. (2014). Prognosis of elderly patients with ST-elevation myocardial infarction treated with primary percutaneous coronary intervention in 2001 to 2011: A report from the Swedish Coronary Angiography and Angioplasty Registry (SCAAR) registry. *Am Heart J*, *167*(5), 666-673. <https://doi.org/10.1016/j.ahj.2014.01.013>

Wang, J. L., Guo, C. Y., Chen, H., Li, H. W., Zhao, X. Q., & Zhao, S. M. (2021). Improvement of long-term clinical outcomes by successful PCI in the very elderly women with ACS. *BMC Cardiovasc Disord*, *21*(1), 122. <https://doi.org/10.1186/s12872-021-01933-7>

Wang, Q., Tan, Q., Liu, D., Yang, H., & Ma, L. (2014). Clinical outcomes of transradial unprotected left main coronary artery stenting in the elderly. *Saudi Med J*, *35*(8), 838-842.

Wang, T. Y., Masoudi, F. A., Messenger, J. C., Shunk, K. A., Boyle, A., Brennan, J. M., Anderson, H. V., Anstrom, K. J., Dai, D., Peterson, E. D., Douglas, P. S., & Rumsfeld, J. S. (2012). Percutaneous coronary intervention and drug-eluting stent use among patients ≥85 years of age in the United States. *J Am Coll Cardiol*, *59*(2), 105-112. <https://doi.org/10.1016/j.jacc.2011.10.853>

Wang, Y. C., Hwang, J. J., Hung, C. S., Kao, H. L., Chiang, F. T., & Tseng, C. D. (2006). Outcome of primary percutaneous coronary intervention in octogenarians with acute myocardial infarction. *J Formos Med Assoc*, *105*(6), 451-458. <https://doi.org/10.1016/s0929-6646(09)60184-4>

Wenaweser, P., Ramser, M., Windecker, S., Lütolf, I., Meier, B., Seiler, C., Eberli, F. R., & Hess, O. M. (2007). Outcome of elderly patients undergoing primary percutaneous coronary intervention for acute ST-elevation myocardial infarction. *Catheter Cardiovasc Interv*, *70*(4), 485-490. <https://doi.org/10.1002/ccd.21128>

Wiemer, M., Langer, C., Kottmann, T., Horstkotte, D., Hamm, C., Pfannebecker, T. H., Tebbe, U., Schneider, S., & Senges, J. (2007). Outcome in the elderly undergoing percutaneous coronary intervention with sirolimus-eluting stents: results from the prospective multicenter German Cypher Stent Registry. *Am Heart J*, *154*(4), 682-687. <https://doi.org/10.1016/j.ahj.2007.06.019>

Won, H., Her, A. Y., Kim, B. K., Kim, Y. H., Shin, D. H., Kim, J. S., Ko, Y. G., Choi, D., Kwon, H. M., Jang, Y., & Hong, M. K. (2016). Percutaneous Coronary Intervention Is More Beneficial Than Optimal Medical Therapy in Elderly Patients with Angina Pectoris. *Yonsei Med J*, *57*(2), 382-387. <https://doi.org/10.3349/ymj.2016.57.2.382>

Wong, B., Lee, K. H., & El-Jack, S. (2021). Very Elderly Patients With Acute Coronary Syndromes Treated With Percutaneous Coronary Intervention. *Heart Lung Circ*, *30*(9), 1337-1342. <https://doi.org/10.1016/j.hlc.2021.03.275>

Xu, S., Liang, Y., Chen, Y., Gao, H., Tan, Z., Wang, Q., Liu, Y., Zhu, B., Tao, F., Wang, Q., Zhao, S., Yang, L., Zhang, Y., Wang, Z., Han, P., Chen, Y., Zhang, A., Li, C., & Lian, K. (2022). Comparative Effectiveness and Safety of Ticagrelor Versus Clopidogrel for Elderly Chinese Patients Undergoing Percutaneous Coronary Intervention: A Single-Center Retrospective Cohort Study. *Drugs Aging*, *39*(9), 695-703. <https://doi.org/10.1007/s40266-022-00971-w>

Yeh, J. S., Chen, W. T., Tomlinson, B., Tam, W. C., & Chien, L. N. (2023). Comparing the effectiveness and safety of dual antiplatelet with ticagrelor or clopidogrel in elderly Asian patients with acute myocardial infraction. *Front Cardiovasc Med*, *10*, 1143509. <https://doi.org/10.3389/fcvm.2023.1143509>

Zhang, H. P., Ai, H., Zhao, Y., Li, H., Tang, G. D., Zheng, N. X., Sun, F. C., & Liu, J. H. (2018). Effect of Chronic Total Occlusion Percutaneous Coronary Intervention on Clinical Outcomes in Elderly Patients. *Am J Med Sci*, *355*(2), 174-182. <https://doi.org/10.1016/j.amjms.2017.09.007>

Zhang, J. W., Zhou, Y. J., Yang, Q., Yang, S. W., Nie, B., & Xu, X. H. (2013). Impact of chronic obstructive pulmonary diseases on outcomes and hospital days after percutaneous coronary intervention. *Angiology*, *64*(6), 430-434. <https://doi.org/10.1177/0003319712458145>

Zhang, Y., Peng, W., Shi, X., Han, J., Wang, Y., Fang, Z., & Lin, Y. (2022). Ticagrelor vs. Clopidogrel in Older Patients With Acute Coronary Syndrome Undergoing Percutaneous Coronary Intervention: Insights From a Real-World Registry. *Front Cardiovasc Med*, *9*, 859962. <https://doi.org/10.3389/fcvm.2022.859962>

Zhang, Y., Zhai, G., Wang, J., & Zhou, Y. (2022). Risk Factors of Cardiac Death for Elderly Patients with Severe Chronic Kidney Disease after Percutaneous Coronary Intervention. *Clin Appl Thromb Hemost*, *28*, 10760296221081848. <https://doi.org/10.1177/10760296221081848>

Zhang, Z., Zhao, L., Lu, Y., Meng, X., & Zhou, X. (2023). Association between non-insulin-based insulin resistance indices and cardiovascular events in patients undergoing percutaneous coronary intervention: a retrospective study. *Cardiovasc Diabetol*, *22*(1), 161. <https://doi.org/10.1186/s12933-023-01898-1>

Zheng, H., Foo, L. L., Tan, H. C., Richards, A. M., Chan, S. P., Lee, C. H., Low, A. F. H., Hausenloy, D. J., Tan, J. W. C., Sahlen, A. O., Ho, H. H., Chai, S. C., Tong, K. L., Tan, D. S. Y., Yeo, K. K., Chua, T. S. J., Lam, C. S. P., & Chan, M. Y. (2019). Sex Differences in 1-Year Rehospitalization for Heart Failure and Myocardial Infarction After Primary Percutaneous Coronary Intervention. *Am J Cardiol*, *123*(12), 1935-1940. <https://doi.org/10.1016/j.amjcard.2019.03.021>

1. The analysis might be wrong. [↑](#footnote-ref-1)
